# Preference of Chinese clinical researchers to participate in international clinical research training: a cross-sectional study

**DOI:** 10.1186/s12909-025-07570-4

**Published:** 2025-08-30

**Authors:** Yun Chen, Shengjie Zhu, Haoqing Zhu, Pei Li

**Affiliations:** 1 Jiahui Medical Research and Education Group, Shanghai, China; 2 New York University Langone Vilcek Institute of Graduate Biomedical, Sciences’ 25, New York, US; 3 Institute for Hospital Management, Tsinghua University, Beijing, China

**Keywords:** Clinical research training, International medical education, Randomized controlled trials (RCTs), Cross-sectional survey

## Abstract

**Background:**

Systematic and internationally standardized clinical research training has not traditionally been widely accessible. With the growing volume and improving quality of clinical research in China, a pressing question facing Chinese clinical investigators is how to enhance the global impact of their research outputs. This study seeks to examine the needs and preferences of Chinese clinical researchers with respect to international clinical research training.

**Methods:**

An online questionnaire was distributed through the “*NEJM Medical Frontiers*” WeChat platform. The survey collected data on participants’ demographic characteristics, preference to participate in international clinical research, and their training needs and preferences. This study adopted a non-probability sampling approach, primarily utilizing a WeChat public platform to disseminate the questionnaire and invite user participation within a defined time frame. To further expand the sample, a peer referral strategy was employed, whereby participants were encouraged to share the survey with their contacts. Univariate and multivariate analyses were performed to identify factors associated with clinical researchers’ preferences for international training.

**Results:**

Between January 11 and February 7, 2023, 324 questionnaires were completed, of which 312 were valid(physicians (66.7%, *n* = 208), with the remainder from universities, research institutes, or pharmaceutical-related companies (33.3%, *n* = 104)). Overall, 247 respondents (78.9%) expressed interest in international clinical research training. Those with a doctoral degree and first-author experience in Science Citation Index (SCI) publications (68.4%, *n* = 121, *P* = 0.040), particularly those with 1–9 publications (72.2%, *n* = 109, *P* = 0.028), were significantly more likely to show interest. These respondents also prioritized learning clinical research design methods (mean score: 7.28 vs. 6.52, *P* = 0.009), especially randomized controlled trials (*n* = 118, *P* = 0.048).

**Conclusion:**

The findings suggest a potential demand for high-quality international clinical research training among certain groups of Chinese clinical researchers. Respondents with higher educational qualifications and SCI publication experience appeared more likely to express interest in engaging in rigorous research and in participating in international training programs. Their training priorities were primarily related to study design, with particular emphasis on the conduct of large-scale randomized controlled trials(RCTs).

**Supplementary Information:**

The online version contains supplementary material available at 10.1186/s12909-025-07570-4.

## Background

In recent years, China’s medical education has undergone significant reform, evolving to align with contemporary needs through lifelong learning principles [1]. Future medical schools are expected to focus more on the translation of medical innovations into national policies and clinical practice, thereby fostering closer integration between clinical research and practice [2].

International clinical research training is a core element of China’s medical education reform and a global priority [3]. Many developing countries have also embraced global clinical research training to advance their medical systems [4–6]. While strengthening China’s global research impact and fostering international collaboration, such training plays a vital role in achieving these objectives [7]. Despite these advancements, clinical researchers in China have historically had limited access to clinical research education [6]. As globalization accelerates, there is growing recognition of the importance of physicians’ global competence [8]. Within this context, Chinese clinical researchers face both the responsibility and the opportunity to enhance their skills, access international research resources, and promote Chinese medical research on a global stage. The growing importance of international clinical research training cannot be overstated. Such training is vital for developing medical researchers with an international perspective and for enhancing the research capabilities of clinical researchers [9]. To address this need, it is imperative to provide clinical physicians in China with training programs that meet international standards [10].

International experience has shown that differentiated and modular training systems are essential for building clinical research capacity [11]. Although institutions such as the U.S. National Institutes of Health (NIH) [12] and the European Clinical Research Infrastructure Network (ECRIN) [13] are global leaders in clinical research methodology education, relevant investigations have revealed that even in these regions, the lack of systematic and unified training strategies remains a challenge.

Meanwhile, countries and regions in Asia—including Japan, Singapore, and Hong Kong—have also invested resources and actively explored structured approaches to clinical research training [14–16]. These efforts vary in form: some have established structured curricula that integrate interdisciplinary content such as medicine, ethics, scientific writing, project management, and clinical practice [12, 13, 16]; others have introduced internationally recognized clinical research systems to enhance local researchers’ practical competencies [14, 15]; and still others have developed modular programs tailored to specific roles (e.g., principal investigators [PI], clinical research coordinators [CRC], clinical research associates [CRA]) and disease areas [17], in order to meet the diverse professional development needs of clinical researchers.

This study aims to provide exploratory insights to support the development of a localized yet internationally standardized clinical research training system in China.

## Methods

### Study design

This is an observational study that employed a cross-sectional survey design. Between January 11 and February 7, 2023, a structured questionnaire was distributed via the NEJM WeChat public platform to invite user participation. In addition, a peer referral strategy was used, encouraging platform operators, academic researchers, and other relevant individuals to share the survey with their medical research peers. The study aimed to assess the tendency of Chinese clinical researchers who follow NEJM Medical Frontiers to participate in international clinical research training and to identify factors associated with this preference. Data were collected through an online platform using a structured questionnaire.

### Definition of key variables

The questionnaire consisted of several sections: demographic information (e.g., age, gender, highest education level, hospital type such as tertiary hospitals, and work location), research background, training needs and preferences, and open-ended questions to capture participants’ specific expectations and suggestions regarding international training programs (**please refer to Attachment 1 for details)**.

The key variable, “preference for international training,” was defined based on participants’ preferences for training instructors. The respondents were asked whether they preferred domestic or international instructors. Those who selected instructors from leading international universities or editors/editorial board members of top-tier international journals were categorized as “preference for international training.” Based on this criterion, participants were divided into two groups: those with a preference for international training and those without.

The participants were also asked to identify their preferred training topics via a matrix question, with options such as study design, protocol development, statistical analysis, and ethical guidelines. To facilitate quantitative analysis, the responses were scored via a reverse ranking system. The first choice received 8 points, the second choice received 7 points, and so on, with the eighth choice receiving 1 point. Unselected items were treated as missing values and assigned a score of 0. This scoring approach enabled the quantification of participants’ preferences for various research topics and allowed for further analysis of how these preferences influenced their preference for international training.

### Participant recruitment

The study recruited clinical researchers and researchers working in hospitals, regardless of their prior experience with clinical research. The survey link was distributed via the *NEJM Medical Frontiers* WeChat public account, a platform that shares updates from the *New England Journal of Medicine (NEJM)* and highlights recent clinical research in China. This platform has an audience of approximately 500,000 clinical researchers, researchers, and medical professionals. The survey link was shared with eligible individuals through group messages.

**Fig. 1:**
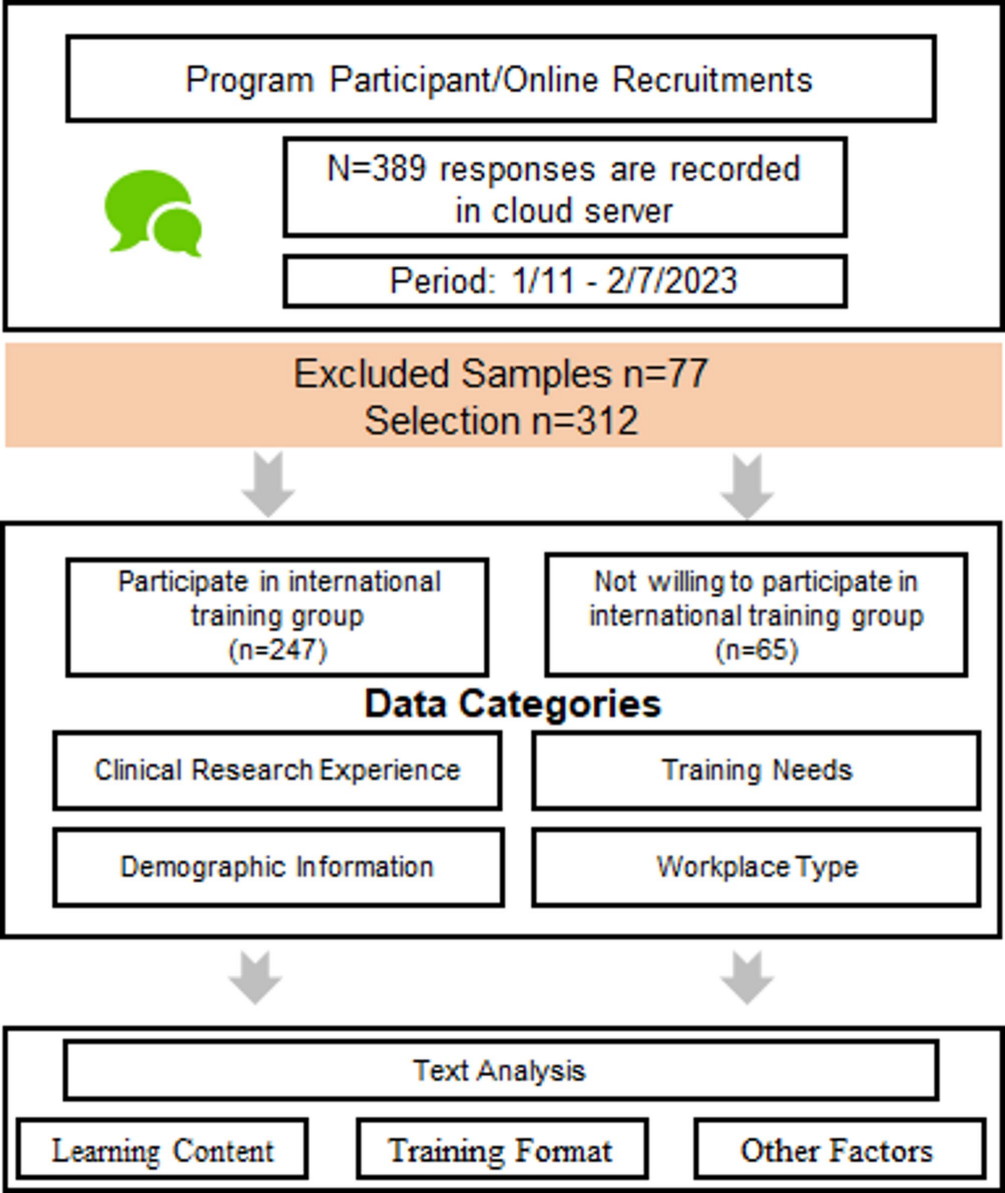
*Elective reporting inclusion flowchart n = number of reports

Between January 11 and February 7, 2023, a total of 324 completed questionnaires were received, of which 312 were deemed valid (Fig. 1).

Additionally, some physicians who were aware of the study further disseminated the survey link, making it difficult to determine the total number of recipients. The participants accessed the questionnaire by scanning a QR code and were required to provide informed consent before completing the survey. The data collected included participants’ clinical research experience and training needs. Owing to the inability to track the total number of nonrespondents, the response rate was not calculated.

### Statistical analysis

Data analysis involved a combination of methods. Descriptive statistics were used to summarize the data, with continuous variables presented as the means ± standard deviations and categorical variables presented as frequencies (percentages). Chi-square tests or t tests were employed to examine the relationships between baseline characteristics (e.g., years of research experience, age, education level, hospital type, and work location) and physicians’ preference for international clinical research training. A p value of < 0.05 was considered to indicate statistical significance.

Factors influencing preference to participate in international clinical research training were identified using unconditional logistic regression analysis. Due to the large number of potential independent variables, logistic regression was conducted in two steps: first, a univariate logistic regression analysis was performed to screen for candidate variables; subsequently, variables with statistically significant (or marginally significant) results in the univariate analysis were entered into a multivariate logistic regression model (forward stepwise method, Wald test, α = 0.05). In the multivariate logistic regression analysis, the dependent variable was defined as preference to participate in international clinical research training (yes/no).

All statistical analyses were performed using SPSS software version 27.0 (IBM Corp., NY, USA).

We conducted a qualitative analysis to explore the training preferences and expectations of users interested in international clinical research training. Data were collected through an open-ended online questionnaire distributed to individuals who had previously engaged with the training program. Participants provided free-text responses regarding their needs, expectations, and preferences.

All responses were first manually reviewed, and an inductive thematic framework was developed. The final framework included three major dimensions: (1) **Learning Content** (e.g., preferred research types, disease areas, balance between theory and practice, need for real-world case studies); (2) **Training Format** (e.g., online/offline delivery, preferred language, demand for repeatable access); and (3) **Other Factors** (e.g., cost considerations, certification needs).

After data cleaning, we applied **ChatGPT-4o** (OpenAI, 2024) to assist with text coding and thematic classification based on the predefined framework. Multiple prompt iterations were used to verify consistency and improve the clarity of identified themes. Final outputs were reviewed by two researchers with clinical research training backgrounds, who validated the AI-generated themes against the original responses to ensure **accuracy**,** coherence**,** and contextual relevance**. Any discrepancies were resolved through discussion.

## Results

### Core dependent variable

Among the 324 distributed questionnaires, 312 valid responses were collected. The average time to complete the questionnaire was approximately five minutes. In terms of training preferences, **78.9% (***n*** = 247)** of the respondents expressed a preference for international instructors, whereas only **20.8% (***n*** = 65)** indicated a preference for domestic instructors (Table 1).

**Table 1:** Distribution of respondents’ preference for international training

Preference for International Training	Number	Percentage
Preference for international instructors	247/312	78.9%
Preference for domestic instructors	65/312	20.8%

### Demographic characteristics

The majority of the participants were aged 31–40 years (54.3%, *n* = 170). Half of them (50.5%, *n* = 158) had less than three years of clinical research experience, while 18.8% (*n* = 59) had 3–5 years, and 19.2% (*n* = 60) had 5–10 years. Most participants (66.3%, *n* = 207) were affiliated with hospitals, predominantly tertiary Grade A hospitals (95.2%, *n* = 197), whereas the remaining participant were from universities, research institutes, or medical enterprises.

Preference to participate in international training varied by age and research experience but did not reach statistical significance. Among the age groups, preference was highest in respondents aged 31–40 years (81.8%) and > 51 years (81.8%), whereas it was lowest in those ≤ 30 years (73.0%; *P* = 0.499).

Interest in participating in international training varied significantly by highest education level (***P* = 0.010**). The respondents with a doctorate demonstrated the highest level of interest (**53.4%**), followed by those with a master’s degree (**42.1%**). The participants with an undergraduate degree had the lowest level of interest (**4.5%**) (Table 2).

**Table 2:** Age and work location with preference for international training

Characteristic	No (%)	Yes (%)	*P* value
**Age**			0.499^1^
≤ 30 years	17 (27.0%)	46 (73.0%)	
31–40 years	31 (18.2%)	139 (81.8%)	
41–50 years	13 (22.8%)	44 (77.2%)	
>51 years	4 (18.82%)	18 (81.8%)	
**Work Location**			0.058^1^
First-tier cities	31 (17.4%)	147 (82.6%)	
Other cities	34 (25.4%)	100 (74.6%)	
**Highest Education Level**			0.010^1*^
Undergraduate	9 (45.0%)	11 (55.0%)	
Master’s	30 (22.4%)	104 (77.6%)	
Doctorate	26 (16.5%)	132 (83.5%)	

1. ^1^Two-sided Pearson chi-square test

2. ^1*^p-value ≤ 0.05

Most respondents were physicians (**66.7%**, ***n* = 208**), with the rest coming from universities, research institutes, or pharmaceutical-related companies (**33.3%**, ***n* = 104**).

Among clinical researchers in hospitals (***n* = 208**), preferences were analyzed by hospital type (Table 3). Respondents from tertiary hospitals made up the majority. Of these, **78.2%** (*n* = 154) indicated a positive attitude, while **21.8%** (*n* = 43) expressed a lack of interest. Among researchers from other hospital types, **90.9%** (*n* = 10) had a positive response, although the sample size was considerably smaller. The association between hospital type and response was not statistically significant (***P* = 0.314)**.

**Table 3:** Hospital type and interest in international training (*n* = 208)

Characteristic	No (%)	Yes (%)	*p* value
**Hospital Type**			0.314^1^
Tertiary	43 (21.8%)	154 (78.2%)	
Others	1 (9.1%)	10 (90.9%)	

1. ^1^Two-sided Pearson chi-square test

### Research experience

The analysis revealed no statistically significant association between years of research experience and response (***P* = 0.207**). Respondents with **5–10 years** and **> 10 years** of research experience had the highest positive response rates (**86.7%** and **86.1%**, respectively), whereas those with **≤ 3 years** and **3–5 years** of research experience presented slightly lower rates (**76.4%** and **74.6%**, respectively).

Similarly, PI status was not significantly related to response (***P* = 0.900**). The respondents who had served as principal investigators (PIs) reported a positive response rate of **79.0%**, nearly identical to those without PI experience (**79.7%**) (Table 4).

**Table 4:** Research experience and interest in international Training(*n* = 312)

Characteristic	Not Willing (%)	Willing (%)	*P* value
**Years of Research**			0.207^1^
≤ 3 years	37 (23.6%)	120 (76.4%)	
3–5 years	15 (25.4%)	44 (74.6%)	
5–10 years	8 (13.3%)	52 (86.7%)	
> 10 years	5 (13.9%)	31 (86.1%)	
**PI Status**			0.9001^1^
No	14 (20.3%)	55 (79.7%)	
Yes	51 (21.0%)	192 (79.0%)	

1. ^1^Two-sided Pearson chi-square test

For SCI publication status (*n* = 223), respondents with SCI publications were more likely to respond positively (82.5%, *n* = 151) than were those without SCI publications (65.0%, *n* = 26). Conversely, respondents without SCI publications were more likely to be unwilling (35.0%, *n* = 14 vs. 17.5%, *n* = 32 for those with SCI publications). This association was statistically significant (***P* = 0.013)**.

First author status (*n* = 223) also revealed significant differences **(***P*** = 0.040)**. The respondents who served as first authors presented a higher positive response rate (83.4%, *n* = 121) than did those without first author experience (71.8%, *n* = 56).

The number of publications (*n* = 183) further highlighted significant differences **(*P* = 0.028)**. The respondents with ≥ 10 publications had the highest positive response rate (93.3%, *n* = 42), whereas those with 1–9 publications had a slightly lower rate (79.0%, *n* = 109). Additionally, the reluctance to participate was significantly lower among those with ≥ 10 publications (6.7%, *n* = 3) than among those with 1–9 publications (21.0%, *n* = 29) (Table 5).

**Table 5:** The relationship between academic performance and the preference to accept international clinical training

Characteristic	No (%)	Yes (%)	*P* value
**SCI Publication(** ***n*** ** = 223)**			0.013^1*^
No	14 (35.0%)	26 (65.0%)	
Yes	32 (17.5%)	151 (82.5%)	
**First Author Status(** ***n*** ** = 223)**			0.040^1*^
No	22 (28.2%)	56 (71.8%)	
Yes	24 (16.6%)	121 (83.4%)	
**Number of Publications(** ***n*** ** = 183)**			0.028^1*^
1–9	29 (21.0%)	109 (79.0%)	
≥ 10	3 (6.7%)	42 (93.3%)	

1. ^1^Two-sided Pearson chi-square test

2. 1* p value ≤ 0.05

### Association between participated research types and learning preferences

Participation in research types showed varying associations with interest in international training. The positive response rate was higher among those involved in single-center randomized controlled trials (84.4%) compared to those not involved (75.5%, *P* = 0.059). Similarly, the positive response rate was higher among respondents who contributed to case report publications (87.6%) than among those who did not contribute to such publications **(75.3%**, ***P* = 0.013)**.

Participation in cross-sectional studies showed the strongest association (*P*** < 0.001)**, with 89.8% of participants responding positively versus 73.5% of nonparticipants. In contrast, involvement did not significantly differ between prospective cohort studies (*P* = 0.407) and case‒control studies (*P* = 0.897) (Table 6).

**Table 6:** The relationship between previously participated research types and preference for international training

Characteristic	Not Willing (%)	Willing (%)	*P* value
**Participated in a single-center randomized controlled trial.**			0.059^1^
No	45(24.5%)	139(75.5%)	
Yes	20(15.6%)	108(84.4%)	
**Participated in a prospective cohort study.**			0.407^1^
No	39(22.5%)	134(77.5%)	
Yes	26(18.7%)	113(81.3%)	
**Participated in a case‒control study.**			0.897^1^
No	39(21.1%)	146(78.9%)	
Yes	26(20.5%)	101(79.5%)	
**Participated in a cross-sectional study.**			<0.001^1*^
No	54(26.5%)	150(73.5%)	
Yes	11(10.2%)	97(89.8%)	
**Contributed to the publication of a case report.**			0.013^1*^
No	53(24.7%)	162(75.3%)	
Yes	12(12.4%)	85(87.6%)	

1. ^1^Two-sided Pearson chi-square test

2. ^1*^ p value ≤ 0.05

### Multivariate logistic regression analysis

In the multivariate logistic regression analysis, no independent factors were significantly associated with physicians’ preference to participate in international clinical research training. Specifically, work location (OR = 0.765, 95% CI: 0.390–1.500, *P* = 0.435), highest education level (OR = 1.604, 95% CI: 0.872–2.949, *P* = 0.129), prior SCI publication history (OR = 1.564, 95% CI: 0.575–4.255, *P* = 0.381), first author status on SCI-indexed articles (OR = 1.247, 95% CI: 0.514–3.021, *P* = 0.626), participation in cross-sectional studies (OR = 1.064, 95% CI: 0.902–1.254, *P* = 0.463), and contribution to published case reports (OR = 1.080, 95% CI: 0.926–1.260, *P* = 0.326) were not statistically significant predictors of the preference for international clinical research training (Table 7). However, a trend suggesting a possible positive association was observed for higher education levels and prior SCI publication history, warranting further investigation with larger sample sizes.

**Table 7:** Multivariate analysis of factors influencing clinical researchers’ preference for participating in international clinical research training

	OR	(95%CI)	*P*
Work Location	0.765	(0.390–1.500)	0.435
Highest Education Level	1.604	(0.872–2.949)	0.129
**SCI Publication**	**1.564**	**(0.575–4.255)**	**0.381**
**First Author Status**	**1.247**	**(0.514–3.021)**	**0.626**
**Participated in a cross-sectional study.**	**1.064**	**(0.902–1.254)**	**0.463**
**Contributed to the publication of a case report.**	**1.080**	**(0.926–1.260)**	**0.326**

### The impact of research type participation and preferences on preference for international training

Participation in randomized controlled trials was significantly associated with preference for international training (***P* = 0.048**), with **80.3%** Yes and **19.7%** No. For other research types, no significant associations were observed: preference was highest for prospective cohort studies (**81.7%**) and moderate for case‒control studies (**68.0%**). Cross-sectional studies and case reports reported lower preference rates (**50.0%** and **66.7%**, respectively) (Table 8).

**Table 8:** Preferred clinical research topics for learning

	No (%)	Yes (%)	*P* value
Randomized Controlled Trials	29(19.7%)	118(80.3%)	**0.048** ^1*^
Prospective Cohort Studies	21(18.3%)	94(81.7%)	
Case‒Control Studies	8(32.0%)	17(68.0%)	
Cross-Sectional Studies	5(50.0%)	5(50.0%)	
Case Reports	2(33.3%)	4(66.7%)	

1. Two-sided Pearson chi-square test

2. ^*^p value ≤ 0.05

Compared with those who were not willing, those willing to participate in international training scored significantly higher in study design (7.28 vs. 6.52, *P* = 0.009) and literature review (1.79 vs. 1.11, *P* = 0.018). Other topics, such as protocol writing, statistical analysis, and execution and management, were not significantly different. A marginal trend was observed in execution and management (*P* = 0.079), with higher scores among those who were not willing (Table 9).

**Table 9:** Comparison of learning preferences for research topics between groups with and without preference for international training

	Not Willing (%)	Willing (%)	t	*P* value
Study Design	6.52	7.28	-2.629	**0.009** ^*^
Protocol Writing	5.11	5.42	-0.856	0.393
Statistical Analysis	4.00	4.45	-1.118	0.265
Ethics and Regulations	2.18	2.39	-0.601	0.548
Execution and Management	4.48	3.81	1.761	0.079
Writing and Publishing	2.22	2.74	-1.520	0.129
Literature Review	1.11	1.79	-2.380	**0.018** ^*^

1. ^*^p-value ≤ 0.05

### Text analysis results

Respondents demonstrated diverse needs regarding training content. While most clinicians expressed interest in randomized controlled trials (RCTs), some noted that observational studies, case-control studies, and cohort designs are more feasible in primary or secondary hospital settings and hoped to gain relevant knowledge through training. A few respondents also recommended including disease-specific modules, such as those focused on infectious diseases, chronic conditions, or rare diseases. Overall, participants preferred a practice-oriented teaching model, emphasizing the inclusion of real-world case studies, simulation exercises, and detailed guidance on research design, data analysis, and protocol development. One respondent noted, *“It would be helpful to explain current examples and make statistical knowledge easier to understand*,*”* while another commented, *“Training should start from research examples*,* using case-based teaching and group discussion.”* These suggestions indicate a strong demand, particularly among grassroots and early-career physicians, for training programs that are both systematic and highly practical.

In terms of training format, flexibility and accessibility emerged as core themes. Most respondents favored online or hybrid models to accommodate the unpredictable schedules of clinical work. Many emphasized the need for repeatable access, such as recorded lectures or modular content that supports self-paced learning. Some participants also suggested incorporating bilingual instruction (Chinese-English) to improve international communication skills, and emphasized the value of ongoing post-course support—such as Q&A groups or one-on-one mentorship. For instance, one respondent remarked, *“Training content should be repeatable*,* with opportunities for learners to ask follow-up questions.”* Another suggested, *“Short-term or weekend in person sessions would be helpful*,* as they wouldn’t interfere with daily clinical duties.”* These responses highlight the importance of balancing flexibility and continuity in training delivery.

Beyond content and format, cost and certification were identified as key factors influencing participation. Many respondents hoped the program would remain affordable and suggested offering partially subsidized or low-cost modules to broaden accessibility. Others emphasized the importance of official certification, which could support professional development or institutional recognition. Several participants remarked, *“The price should not be too high*,*”* and *“Reasonable fees would make the program more inclusive.”* Another noted, *“A certificate upon completion would increase my motivation to attend.”* Taken together, these findings reflect current expectations among clinicians regarding training content, format, and access. They also suggest that future programs should strike a balance between practicality, flexibility, and equity, while aligning with international standards to meaningfully support the research capacity of Chinese physicians.

## Discussion

This study was conducted as a cross-sectional survey aimed at exploring the preferences of Chinese clinical researchers for structured international clinical research training.

The research team primarily comprises members of NEJM Medical Frontiers, an academic platform co-founded by The New England Journal of Medicine (NEJM) and the Jiahui Medical Research and Education Group (J-Med). The platform is dedicated to enhancing the research capabilities of Chinese clinicians by introducing NEJM Group’s exclusive authorized content. It operates through content dissemination, in-person workshops, online courses, and academic conferences, and currently engages approximately 500,000 subscribers with an interest in clinical research.

In this study, the questionnaire was disseminated through a combination of WeChat Official Account posts, Moments sharing, and group messaging, forming an interest-driven, non-probability sampling pathway. The official account served as the primary entry point, Moments enabled social network-based diffusion, and WeChat groups facilitated targeted outreach and interactive dissemination. While this approach allowed for rapid access to a clinically engaged population, it is well-suited for survey mobilization and sample acquisition under resource-limited conditions, particularly when the target population is relatively concentrated and shares a high degree of interest relevance.

Based on user geolocation statistics available through the WeChat platform, most users are concentrated in Guangdong, Beijing, Shanghai, and Jiangsu. Approximately 56.83% of users are from Tier 1 and Tier 2 cities, while about 35% are from other types of cities, and fewer than 10% are from overseas or smaller locales. Among them, roughly 4% (~ 20,000) are considered active users—defined as those who regularly read platform content and engage through likes, bookmarks, comments, or other interactive behaviors. Notably, the distribution of geographic origin among active users is similar to that of the overall user base.

In this survey, the questionnaire was distributed through posts on a WeChat public account targeting active users, who participated on a voluntary basis. In addition, members of the research team promoted the survey via their professional networks to increase outreach and access a broader group of clinical researchers. This approach enabled rapid data collection with reasonable granularity. As of May 2025, the survey article had received 3,430 reads, and we collected 389 questionnaire responses prior to data cleaning. The cooperation rate—the ratio of valid responses to survey viewers—was approximately 11.34%.

While this response rate is lower than typically ideal for cross-sectional studies, potentially introducing nonresponse bias and limiting the representativeness and generalizability of the findings, we observed that the geographic distribution of respondents was consistent with both the active user base and the broader platform user population. Therefore, we believe that the findings retain interpretive value despite this limitation.

Nevertheless, because NEJM Medical Frontiers inherently attracts individuals with a strong interest in clinical research, this sampling strategy may have resulted in selection bias—likely overestimating the level of interest and preference for international clinical research training in the general physician population. Future studies could enhance representativeness by adopting a multi-channel recruitment approach, such as engaging medical associations, academic conferences, and probabilistic sampling methods. The use of incentives and refined questionnaire design may also improve response rates and reduce bias.

Another major limitation of this study lies in the imprecise definition of the primary dependent variable and limited description of influencing factors. We operationalized “preference for international clinical research training” narrowly as the selection of international medical school mentors or international journal editors as desirable training instructors. This definition does not fully capture the multidimensional nature of training preference. In addition to individual-level characteristics (e.g., educational background, prior research experience), a range of contextual factors—including training costs, location, scheduling, language, format, and evaluation methods—could substantially influence respondents’ preferences. For instance, policies and institutional attitudes toward continuing medical education vary significantly across hospitals in China, which in turn affect clinicians’ access to training opportunities [18].

In terms of independent variables, several factors such as educational attainment, research experience, and SCI publication history were significantly associated with training preference in univariate analyses but lost statistical significance in the multivariable logistic regression model. This suggests that while these variables may appear correlated with the outcome in isolation, their effect may be confounded or mediated by other factors, such as academic background, professional environment, or combined research exposure. In other words, these variables may not act as independent predictors of preference, and their relationship to training interest is potentially indirect or conditional.

Future studies may benefit from investigating interaction effects among predictors or applying path analysis or structural equation modeling to better understand the underlying mechanisms. Moreover, employing pre-tested, refined instruments with validated scales would enable more accurate assessment of Chinese clinical researchers’ true preferences and needs regarding international training programs.

On the other hand, although these findings must be interpreted with caution, several observed trends align with our routine experience in clinical education [3, 4, 6, 13]. For instance, higher educational attainment was associated with stronger training preference [21]. We believe that the increased preference among physicians with doctoral degrees reflects the common academic background of Chinese clinical researchers. Those holding doctoral degrees are often responsible for designing and implementing research projects. Their doctoral training typically includes structured research methodology, and after graduation, they continue to play central roles in research activities.

Similarly, physicians with some experience in publishing but without high-impact outputs may have accumulated foundational knowledge through repeated manuscript submission and publication [19–21]. At this career stage, structured training may help them overcome research bottlenecks and pursue greater academic advancement. Therefore, international training may be viewed as a meaningful opportunity to support their research development [22].

Physicians from first-tier cities such as Beijing, Shanghai, and Guangzhou exhibited greater preference for international training, likely due to a combination of higher research pressure and greater availability of resources [23]. These findings are consistent with the explanatory framework of **Self-Determination Theory (SDT)** [24], which posits that preference for participation in professional development programs may not solely depend on access to training opportunities. Rather, future training programs should be intentionally designed to support learners’ basic psychological needs—such as providing flexible and autonomous learning pathways, tiered content to enhance competence, and collaborative learning environments to foster professional connection. These features are more likely to activate intrinsic motivation and promote broader and more sustained participation.

Additional noteworthy findings from this study relate to training content. *The New England Journal of Medicine (NEJM)* is widely recognized as a global leader in large-scale randomized controlled trial (RCT) research [20, 23]. The strong NEJM brand presence on the *NEJM Medical Frontiers* platform may have led users to associate the training content with RCT methodology even prior to completing the survey. Importantly, RCTs are often regarded as the “gold standard” of clinical research and represent a high-level challenge pursued by advanced investigators due to their resource intensity and complexity [25]. Therefore, our finding that respondents with a preference for international training also showed greater interest in RCTs is reasonable and expected.

Global evidence suggests that differentiated and modular training systems are critical to building clinical research capacity [11]. While institutions such as the U.S. National Institutes of Health (NIH) [12] and the European Clinical Research Infrastructure Network (ECRIN) [13] are considered global leaders in clinical research methodology education, surveys indicate that even in these regions, systematic and unified training strategies remain limited, which may constrain the efficiency of multinational collaboration and the advancement of research quality.

Meanwhile, Asian countries and regions such as Japan, Singapore, and Hong Kong have also actively invested in structured clinical research training and have taken diverse, exploratory approaches [14–16]. These initiatives vary in form: some have developed structured curricula that integrate interdisciplinary content including medical knowledge, research ethics, scientific writing, project management, and clinical practice [12, 13, 16]; others have enhanced domestic research capabilities by introducing international best practices and systems [14, 15]; and still others have built role-specific (e.g., PI, CRC, CRA) or disease-specific modular programs [17] to support professional development tailored to various researcher profiles.

In light of our findings—where physicians with higher education levels, moderate publishing experience, and residence in resource-rich cities expressed stronger preference for international training—we believe China would benefit from incorporating such global practices. Specifically, future training programs should adopt a systematic and tiered design in terms of curriculum structure, modular content, and learner segmentation, thereby promoting more effective capacity-building and enabling deeper international collaboration.

In summary, while this study has several limitations, many of its findings are consistent with our observations in practice. These insights further strengthen our confidence in expanding clinical research training in China. In fact, at the time of writing, the *NEJM High-Impact Clinical Trials Certificate Program* has already been conducted twice in China. The first sessions were held in Beijing and Shanghai, and the second was implemented in collaboration with the First Affiliated Hospital of Sun Yat-sen University in Guangdong. Many of the hypotheses generated from this study have been corroborated through subsequent program delivery. We believe the findings retain meaningful value and look forward to further validating our conclusions with additional data in the future.

### Limitations

This study was conducted via the NEJM Medical Frontiers WeChat public account, where all users reached during the designated survey period were invited to complete the questionnaire. Additionally, participation was encouraged through peer and colleague referrals. While this recruitment strategy may limit the representativeness of the sample, it is likely that both the platform’s user base and the extended network accessed by its operators have a heightened interest in clinical research and international training. Consequently, the findings may overestimate the overall demand for such training programs.

Given this context, the results should not be interpreted as reflective of the broader population of healthcare professionals in China, but rather as indicative of the interests and preferences of a specific subset of highly engaged individuals. Furthermore, the response rate was notably low—312 valid responses out of approximately 500,000 potential users—raising additional concerns regarding selection and nonresponse bias. Despite these limitations, as a cross-sectional survey, the study offers preliminary insights into the training needs and motivations of a targeted group of physicians and clinical researchers.

While the key outcome variable, “preference to participate in international training,” was defined using a single proxy item based on instructor preference, which may not fully capture the multifactorial nature of training decisions. Factors such as cost, delivery mode, institutional support, and time availability were not measured but could significantly influence participation.

To address these limitations, future research should incorporate more representative sampling strategies across diverse platforms, refine variable definitions with multiple items, and include contextual factors influencing training participation. Pilot testing and pre-survey cognitive interviews may also improve instrument validity and respondent understanding.

## Conclusion

This study provides preliminary insights into the preferences and potential needs of Chinese clinical researchers regarding international clinical research training. The findings suggest that individuals with higher educational attainment and prior research experience may be more inclined to participate in such programs, and that those expressing interest in international training are particularly drawn to topics such as randomized controlled trials. Although the study is subject to several limitations—including sampling bias and a narrowly defined outcome variable—the observed trends are generally consistent with practical experience and existing assumptions. As such, the results may serve as a useful reference for the future design and implementation of targeted training initiatives.

## Electronic supplementary material

Below is the link to the electronic supplementary material.

Supplementary Material 1

Supplementary Material 2

## Data Availability

All data generated or analyzed during this study are included in this published article.
