# Supplementary material for: Preference of Chinese clinical researchers to participate in international clinical research training: a cross-sectional study
Source: BMC Med Educ. 2025 Aug 30;25:1226. doi: 10.1186/s12909-025-07570-4 (PMC12398024; doi:10.1186/s12909-025-07570-4)
Supplement: Supplementary file 1 — Supplementary Material 1 [file 12909_2025_7570_MOESM1_ESM.docx]

**Attachment 1: Initial variables (some variables were merged or calculated during the statistical process)**

| **No.** | **Variable Name** | **Variable Labels** |
| --- | --- | --- |
| 1 | Years of participation in clinical research | ≤3 years, 3-5 years, 5-10 years, >10 years |
| 2 | Age | Under 30, 31-40 years-old, 41-50 years-old, 51-60 years-old, >60 years-old |
| 3 | Work city | Text |
| 4 | Highest education level | Bachelor Degree, Academic Master Degree, Professional Master Degree, PhD/MD |
| 5 | Primary job title | Missing/Skip, Hospital (e.g., Chief Physician/Associate Chief/Attending, etc.), University or Institute (e.g., Professor/Researcher/Associate Professor, etc.), Enterprise (e.g., Founder/C-level/Department Manager, etc.), Government (e.g., National/Provincial/City + Department Position), Others (Specify organization type + position) |
| 6 | Hospital nature (if Q5 is selected as 1) | Missing/Skip, Grade A Tertiary Hospital with name, Grade B Tertiary Hospital with name, Secondary Hospital with name, Primary Hospital with name, Private Hospital with name, Others with name |
| 7 | University nature (if Q5 is selected as 2) | Missing/Skip, University with name, Research Institute, Others with name |
| 8 | Company nature (if Q5 is selected as 3) | Missing/Skip, Multi-National Company, Local Big Pharma, Biotech Company, Others with name |
| 9 | Secondary title (if applicable, specify) | Missing/Skip, No secondary position, Hospital (Chief Physician/Associate Chief Physician/Attending Physician, etc.), University and Research Institute (Professors/Researchers/Associate Professors, etc.), Enterprise (Founder/C-level/Department Manager, etc.), Government (National/Provincial/City level + Department), Others (Specify organization type + position) |
| 10 | Research PI (specify the number if applicable) | Missing/Skip, Yes (specify), No |
| 11 | Types of clinical research you have participated in (multi-choice) | Missing/Skip, Multi-center randomized controlled trials, Single-center randomized controlled trials, Prospective cohort studies, Case-control studies, Cross-sectional studies, Case reports, Others |
| 12 | Most desired clinical research type to learn (rank by participation frequency) | Missing/Skip, Multi-center randomized controlled trials, Single-center randomized controlled trials, Prospective cohort studies, Case-control studies, Cross-sectional studies, Case reports, Others |
| 13 | Have you published SCI articles? How many? | Missing/Skip, No, 1-5, 6-10, 11-15, 16-20, >20 |
| 14 | Have you published SCI articles as first or corresponding author? | Missing/Skip, No, 1-5, 6-10, 11-15, 16-20, >20 |
| 15 | In your current work, which clinical research knowledge do you most wish to learn? | Missing/Skip, Randomized controlled trials, Prospective cohort studies, Case-control studies, Cross-sectional studies, Case reports, Others |
| 16 | For your current and future career development, what aspects do you want to strengthen? (rank by importance) | Missing/Skip, Clinical research protocol design, Clinical research protocol writing, Statistical analysis of clinical research data (e.g., SPSS, STATA), Clinical research ethics and regulations, Implementation and management of clinical research projects, Paper writing and publishing, Literature review and analysis, Others |
| 17 | What factors can motivate you to participate in clinical research training? (rank by motivation level) | Certificate or degree, Publish SCI clinical research articles, Obtain continuing education credits, Collaborate with well-known clinical experts, Improve clinical research knowledge, Others |
| 18 | Requirements for instructors’ qualifications | Faculty from top international universities (e.g., Harvard, Stanford), Editors/board members of top international journals, First authors of internationally high-impact articles, Conducted internationally impactful clinical research in China, Senior executives with extensive clinical experience, Others |
| 19 | Any suggestions for the upcoming training programs? | Text |
