# Supplementary material for: Preference of Chinese clinical researchers to participate in international clinical research training: a cross-sectional study
Source: BMC Med Educ. 2025 Aug 30;25:1226. doi: 10.1186/s12909-025-07570-4 (PMC12398024; doi:10.1186/s12909-025-07570-4)
Supplement: Supplementary file 2 — Supplementary Material 2 [file 12909_2025_7570_MOESM2_ESM.pdf]

seria	Time for submission	Time us	sour	Source (	From IP
1	2023/1/11 17:08:23	6s	micr	N/A	114.102.33.21 (Anhui-Maansh
2	2023/1/11 17:09:02	184s	micr	N/A	39.144.45.61 (Shanghai-Shan
3	2023/1/11 17:09:15	113s	micr	N/A	14.150.122.63 (Guangdong - (
4	2023/1/11 17:09:49	8s	micr	N/A	202.120.235.104 (Shanghai-S
5	2023/1/11 17:09:59	163s	micr	N/A	61.135.237.253 (Beijing-Beijin
6	2023/1/11 17:10:09	147s	micr	N/A	58.39.115.220 (Shanghai-Sha
7	2023/1/11 17:10:35	198s	micr	N/A	139.227.12.168 (Shanghai-Sh
8	2023/1/11 17:10:47	250s	micr	N/A	118.242.34.250 (Shanghai-Sh
9	2023/1/11 17:11:49	259s	micr	N/A	36.110.96.254 (Beijing-Beijing
10	2023/1/11 17:11:49	174s	micr	N/A	223.104.220.189 (Sichuan - C
11	2023/1/11 17:11:57	221s	micr	N/A	223.72.64.211 (Beijing-Beijing
12	2023/1/11 17:12:03	173s	micr	N/A	120.244.198.213 (Beijing-Beiji
13	2023/1/11 17:12:13	217s	micr	N/A	120.245.68.233 (Beijing-Beijin
14	2023/1/11 17:12:17	245s	micr	N/A	124.116.26.98 (Shaanxi - Yan
15	2023/1/11 17:12:17	221s	micr	N/A	112.17.97.226 (Zhejiang-Hang
16	2023/1/11 17:12:22	370s	micr	N/A	113.201.132.56 (Shaanxi - Xi'a
17	2023/1/11 17:12:25	11s	micr	N/A	114.254.1.16 (Beijing-Beijing)
18	2023/1/11 17:12:43	218s	micr	N/A	39.144.139.63 (Sichuan-Aba)
19	2023/1/11 17:13:20	13s	micr	N/A	58.247.194.82 (Shanghai-Sha
20	2023/1/11 17:13:26	271s	micr	N/A	221.222.20.135 (Beijing-Beijin
21	2023/1/11 17:14:01	387s	micr	N/A	114.87.69.168 (Shanghai-Sha
22	2023/1/11 17:14:07	181s	micr	N/A	180.168.200.195 (Shanghai-S
23	2023/1/11 17:14:11	299s	micr	N/A	223.104.41.155 (Beijing-Beijin
24	2023/1/11 17:14:25	239s	micr	N/A	117.136.94.29 (Shandong - Ji
25	2023/1/11 17:14:39	175s	micr	N/A	117.136.32.63 (Guangdong - (
26	2023/1/11 17:15:02	398s	micr	N/A	14.150.226.177 (Guangdong -
27	2023/1/11 17:15:48	159s	micr	N/A	125.33.205.20 (Beijing-Beijing
28	2023/1/11 17:15:56	306s	micr	N/A	223.104.30.181 (Xinjiang-Urur
29	2023/1/11 17:16:20	277s	micr	N/A	202.120.235.141 (Shanghai-S
30	2023/1/11 17:16:38	44s	micr	N/A	112.17.247.229 (Zhejiang-Sha
31	2023/1/11 17:16:51	588s	micr	N/A	223.104.41.236 (Beijing-Beijin
32	2023/1/11 17:17:17	413s	micr	N/A	183.242.3.214 (Beijing-Beijing
33	2023/1/11 17:17:38	296s	micr	N/A	112.64.119.222 (Shanghai - S
34	2023/1/11 17:18:28	13s	micr	N/A	183.38.152.185 (Guangdong -
35	2023/1/11 17:18:35	245s	micr	N/A	117.24.23.150 (Fujian-Quanzh
36	2023/1/11 17:18:45	265s	micr	N/A	117.89.215.33 (Jiangsu-Nanjir
37	2023/1/11 17:19:21	302s	micr	N/A	14.26.80.104 (Guangdong - S
38	2023/1/11 17:19:30	10s	micr	N/A	112.224.65.58 (Shandong-Qir
39	2023/1/11 17:19:35	248s	micr	N/A	223.104.165.179 (Zhejiang-Qu
40	2023/1/11 17:19:36	276s	micr	N/A	183.129.214.130 (Zhejiang-Ha
41	2023/1/11 17:21:09	245s	micr	N/A	123.138.70.174 (Shaanxi - Xi'a
42	2023/1/11 17:21:11	660s	micr	N/A	39.144.133.163 (Guangxi - Na
43	2023/1/11 17:21:16	340s	micr	N/A	144.255.82.151 (Shandong-W
44	2023/1/11 17:21:26	125s	micr	N/A	117.136.111.233 (Zhejiang-Ha
45	2023/1/11 17:21:39	307s	micr	N/A	124.160.220.142 (Zhejiang-Ha
46	2023/1/11 17:21:56	328s	micr	N/A	183.76.223.177 (Overseas -- .
47	2023/1/11 17:22:59	703s	micr	N/A	120.192.3.72 (Shandong - Jin
48	2023/1/11 17:23:20	317s	micr	N/A	222.212.94.8 (Sichuan - Chen
49	2023/1/11 17:23:58	10s	micr	N/A	112.96.195.210 (Guangdong -
50	2023/1/11 17:24:25	521s	micr	N/A	183.239.175.36 (Guangdong -
51	2023/1/11 17:27:32	324s	micr	N/A	42.94.53.95 (Gansu - Pinglian
52	2023/1/11 17:29:57	370s	micr	N/A	223.104.204.55 (Shaanxi - Xi'a
53	2023/1/11 17:30:28	214s	micr	N/A	218.108.191.13 (Zhejiang-Har
54	2023/1/11 17:30:44	254s	micr	N/A	112.8.184.188 (Shandong-Tai
55	2023/1/11 17:30:48	312s	micr	N/A	221.200.17.104 (Liaoning-She

56	2023/1/11 17:30:52	272s	micr(	N/A	114.248.91.179 (Beijing-Beijin
57	2023/1/11 17:30:58	180s	micr(	N/A	223.104.210.101 (Shanghai-S
58	2023/1/11 17:31:24	535s	micr(	N/A	222.29.17.123 (Beijing-Beijing
59	2023/1/11 17:32:23	276s	micr(	N/A	223.104.41.76 (Beijing-Beijing
60	2023/1/11 17:33:42	103s	micr(	N/A	120.245.122.220 (Beijing-Beiji
61	2023/1/11 17:34:09	197s	micr(	N/A	39.144.145.180 (Yunnan -- Ku
62	2023/1/11 17:48:10	557s	micr(	N/A	106.91.64.147 (Chongqing-Ch
63	2023/1/11 17:52:14	313s	micr(	N/A	101.86.83.51 (Shanghai-Shan
64	2023/1/11 17:53:33	725s	micr(	N/A	183.192.136.227 (Shanghai-S
65	2023/1/11 17:54:47	247s	micr(	N/A	171.41.239.153 (Hubei -- Jing
66	2023/1/11 17:59:42	16s	micr(	N/A	219.239.107.2 (Beijing-Beijing
67	2023/1/11 18:03:56	307s	micr(	N/A	183.200.209.69 (Shanxi - Xinz
68	2023/1/11 18:06:59	160s	micr(	N/A	125.33.205.20 (Beijing-Beijing
69	2023/1/11 18:08:51	124s	micr(	N/A	223.104.165.227 (Zhejiang-Qu
70	2023/1/11 18:14:12	224s	micr(	N/A	120.239.67.48 (Guangdong - (
71	2023/1/11 18:14:53	397s	micr(	N/A	122.96.32.184 (Jiangsu-Nanjir
72	2023/1/11 18:16:39	405s	micr(	N/A	49.93.80.65 (Jiangsu-Nanjing)
73	2023/1/11 18:16:40	307s	micr(	N/A	106.38.47.91 (Beijing-Beijing)
74	2023/1/11 18:18:35	360s	micr(	N/A	117.136.52.222 (Hubei-Wuhar
75	2023/1/11 18:20:44	281s	micr(	N/A	117.152.98.47 (Hubei -- Tianm
76	2023/1/11 18:21:12	8s	micr(	N/A	114.254.0.11 (Beijing-Beijing)
77	2023/1/11 18:21:30	251s	micr(	N/A	101.82.202.49 (Shanghai-Sha
78	2023/1/11 18:24:04	283s	micr(	N/A	117.136.75.93 (Fujian-Fuzhou
79	2023/1/11 18:25:35	8s	micr(	N/A	101.206.168.248 (Sichuan - C
80	2023/1/11 18:26:17	256s	micr(	N/A	1.199.79.210 (Henan - Zheng:
81	2023/1/11 18:27:51	1942s	micr(	N/A	39.144.102.190 (Jilin -- Chang
82	2023/1/11 18:30:47	8s	micr(	N/A	117.136.88.1 (Changsha, Hun
83	2023/1/11 18:35:12	209s	micr(	N/A	101.229.49.174 (Shanghai-Sh
84	2023/1/11 18:36:14	251s	micr(	N/A	120.228.94.225 (Changsha, H
85	2023/1/11 18:36:22	137s	micr(	N/A	139.215.46.234 (Jilin - Chang(
86	2023/1/11 18:37:30	16s	micr(	N/A	223.166.67.220 (Shanghai-Sh
87	2023/1/11 18:45:55	15s	micr(	N/A	223.167.201.148 (Shanghai-S
88	2023/1/11 18:47:43	21s	micr(	N/A	223.104.51.7 (Fujian-Fuzhou)
89	2023/1/11 18:53:37	331s	micr(	N/A	223.104.213.26 (Shanghai-Sh
90	2023/1/11 18:54:46	403s	micr(	N/A	14.135.192.33 (Ningxia -- Yin(
91	2023/1/11 18:55:37	8s	micr(	N/A	39.144.137.89 (Sichuan - Che
92	2023/1/11 19:01:23	140s	micr(	N/A	117.136.50.139 (Shaanxi - Xi':
93	2023/1/11 19:02:50	13s	micr(	N/A	49.77.218.180 (Jiangsu-Nanjir
94	2023/1/11 19:02:51	166s	micr(	N/A	220.196.194.86 (Shanghai-Sh
95	2023/1/11 19:04:01	344s	micr(	N/A	120.244.166.242 (Beijing-Beiji
96	2023/1/11 19:04:39	14s	micr(	N/A	49.84.118.246 (Jiangsu - Xuzl
97	2023/1/11 19:06:15	168s	micr(	N/A	101.80.22.65 (Shanghai-Shan
98	2023/1/11 19:11:20	168s	micr(	N/A	223.104.150.45 (Jiangsu-Nanj
99	2023/1/11 19:14:34	354s	micr(	N/A	114.254.0.82 (Beijing-Beijing)
100	2023/1/11 19:15:46	271s	micr(	N/A	39.144.103.222 (Shanghai-Sh
101	2023/1/11 19:17:48	265s	micr(	N/A	42.7.120.103 (Liaoning-Huluda
102	2023/1/11 19:18:59	49s	micr(	N/A	101.224.65.32 (Shanghai-Sha
103	2023/1/11 19:20:20	311s	micr(	N/A	115.192.215.104 (Zhejiang-Ha
104	2023/1/11 19:25:52	231s	micr(	N/A	120.243.53.185 (Anhui-Bozho
105	2023/1/11 19:31:23	169s	micr(	N/A	39.130.65.98 (Yunnan -- Kunr
106	2023/1/11 19:33:58	310s	micr(	N/A	27.151.52.81 (Fujian-Fuzhou)
107	2023/1/11 19:39:08	343s	micr(	N/A	119.130.155.67 (Guangdong -
108	2023/1/11 19:41:50	205s	micr(	N/A	223.104.96.55 (Guizhou - Gui
109	2023/1/11 19:44:18	825s	micr(	N/A	120.204.150.147 (Shanghai-S
110	2023/1/11 19:44:49	13s	micr(	N/A	112.10.246.81 (Zhejiang-Hang
111	2023/1/11 19:54:24	12s	micr(	N/A	60.186.78.156 (Zhejiang-Hang

112	2023/1/11 19:56:01	240s	micr(	N/A	223.104.247.204 (Zhejiang-Ha
113	2023/1/11 19:57:56	1235s	micr(	N/A	39.144.39.11 (Shanghai-Shan
114	2023/1/11 20:00:49	408s	micr(	N/A	124.64.22.249 (Beijing-Beijing
115	2023/1/11 20:08:33	29s	micr(	N/A	114.93.3.49 (Shanghai-Shang
116	2023/1/11 20:09:39	355s	micr(	N/A	117.136.38.194 (Beijing-Beijin
117	2023/1/11 20:13:14	296s	micr(	N/A	112.97.49.51 (Guangdong - S
118	2023/1/11 20:17:15	306s	micr(	N/A	183.95.75.155 (Hubei-Wuhan
119	2023/1/11 20:19:08	184s	micr(	N/A	116.234.92.134 (Shanghai-Sh
120	2023/1/11 20:21:28	194s	micr(	N/A	221.196.108.123 (Tianjin-Tian
121	2023/1/11 20:22:42	163s	micr(	N/A	112.22.75.66 (Jiangsu-Wuxi)
122	2023/1/11 20:24:32	19s	micr(	N/A	180.174.130.143 (Shanghai-S
123	2023/1/11 20:26:36	235s	micr(	N/A	115.199.127.220 (Zhejiang-Ha
124	2023/1/11 20:28:17	221s	micr(	N/A	180.174.130.143 (Shanghai-S
125	2023/1/11 20:34:45	191s	micr(	N/A	220.112.95.74 (Shanghai-Sha
126	2023/1/11 20:36:34	474s	micr(	N/A	219.233.181.10 (Shanghai-Sh
127	2023/1/11 20:41:37	14s	micr(	N/A	36.101.147.227 (Hainan -- Ha
128	2023/1/11 20:45:10	207s	micr(	N/A	183.162.177.202 (Anhui-Bozh
129	2023/1/11 20:49:36	189s	micr(	N/A	60.181.74.20 (Zhejiang-Wenz
130	2023/1/11 20:50:00	8s	micr(	N/A	180.174.237.36 (Shanghai-Sh
131	2023/1/11 20:51:38	84s	micr(	N/A	180.174.237.36 (Shanghai-Sh
132	2023/1/11 20:56:36	147s	micr(	N/A	59.33.103.185 (Guangdong -
133	2023/1/11 21:00:10	459s	micr(	N/A	39.144.64.245 (Guangxi -- Liu
134	2023/1/11 21:02:17	3702s	micr(	N/A	180.107.192.85 (Jiangsu-Suzh
135	2023/1/11 21:05:08	195s	micr(	N/A	117.15.202.34 (Tianjin-Tianjin
136	2023/1/11 21:08:35	455s	micr(	N/A	120.245.122.11 (Beijing-Beijin
137	2023/1/11 21:09:21	260s	micr(	N/A	101.87.208.244 (Shanghai-Sh
138	2023/1/11 21:32:31	21s	micr(	N/A	183.195.18.153 (Shanghai-Sh
139	2023/1/11 21:39:40	209s	micr(	N/A	112.224.143.192 (Shandong-u
140	2023/1/11 21:40:23	185s	micr(	N/A	39.173.117.14 (Zhejiang-Hang
141	2023/1/11 21:41:58	352s	micr(	N/A	223.104.76.161 (Guangdong -
142	2023/1/11 21:43:29	256s	micr(	N/A	182.39.52.192 (Shandong - Ji
143	2023/1/11 21:48:23	5s	micr(	N/A	120.229.250.63 (Guangdong -
144	2023/1/11 21:50:13	14s	micr(	N/A	207.237.226.211 (Foreign - U
145	2023/1/11 21:57:39	166s	micr(	N/A	116.232.151.46 (Shanghai-Sh
146	2023/1/11 21:58:13	265s	micr(	N/A	124.240.91.226 (Guangdong -
147	2023/1/11 22:08:07	1153s	micr(	N/A	223.72.77.220 (Beijing-Beijing
148	2023/1/11 22:16:31	184s	micr(	N/A	211.103.227.250 (Beijing-Beiji
149	2023/1/11 22:19:03	399s	micr(	N/A	61.161.198.12 (Liaoning-Sher
150	2023/1/11 22:24:21	182s	micr(	N/A	101.88.209.232 (Shanghai-Sh
151	2023/1/11 22:29:58	277s	micr(	N/A	117.170.100.16 (Jiangxi - Gar
152	2023/1/11 22:30:10	433s	micr(	N/A	223.104.150.12 (Jiangsu-Nanj
153	2023/1/11 22:30:51	201s	micr(	N/A	183.62.48.156 (Guangdong -
154	2023/1/11 22:34:20	297s	micr(	N/A	122.240.40.130 (Zhejiang-We
155	2023/1/11 22:36:33	206s	micr(	N/A	39.130.65.50 (Yunnan -- Kunr
156	2023/1/11 22:40:09	215s	micr(	N/A	223.104.76.230 (Guangdong -
157	2023/1/11 22:42:50	305s	micr(	N/A	223.104.196.80 (Shandong-Ji
158	2023/1/11 22:44:42	152s	micr(	N/A	183.193.38.207 (Shanghai-Sh
159	2023/1/11 22:49:25	14s	micr(	N/A	223.104.42.115 (Beijing-Beijin
160	2023/1/11 22:50:05	14s	micr(	N/A	183.195.25.151 (Shanghai-Sh
161	2023/1/11 22:52:07	16s	micr(	N/A	118.212.207.245 (Jiangxi -- N
162	2023/1/11 22:53:14	105s	micr(	N/A	134.174.250.144 (Foreign - U
163	2023/1/11 22:53:39	9s	micr(	N/A	218.17.126.122 (Guangdong -
164	2023/1/11 22:56:02	189s	micr(	N/A	101.80.19.19 (Shanghai-Shan
165	2023/1/11 23:01:45	206s	micr(	N/A	122.192.15.24 (Jiangsu-Suzh
166	2023/1/11 23:03:58	607s	micr(	N/A	117.176.248.138 (Sichuan - C
167	2023/1/11 23:20:01	10s	micr(	N/A	120.229.13.128 (Guangdong -

168	2023/1/11 23:23:34	129s	micr(	N/A	120.244.218.195 (Beijing-Beiji
169	2023/1/11 23:38:01	197s	micr(	N/A	140.206.199.154 (Shanghai-S
170	2023/1/11 23:38:12	232s	micr(	N/A	183.192.59.74 (Shanghai-Sha
171	2023/1/11 23:41:53	202s	micr(	N/A	120.230.109.177 (Guangdong
172	2023/1/11 23:48:40	2052s	micr(	N/A	116.21.130.49 (Guangdong -
173	2023/1/11 23:55:15	364s	micr(	N/A	117.136.0.248 (Beijing-Beijing
174	2023/1/12 0:02:49	33s	micr(	N/A	223.104.150.22 (Jiangsu-Nanj
175	2023/1/12 0:07:44	23s	micr(	N/A	183.208.196.5 (Jiangsu-Suzho
176	2023/1/12 0:21:54	281s	micr(	N/A	223.104.193.114 (Shandong -
177	2023/1/12 1:28:38	300s	micr(	N/A	65.220.61.226 (Foreign - Unite
178	2023/1/12 1:33:56	233s	micr(	N/A	125.86.108.200 (Chongqing-C
179	2023/1/12 3:49:08	355s	micr(	N/A	183.192.227.182 (Shanghai-S
180	2023/1/12 6:29:56	334s	micr(	N/A	111.199.80.12 (Beijing-Beijing
181	2023/1/12 7:08:36	17s	micr(	N/A	171.218.84.78 (Sichuan - Che
182	2023/1/12 7:09:23	16s	micr(	N/A	39.144.104.40 (Shanghai-Sha
183	2023/1/12 7:14:08	278s	micr(	N/A	39.144.104.40 (Shanghai-Sha
184	2023/1/12 7:18:36	473s	micr(	N/A	123.123.97.128 (Beijing-Beijin
185	2023/1/12 7:42:19	190s	micr(	N/A	114.254.0.186 (Beijing-Beijing
186	2023/1/12 7:46:10	227s	micr(	N/A	223.104.10.60 (Jiangxi -- Nan
187	2023/1/12 7:47:51	311s	micr(	N/A	125.32.13.19 (Jilin - Changchu
188	2023/1/12 8:07:28	309s	micr(	N/A	112.17.235.253 (Zhejiang-We
189	2023/1/12 8:21:39	16s	micr(	N/A	223.104.64.219 (Guangdong -
190	2023/1/12 8:27:05	399s	micr(	N/A	116.178.3.245 (Xinjiang-Urum
191	2023/1/12 8:37:47	378s	micr(	N/A	124.74.137.126 (Shanghai-Sh
192	2023/1/12 8:49:42	170s	micr(	N/A	116.237.192.173 (Shanghai-S
193	2023/1/12 8:52:59	295s	micr(	N/A	58.56.30.222 (Shandong - Jin
194	2023/1/12 9:01:07	269s	micr(	N/A	121.35.183.207 (Guangdong -
195	2023/1/12 9:07:10	191s	micr(	N/A	124.64.22.249 (Beijing-Beijing
196	2023/1/12 9:25:29	17s	micr(	N/A	114.254.1.135 (Beijing-Beijing
197	2023/1/12 9:43:08	12s	micr(	N/A	218.249.94.200 (Beijing-Beijin
198	2023/1/12 10:14:04	177s	micr(	N/A	223.104.192.81 (Shandong - L
199	2023/1/12 10:26:26	198s	micr(	N/A	223.104.76.222 (Guangdong -
200	2023/1/12 10:56:35	32s	micr(	N/A	36.110.58.34 (Beijing-Beijing)
201	2023/1/12 10:59:49	239s	micr(	N/A	36.110.58.34 (Beijing-Beijing)
202	2023/1/12 11:00:33	210s	micr(	N/A	36.110.58.34 (Beijing-Beijing)
203	2023/1/12 11:20:56	1308s	micr(	N/A	223.71.29.86 (Beijing-Beijing)
204	2023/1/12 11:45:40	197s	micr(	N/A	223.104.41.105 (Beijing-Beijin
205	2023/1/12 11:50:35	106s	micr(	N/A	39.144.95.155 (Shanxi -- Taiy
206	2023/1/12 12:03:15	13s	micr(	N/A	111.32.100.182 (Tianjin-Tianji
207	2023/1/12 12:44:05	241s	micr(	N/A	223.104.246.47 (Zhejiang-Har
208	2023/1/12 12:46:48	184s	micr(	N/A	114.241.86.71 (Beijing-Beijing
209	2023/1/12 12:53:50	264s	micr(	N/A	222.71.124.178 (Shanghai-Sh
210	2023/1/12 13:06:08	194s	micr(	N/A	223.104.76.172 (Guangdong -
211	2023/1/12 14:33:15	247s	micr(	N/A	39.129.249.29 (Yunnan -- Kur
212	2023/1/12 16:13:50	228s	micr(	N/A	218.22.1.158 (Anhui-Hefei)
213	2023/1/12 16:16:22	14s	micr(	N/A	113.87.129.40 (Guangdong -
214	2023/1/12 16:44:23	115s	micr(	N/A	112.96.54.178 (Guangdong -
215	2023/1/12 17:44:06	267s	micr(	N/A	112.97.84.124 (Guangdong -
216	2023/1/12 19:36:34	264s	micr(	N/A	182.102.196.191 (Jiangxi -- N
217	2023/1/12 19:57:26	230s	micr(	N/A	223.104.51.210 (Fujian-Fuzho
218	2023/1/12 22:44:56	334s	micr(	N/A	123.149.74.254 (Henan-Zheng
219	2023/1/12 23:59:23	1027s	micr(	N/A	39.144.197.58 (Xinjiang -- Hot
220	2023/1/13 0:07:40	184s	micr(	N/A	183.46.183.174 (Guangdong -
221	2023/1/13 7:42:58	243s	micr(	N/A	119.44.45.96 (Hunan -- Huaih
222	2023/1/13 7:51:24	275s	micr(	N/A	39.144.135.196 (Guangxi - Yu
223	2023/1/13 7:55:41	211s	micr(	N/A	39.144.135.196 (Guangxi - Yu

224	2023/1/13 8:46:46	154s	micr(	N/A	223.104.41.16 (Beijing-Beijing
225	2023/1/13 10:24:32	161s	micr(	N/A	112.17.97.226 (Zhejiang-Hang
226	2023/1/13 10:24:41	182s	micr(	N/A	36.28.129.92 (Zhejiang-Jinhua
227	2023/1/13 10:27:32	157s	micr(	N/A	120.236.205.211 (Guangdong
228	2023/1/13 10:28:14	344s	micr(	N/A	223.104.165.201 (Zhejiang-Qu
229	2023/1/13 12:27:20	222s	micr(	N/A	120.243.141.38 (Anhui-Huaina
230	2023/1/13 15:36:40	108s	micr(	N/A	42.80.31.106 (Tianjin-Tianjin)
231	2023/1/13 15:42:03	292s	micr(	N/A	182.149.162.25 (Sichuan - Ch
232	2023/1/13 15:43:47	13s	micr(	N/A	123.115.145.38 (Beijing-Beijin
233	2023/1/13 15:53:54	724s	micr(	N/A	76.226.70.114 (Foreign - Unite
234	2023/1/13 16:16:26	274s	micr(	N/A	112.41.76.132 (Liaoning-Sher
235	2023/1/13 16:57:24	85s	micr(	N/A	219.239.107.2 (Beijing-Beijing
236	2023/1/13 18:29:29	174s	micr(	N/A	36.48.125.198 (Jilin - Changch
237	2023/1/13 18:58:16	196s	micr(	N/A	106.37.126.231 (Beijing-Beijin
238	2023/1/14 2:46:00	187s	micr(	N/A	183.193.138.90 (Shanghai-Sh
239	2023/1/14 7:00:39	8s	micr(	N/A	223.147.199.54 (Hunan - Hen
240	2023/1/14 7:01:28	13s	micr(	N/A	183.46.180.87(Guangdong-Sh
241	2023/1/14 7:55:32	5s	micr(	N/A	27.223.134.63 (Shandong-Qir
242	2023/1/14 8:17:04	296s	micr(	N/A	101.88.41.174 (Shanghai-Sha
243	2023/1/14 8:53:42	13s	micr(	N/A	180.164.81.235 (Shanghai-Sh
244	2023/1/14 9:03:37	376s	micr(	N/A	218.26.158.136 (Shanxi-Taiyu
245	2023/1/14 9:10:26	224s	micr(	N/A	223.104.42.66 (Beijing-Beijing
246	2023/1/14 9:49:07	421s	micr(	N/A	183.192.123.29 (Shanghai-Sh
247	2023/1/14 9:49:43	398s	micr(	N/A	58.37.94.231 (Shanghai-Shan
248	2023/1/14 10:51:06	477s	micr(	N/A	101.90.11.82 (Shanghai-Shan
249	2023/1/14 11:10:59	7s	micr(	N/A	36.28.29.114 (Zhejiang-Hangz
250	2023/1/14 11:39:46	214s	micr(	N/A	180.110.156.108 (Jiangsu-Na
251	2023/1/14 14:20:56	144s	micr(	N/A	115.171.244.81 (Beijing-Beijin
252	2023/1/14 17:47:43	152s	micr(	N/A	61.149.74.209 (Beijing-Beijing
253	2023/1/14 18:06:16	221s	micr(	N/A	112.96.54.184 (Guangdong - (
254	2023/1/14 18:18:01	38s	micr(	N/A	111.16.78.72 (Shandong-Jinin
255	2023/1/14 18:20:08	325s	micr(	N/A	183.195.45.30 (Shanghai-Sha
256	2023/1/14 18:35:39	394s	micr(	N/A	222.129.55.72 (Beijing-Beijing
257	2023/1/14 19:02:47	14s	micr(	N/A	117.103.129.242 (Beijing-Beiji
258	2023/1/14 19:35:44	1157s	micr(	N/A	223.104.190.155 (Shandong -
259	2023/1/14 19:55:27	198s	micr(	N/A	112.64.68.249 (Shanghai-Sha
260	2023/1/14 20:05:02	372s	micr(	N/A	123.119.117.242 (Beijing-Beiji
261	2023/1/14 20:12:30	382s	micr(	N/A	123.232.209.35 (Shandong - .
262	2023/1/14 21:06:36	171s	micr(	N/A	123.113.105.171 (Beijing-Beiji
263	2023/1/14 21:35:36	170s	micr(	N/A	223.167.141.148 (Shanghai-S
264	2023/1/14 22:05:00	374s	micr(	N/A	101.88.0.157 (Shanghai-Shan
265	2023/1/14 22:55:43	165s	micr(	N/A	113.87.192.199 (Guangdong -
266	2023/1/14 23:24:09	348s	micr(	N/A	114.60.224.115 (Shanghai-Sh
267	2023/1/14 23:39:58	15s	micr(	N/A	183.194.170.71 (Shanghai-Sh
268	2023/1/15 9:14:18	303s	micr(	N/A	120.244.4.40 (Beijing-Beijing)
269	2023/1/15 9:30:13	181s	micr(	N/A	183.209.152.241 (Jiangsu-Na
270	2023/1/15 9:33:48	499s	micr(	N/A	219.237.184.244 (Beijing-Beiji
271	2023/1/15 9:54:58	784s	micr(	N/A	223.72.86.134 (Beijing-Beijing
272	2023/1/15 11:15:12	923s	micr(	N/A	114.222.121.78 (Jiangsu - Na
273	2023/1/15 13:11:03	18s	micr(	N/A	111.173.152.183 (Hubei -- Xia
274	2023/1/15 13:50:03	212s	micr(	N/A	183.192.19.117 (Shanghai-Sh
275	2023/1/15 14:08:34	698s	micr(	N/A	171.219.25.67 (Sichuan - Che
276	2023/1/15 17:30:46	148s	micr(	N/A	180.98.128.225 (Jiangsu-Nanj
277	2023/1/15 17:38:48	27s	micr(	N/A	60.253.155.39 (Beijing-Beijing
278	2023/1/15 17:39:41	207s	micr(	N/A	223.104.214.155 (Sichuan -- Y
279	2023/1/15 17:40:53	162s	micr(	N/A	117.136.52.246 (Hubei-Wuhan

280	2023/1/15 17:55:44	228s	micr	N/A	110.188.56.98 (Sichuan - Che
281	2023/1/15 18:17:00	311s	micr	N/A	223.104.39.121 (Beijing-Beijin
282	2023/1/15 18:27:16	168s	micr	N/A	223.101.29.11 (Liaoning-Sher
283	2023/1/15 19:21:26	130s	micr	N/A	113.132.219.81 (Shaanxi - Xi'
284	2023/1/15 19:35:23	407s	micr	N/A	144.12.209.233 (Shandong-Bi
285	2023/1/15 19:47:42	260s	micr	N/A	183.208.54.214 (Jiangsu-Suzh
286	2023/1/15 21:22:37	8s	micr	N/A	115.192.172.34 (Zhejiang-Har
287	2023/1/15 23:39:41	14s	micr	N/A	111.194.183.252 (Beijing-Beiji
288	2023/1/16 6:33:35	9s	micr	N/A	111.196.210.236 (Beijing-Beiji
289	2023/1/16 10:34:08	211s	micr	N/A	221.219.0.255 (Beijing-Beijing
290	2023/1/16 13:45:37	211s	micr	N/A	101.229.218.183 (Shanghai-S
291	2023/1/19 19:43:50	177s	micr	N/A	113.57.182.224 (Hubei-Wuha
292	2023/1/23 9:02:24	215s	micr	N/A	14.122.128.147 (Guangdong -
293	2023/1/24 14:07:43	375s	micr	N/A	223.104.41.52 (Beijing-Beijing
294	2023/1/28 11:35:21	14s	micr	N/A	223.95.54.203 (Zhejiang-Jinhu
295	2023/1/28 11:35:56	235s	micr	N/A	218.26.55.31 (Shanxi - Xinzho
296	2023/1/28 11:37:03	224s	micr	N/A	39.144.73.237 (Ningxia -- Shiz
297	2023/1/28 11:37:30	214s	micr	N/A	183.9.211.95 (Guangdong - Ji
298	2023/1/28 11:37:52	29s	micr	N/A	223.104.212.185 (Shanghai-S
299	2023/1/28 11:37:57	222s	micr	N/A	223.104.76.205 (Guangdong -
300	2023/1/28 11:39:19	151s	micr	N/A	117.61.28.163 (Beijing-Beijing
301	2023/1/28 11:39:38	338s	micr	N/A	14.111.240.173 (Chongqing-C
302	2023/1/28 11:40:30	307s	micr	N/A	221.222.21.99 (Beijing-Beijing
303	2023/1/28 11:40:45	203s	micr	N/A	116.246.36.58 (Shanghai-Sha
304	2023/1/28 11:41:09	336s	micr	N/A	117.136.30.2 (Chongqing-Cho
305	2023/1/28 11:41:56	217s	micr	N/A	183.195.66.165 (Shanghai-Sh
306	2023/1/28 11:42:18	461s	micr	N/A	14.122.131.185 (Guangdong -
307	2023/1/28 11:42:45	497s	micr	N/A	223.104.210.9 (Shanghai-Sha
308	2023/1/28 11:42:59	184s	micr	N/A	115.231.180.1 (Zhejiang-Ning
309	2023/1/28 11:45:12	164s	micr	N/A	223.246.116.88 (Anhui-Huain
310	2023/1/28 11:48:08	280s	micr	N/A	14.122.150.79 (Guangdong -
311	2023/1/28 11:48:36	15s	micr	N/A	182.240.117.33 (Yunnan - Qu
312	2023/1/28 11:48:56	165s	micr	N/A	114.253.195.24 (Beijing-Beijin
313	2023/1/28 11:51:02	361s	micr	N/A	223.104.40.42 (Beijing-Beijing
314	2023/1/28 11:54:52	247s	micr	N/A	39.144.39.152 (Shanghai-Sha
315	2023/1/28 11:57:56	141s	micr	N/A	223.104.67.69 (Guangdong -
316	2023/1/28 11:58:54	228s	micr	N/A	27.128.63.35 (Hebei -- Shijiaz
317	2023/1/28 11:59:40	171s	micr	N/A	39.144.45.22 (Shanghai-Shan
318	2023/1/28 12:01:58	267s	micr	N/A	139.205.156.79 (Sichuan-Mia
319	2023/1/28 12:02:43	336s	micr	N/A	39.144.103.124 (Shanghai-Sh
320	2023/1/28 12:09:21	259s	micr	N/A	114.250.149.62 (Beijing-Beijin
321	2023/1/28 12:09:23	169s	micr	N/A	211.97.125.86 (Fujian-Fuzhou
322	2023/1/28 12:10:37	156s	micr	N/A	112.224.141.82 (Shandong-ur
323	2023/1/28 12:12:00	376s	micr	N/A	223.96.203.22 (Shandong-Za
324	2023/1/28 12:16:47	275s	micr	N/A	114.249.61.72 (Beijing-Beijing
325	2023/1/28 12:21:57	143s	micr	N/A	223.104.41.171 (Beijing-Beijin
326	2023/1/28 12:23:16	569s	micr	N/A	49.90.179.241 (Jiangsu-Nanji
327	2023/1/28 12:29:12	263s	micr	N/A	223.104.39.11 (Beijing-Beijing
328	2023/1/28 12:33:49	149s	micr	N/A	223.104.150.120 (Jiangsu-Na
329	2023/1/28 12:38:31	175s	micr	N/A	223.104.41.189 (Beijing-Beijin
330	2023/1/28 12:44:28	34s	micr	N/A	222.218.183.110 (Guangxi - B
331	2023/1/28 12:46:40	238s	micr	N/A	117.153.2.61 (Hubei - Suizho
332	2023/1/28 13:08:04	454s	micr	N/A	183.192.124.181 (Shanghai -
333	2023/1/28 13:24:42	7s	micr	N/A	223.104.63.119 (Guangdong -
334	2023/1/28 13:27:18	373s	micr	N/A	112.65.61.248 (Shanghai-Sha
335	2023/1/28 13:30:25	16s	micr	N/A	113.65.34.103 (Guangdong -

336	2023/1/28 13:32:16	331s	micr(	N/A	1.202.68.242 (Beijing-Beijing)
337	2023/1/28 13:34:03	13s	micr(	N/A	221.192.179.204 (Hebei -- Shi
338	2023/1/28 13:41:17	232s	micr(	N/A	222.131.31.77 (Beijing-Beijing
339	2023/1/28 13:45:02	612s	micr(	N/A	203.144.68.189 (abroad -- Ca
340	2023/1/28 13:47:49	163s	micr(	N/A	1.199.76.162 (Henan - Zheng:
341	2023/1/28 13:49:59	180s	micr(	N/A	117.39.251.72 (Shaanxi - Xi'a
342	2023/1/28 14:41:01	21s	micr(	N/A	14.222.41.247 (Guangdong - I
343	2023/1/28 14:55:18	206s	micr(	N/A	111.18.46.58 (Shaanxi - Xi'an
344	2023/1/28 15:28:52	192s	micr(	N/A	36.112.110.215 (Beijing-Beijin
345	2023/1/28 15:31:10	9s	micr(	N/A	106.121.69.183 (Beijing-Beijin
346	2023/1/28 15:33:51	549s	micr(	N/A	117.136.95.144 (Shandong-W
347	2023/1/28 15:38:44	153s	micr(	N/A	223.104.164.101 (Zhejiang-Jia
348	2023/1/28 15:51:40	262s	micr(	N/A	123.147.252.67 (Chongqing-C
349	2023/1/28 16:33:52	21s	micr(	N/A	223.104.250.252 (Chongqing-
350	2023/1/28 16:48:14	156s	micr(	N/A	39.130.115.6 (Yunnan -- Kunr
351	2023/1/28 17:07:21	207s	micr(	N/A	120.228.6.78 (Hunan -- Chang
352	2023/1/28 18:07:17	254s	micr(	N/A	123.185.181.181 (Liaoning-Da
353	2023/1/28 18:22:11	377s	micr(	N/A	117.136.66.115 (Jiangsu - Na
354	2023/1/28 18:53:12	390s	micr(	N/A	111.208.112.95 (Jiangsu-Nanj
355	2023/1/28 19:25:39	251s	micr(	N/A	120.245.126.186 (Beijing-Beiji
356	2023/1/28 20:24:38	203s	micr(	N/A	112.17.247.111 (Zhejiang-Sha
357	2023/1/28 21:00:15	35s	micr(	N/A	115.60.21.152 (Henan-Zheng:
358	2023/1/28 21:21:46	11s	micr(	N/A	120.244.13.48 (Beijing-Beijing
359	2023/1/28 21:28:42	13s	micr(	N/A	124.240.3.24 (Guangdong - G
360	2023/1/28 21:56:08	22s	micr(	N/A	98.35.162.13 (Foreign - USA)
361	2023/1/28 23:08:39	16s	micr(	N/A	117.136.50.139 (Shaanxi - Xi'a
362	2023/1/28 23:10:24	300s	micr(	N/A	125.62.3.201 (Chongqing-Cho
363	2023/1/28 23:18:46	170s	micr(	N/A	218.20.9.24 (Guangdong - Gu
364	2023/1/28 23:59:48	7s	micr(	N/A	220.173.190.31 (Guangxi -- Li
365	2023/1/29 1:04:40	188s	micr(	N/A	27.18.219.144 (Hubei-Wuhan
366	2023/1/29 7:36:47	513s	micr(	N/A	119.164.216.130 (Shandong -
367	2023/1/29 8:16:03	31s	micr(	N/A	114.216.67.19 (Jiangsu-Suzh(
368	2023/1/29 8:22:54	250s	micr(	N/A	114.233.81.172 (Jiangsu-Taiz
369	2023/1/29 8:31:14	392s	micr(	N/A	113.98.246.227 (Guangdong -
370	2023/1/29 9:09:20	215s	micr(	N/A	223.72.41.176 (Beijing-Beijing
371	2023/1/29 9:53:39	237s	micr(	N/A	223.104.41.75 (Beijing-Beijing
372	2023/1/29 13:06:52	182s	micr(	N/A	112.96.132.105 (Guangdong -
373	2023/1/29 13:17:43	199s	micr(	N/A	39.144.84.221 (Hebei -- Xingt:
374	2023/1/29 13:38:51	229s	micr(	N/A	36.98.100.101 (Hebei - Langf:
375	2023/1/29 21:02:04	472s	micr(	N/A	114.249.63.108 (Beijing-Beijin
376	2023/1/29 22:31:18	335s	micr(	N/A	115.171.168.73 (Beijing-Beijin
377	2023/1/30 7:47:15	279s	micr(	N/A	171.219.76.238 (Sichuan - Ch
378	2023/1/30 8:20:48	224s	micr(	N/A	39.144.104.104 (Shanghai-Sh
379	2023/1/30 23:02:36	211s	micr(	N/A	117.136.8.252 (Shanghai-Sha
380	2023/1/31 7:22:28	400s	micr(	N/A	218.18.128.68 (Guangdong - :
381	2023/1/31 19:38:46	410s	micr(	N/A	222.130.178.171 (Beijing-Beiji
382	2023/1/31 19:38:48	402s	micr(	N/A	58.33.136.53 (Shanghai-Shan
383	2023/1/31 23:54:11	214s	micr(	N/A	112.97.80.189 (Guangdong - :
384	2023/2/1 13:55:52	266s	micr(	N/A	180.102.176.154 (Jiangsu-Na
385	2023/2/1 15:44:05	14s	micr(	N/A	183.6.9.97 (Guangdong - Gua
386	2023/2/1 16:41:17	11s	micr(	N/A	182.32.138.181 (Shandong-D(
387	2023/2/6 13:10:26	138s	micr(	N/A	112.97.83.171 (Guangdong - :
388	2023/2/6 21:52:22	225s	micr(	N/A	113.87.218.246 (Guangdong -
389	2023/2/7 18:37:42	211s	micr(	N/A	117.136.40.183 (Guangdong -

1. Please select the number of years you have participated in	2. Age	3、Please select	4. Highest degree
1	-3	-3	-3
2	6	Shanghai-Pudon	4
2	2	Guangdong-Gua	5
1	-3	-3	-3
2	1	Beijing-Dongche	1
4	2	Shanghai-Songji	3
4	2	Shanghai-Qingpu	4
2	1	Shanghai-Huang	2
2	1	Beijing - Xicheng	5
4	2	Sichuan - Cheng	2
2	1	Beijing - Xicheng	5
2	2	Beijing-Dongche	4
2	4	Beijing - Xicheng	5
3	3	Beijing-Haidian D	5
3	1	Zhejiang-Hangzh	3
4	3	Shaanxi - Xi'an	4
1	-3	-3	-3
2	3	Sichuan - Cheng	2
1	-3	-3	-3
3	2	Beijing-Haidian D	2
4	6	Shanghai-Hongk	4
2	4	Shanghai-Pudon	4
4	6	Beijing-Haidian D	4
2	2	Shandong - Jinan	2
2	2	Guangdong-Gua	2
3	8	Guangdong-Gua	4
5	4	Beijing-Dongche	4
4	4	Xinjiang-Urumqi	3
2	1	Zhejiang-Hangzh	5
1	-3	-3	-3
2	1	Beijing-Fengtai D	5
4	4	Beijing - Chaoyan	5
2	1	Shanghai-Xuhui	2
1	-3	-3	-3
4	4	Fujian-Quanzhou	2
4	4	Jiangsu - Nanjing	4
4	5	Guangdong-She	4
1	-3	-3	-3
4	4	Zhejiang-Hangzh	5
2	3	Zhejiang-Hangzh	4
3	3	Beijing-Haidian D	5
5	5	Guangxi - Nannin	4
4	3	Beijing-Haidian D	4
3	3	Zhejiang-Hangzh	4
2	2	Zhejiang-Hangzh	4
2	3	Liaoning-Shenya	3
5	5	Shandong - Jinan	1
5	3	Sichuan - Cheng	2
1	-3	-3	-3
2	3	Guangdong-She	5
3	1	Beijing - Xicheng	1
2	1	Shaanxi - Xi'an	2
2	3	Zhejiang-Hangzh	3
2	1	Beijing-Haidian D	3
4	5	Liaoning-Shenya	5

2	4	Beijing-Fengtai D	4
2	2	Shanghai-Yangp	2
2	1	Beijing - Xicheng	5
4	2	Beijing-Fengtai D	4
2	2	Beijing - Chaoyar	4
2	3	Yunnan - Kunmir	3
2	2	Jilin-Changchun	5
2	3	Shanghai-Huang	3
4	3	Shanghai-Pudon	3
3	3	Hubei - Jingzhou	2
1	-3	-3	-3
2	1	Shanghai-Xuhui	2
2	1	Beijing-Fengtai D	4
2	2	Zhejiang-Hangzh	3
2	1	Guangdong-Gua	1
5	3	Jiangsu - Nanjing	5
3	1	Jiangsu - Nanjing	3
4	3	Beijing - Chaoyar	2
2	3	Hubei - Wuhan	4
3	4	Beijing - Chaoyar	3
1	-3	-3	-3
4	2	Shanghai - Jing'a	5
2	3	Fujian-Fuzhou	5
1	-3	-3	-3
2	2	Henan - Zhengzh	2
2	1	Sichuan - Cheng	2
1	-3	-3	-3
3	3	Shanghai-Pudon	2
2	2	Hunan - Changsl	5
2	1	Guangdong-Gua	2
1	-3	-3	-3
1	-3	-3	-3
1	-3	-3	-3
2	1	Shanghai-Xuhui	3
2	1	Ningxia-Yinchuar	5
1	-3	-3	-3
3	3	Shaanxi - Xi'an	2
1	-3	-3	-3
4	4	Shanghai - Jing'a	5
3	3	Beijing - Xicheng	4
1	-3	-3	-3
3	2	Shanghai - Jing'a	4
2	2	Beijing - Xicheng	2
2	2	Beijing-Haidian D	4
3	2	Shanghai-Yangp	5
2	1	Liaoning - Dalian	2
1	-3	-3	-3
4	3	Beijing-Changpir	4
4	2	Anhui - Bozhou	2
2	1	Yunnan - Kunmir	2
5	3	Fujian-Fuzhou	1
4	3	Guangdong-Gua	2
2	3	Guizhou - Guiyar	5
5	5	Shanghai-Minhar	3
1	-3	-3	-3
1	-3	-3	-3

2	3	Zhejiang-Huzhou	2
2	2	Shanghai-Pudon	3
2	3	Beijing - Xicheng	5
1	-3	-3	-3
2	1	Beijing-Dongche	2
2	2	Guangdong-She	2
3	2	Hubei - Wuhan	3
4	2	Shanghai-Xuhui	2
2	2	Tianjin - Jinnan D	5
2	1	Macau - Taipa Is	3
1	-3	-3	-3
5	3	Zhejiang-Hangzh	5
2	2	Shanghai-Yangp	2
2	2	Shanghai-Huang	4
2	4	Yunnan - Kunmir	2
1	-3	-3	-3
4	2	Anhui - Bozhou	2
2	3	Zhejiang-Wenzh	5
1	-3	-3	-3
2	1	Beijing-Dongche	5
2	1	Guangdong - Zh	2
4	2	Shanghai-Xuhui	5
2	2	Jiangsu-Suzhou	4
2	1	Tianjin-Nankai D	3
2	5	Beijing - Chaoyar	2
2	4	Shanghai-Huang	5
1	-3	-3	-3
2	2	Shandong - Jinar	2
4	4	Shanghai-Huang	4
3	1	Guangdong-Gua	2
5	3	Shandong - Liao	3
1	-3	-3	-3
1	-3	-3	-3
3	1	Shanghai-Pudon	4
3	4	Guangdong-Gua	4
5	5	Beijing - Xicheng	5
2	3	Beijing - Xicheng	4
2	1	Shanghai-Pudon	1
3	4	Shanghai-Pudon	3
2	3	Jiangxi - Ganzho	2
4	7	Beijing-Haidian D	5
5	4	Guangdong-Gua	4
2	3	Zhejiang-Wenzh	5
2	1	Yunnan - Kunmir	2
2	2	Guangdong-Gua	2
2	1	Shandong-Zibo	2
2	2	Shanghai-Pudon	3
1	-3	-3	-3
1	-3	-3	-3
1	-3	-3	-3
3	2	Beijing-Haidian D	4
1	-3	-3	-3
3	2	Shanghai - Jing'a	5
2	1	Jiangsu - Nanjing	2
3	2	Sichuan - Cheng	2
1	-3	-3	-3

2	2	Beijing - Xicheng	3
2	3	Shanghai-Pudon	1
5	6	Shanghai-Huang	4
2	2	Guangdong-Gua	3
2	2	Zhejiang-Hangzh	5
2	1	Beijing - Chaoyar	2
1	-3	-3	-3
1	-3	-3	-3
2	5	Shandong-Weifa	1
5	7	Beijing-Fengtai D	5
2	3	Chongqing-Shap	1
3	3	Shanghai-Pudon	4
2	3	Beijing - Chaoyar	4
1	-3	-3	-3
1	-3	-3	-3
2	2	Hubei - Enshi	2
2	2	Beijing-Shijingsh	5
2	3	Beijing-Fengtai D	5
4	2	Jiangxi - Nancha	4
4	6	Jilin-Changchun	5
3	5	Zhejiang-Wenzh	1
1	-3	-3	-3
3	6	Xinjiang-Urumqi	5
2	3	Shanghai-Xuhui	3
2	2	Shanghai-Chang	4
5	7	Shandong - Jinar	5
2	1	Shanghai-Pudon	3
3	3	Beijing-Changpir	5
1	-3	-3	-3
1	-3	-3	-3
2	3	Shandong - Liao	3
4	5	Guangdong-Gua	5
1	-3	-3	-3
4	1	Beijing-Daxing D	1
2	2	Beijing-Daxing D	3
2	2	Beijing-Daxing D	4
3	3	Beijing - Chaoyar	3
2	1	Shanxi-Taiyuan	1
1	-3	-3	-3
2	2	Zhejiang-Hangzh	5
2	1	Beijing-Haidian D	3
5	5	Shanghai-Yangp	5
3	1	Guangdong-Gua	4
2	2	Yunnan - Kunmir	2
3	2	Anhui - Hefei	3
1	-3	-3	-3
2	1	Guangdong-Gua	5
2	1	Guangdong-She	1
2	2	Guangdong-Gua	2
3	2	Fujian - Xiamen	4
2	3	Henan - Zhengzh	4
4	4	Xinjiang-Urumqi	5
2	1	Guangdong-Gua	5
2	2	Hunan - Yiyang	2
3	4	Guangxi - Nannir	4
3	4	Guangxi - Nannir	4

2	1	Beijing-Daxing D	2
2	2	Zhejiang-Hangzh	5
4	2	Zhejiang-Hangzh	3
5	4	Guangdong-Gua	3
4	3	Zhejiang-Hangzh	3
4	3	Anhui - Huainan	3
2	3	Jiangsu-Wuxi	5
2	2	Sichuan - Cheng	2
1	-3	-3	-3
5	7	Jiangsu-Wuxi	4
4	4	Anhui - Hefei	4
4	3	Beijing-Haidian D	3
3	3	Hainan - Sanya	4
4	2	Beijing - Chaoyai	2
5	6	Shanghai-Yangp	2
1	-3	-3	-3
1	-3	-3	-3
1	-3	-3	-3
4	3	Shanghai-Pudon	2
1	-3	-3	-3
2	3	Shanxi-Taiyuan	4
4	2	Beijing-Haidian D	3
3	3	Shanghai-Pudon	3
2	1	Shanghai-Pudon	3
4	3	Shanghai-Pudon	4
1	-3	-3	-3
5	3	Jiangsu - Nanjing	3
2	2	Beijing-Haidian D	2
2	3	Beijing - Chaoyai	2
2	1	Jiangsu-Suzhou	1
4	3	Beijing - Xicheng	2
2	5	Shanghai-Pudon	4
5	6	Beijing-Haidian D	1
1	-3	-3	-3
2	1	Shandong - Jinai	2
2	3	Shanghai-Pudon	4
5	4	Beijing-Haidian D	3
2	4	Shandong - Jinai	5
3	4	Beijing-Dongche	3
3	4	Shanghai-Putuo	3
2	1	Shanghai-Huang	2
2	2	Guangdong-She	2
2	1	Shanghai-Jinsha	2
1	-3	-3	-3
2	2	Beijing-Dongche	3
3	2	Jiangsu - Nanjing	5
2	2	Beijing-Changpir	4
4	6	Beijing - Chaoyai	4
5	8	Jiangsu - Nanjing	3
1	-3	-3	-3
2	2	Shanghai-Pudon	4
5	4	Shanghai-Pudon	2
2	1	Jiangsu - Nanjing	1
1	-3	-3	-3
5	3	Beijing - Chaoyai	3
1	-3	-3	-3

3	3	Sichuan - Cheng	5
4	3	Beijing - Chaoyan	4
4	2	Beijing-Changpin	3
2	4	Shaanxi - Xi'an	2
3	2	Beijing - Chaoyan	4
4	5	Jiangsu-Suzhou	5
1	-3	-3	-3
1	-3	-3	-3
1	-3	-3	-3
4	3	Beijing - Chaoyan	4
2	4	Shanghai-Pudon	4
2	3	Beijing-Daxing D	1
2	6	Guangdong-Zhan	4
3	3	Beijing-Dongche	2
1	-3	-3	-3
2	1	Sichuan - Cheng	5
5	5	Ningxia-Yinchuan	5
3	2	Guangdong-Gua	5
1	-3	-3	-3
3	1	Shaanxi - Xi'an	2
4	2	Beijing - Xicheng	5
5	3	Chongqing - Jian	3
5	6	Beijing-Haidian D	5
2	1	Shanghai-Jinsha	2
2	1	Shanghai-Pudon	2
4	2	Shanghai-Yangp	5
5	6	Guangdong-Zhan	1
5	4	Shanghai-Yangp	4
2	3	Zhejiang-Ningbo	4
2	1	Anhui-Fuyang	2
3	2	Guangdong-Zhan	4
1	-3	-3	-3
2	2	Beijing - Chaoyan	4
5	6	Beijing - Chaoyan	3
4	2	Shanghai - Jing'a	5
5	3	Guangdong-Gua	5
2	2	Beijing-Dongche	4
2	6	Shanghai-Pudon	4
3	3	Sichuan - Mianya	3
3	1	Shanghai - Jing'a	3
2	4	Beijing-Haidian D	4
2	3	Fujian-Fuzhou	2
4	1	Beijing-Haidian D	3
4	5	Shandong - Zaoz	5
4	5	Beijing-Haidian D	4
2	2	Beijing - Xicheng	4
5	5	Jiangsu - Nanjing	5
4	2	Beijing - Xicheng	3
2	2	Shanghai-Putuo	5
2	4	Beijing-Haidian D	4
1	-3	-3	-3
2	1	Hubei - Wuhan	2
3	3	Shanghai-Pudon	4
1	-3	-3	-3
2	2	Shanghai-Pudon	4
1	-3	-3	-3

2	3	Beijing-Changpir	4
1	-3	-3	-3
2	2	Beijing-Dongche	5
5	2	Zhejiang-Hangzh	5
2	3	Henan - Zhengzh	2
2	3	Shaanxi - Xi'an	5
1	-3	-3	-3
3	2	Shaanxi - Xi'an	5
2	2	Beijing - Chaoyar	5
1	-3	-3	-3
4	3	Shandong - Yant	5
4	4	Zhejiang-Hangzh	5
2	1	Chongqing-Shap	3
1	-3	-3	-3
3	4	Yunnan - Kunmir	5
4	2	Hunan - Changsl	5
2	1	Liaoning - Dalian	2
4	2	Jiangsu - Nanjing	2
5	3	Jiangsu-Suzhou	5
4	2	Beijing-Daxing D	2
3	6	Zhejiang-Wenzh	5
1	-3	-3	-3
1	-3	-3	-3
1	-3	-3	-3
1	-3	-3	-3
1	-3	-3	-3
2	3	Chongqing-Shap	3
2	1	Guangdong-Gua	5
1	-3	-3	-3
4	4	Hubei - Wuhan	5
3	6	Shandong - Jinar	3
1	-3	-3	-3
2	4	Jiangsu-Taizhou	2
3	3	Guangdong-She	3
3	3	Beijing - Xicheng	3
3	4	Beijing-Fengtai D	4
5	4	Anhui - Hefei	5
3	3	Hebei - Handan	4
2	1	Shandong - Jinar	1
5	3	Beijing-Haidian D	5
2	5	Beijing-Dongche	5
3	2	Sichuan - Cheng	2
2	2	Shanghai-Xuhui	2
4	3	Shanghai-Minhar	4
3	3	Guangdong-Gua	4
3	3	Beijing-Haidian D	2
2	2	Shanghai-Xuhui	2
2	2	Beijing-Dongche	4
3	1	Jiangxi - Nancha	5
1	-3	-3	-3
1	-3	-3	-3
2	1	Guangdong-She	1
2	2	Guangdong-She	2
2	5	Guangdong-She	2

5. Main job title (please select ar	6. Nature of hospital (please	7. Universities and Colleges (
-3	-3	-3
3	-3	-3
1	1	-3
-3	-3	-3
1	1	-3
5	-3	-3
3	-3	-3
3	-3	-3
1	1	-3
1	1	-3
1	1	-3
4	-3	-3
1	1	-3
1	1	-3
1	1	-3
1	1	-3
-3	-3	-3
1	1	-3
-3	-3	-3
1	1	-3
1	1	-3
1	1	-3
1	1	-3
1	1	-3
5	-3	-3
2	-3	1
1	1	-3
1	1	-3
1	1	-3
-3	-3	-3
1	1	-3
1	2	-3
1	1	-3
-3	-3	-3
1	1	-3
1	1	-3
1	1	-3
-3	-3	-3
1	1	-3
3	-3	-3
1	1	-3
1	1	-3
1	1	-3
1	1	-3
1	1	-3
1	1	-3
5	-3	-3
3	-3	-3
-3	-3	-3
3	-3	-3
5	-3	-3
1	1	-3
1	1	-3
2	-3	2
1	1	-3

1	1	-3
5	-3	-3
1	1	-3
1	1	-3
1	1	-3
1	1	-3
1	1	-3
1	1	-3
3	-3	-3
1	1	-3
-3	-3	-3
2	-3	1
3	-3	-3
1	1	-3
5	-3	-3
1	1	-3
1	1	-3
3	-3	-3
3	-3	-3
1	1	-3
-3	-3	-3
1	1	-3
1	1	-3
-3	-3	-3
1	1	-3
3	-3	-3
-3	-3	-3
1	1	-3
1	1	-3
1	1	-3
-3	-3	-3
-3	-3	-3
-3	-3	-3
1	1	-3
2	-3	1
-3	-3	-3
1	1	-3
-3	-3	-3
1	1	-3
3	-3	-3
-3	-3	-3
3	-3	-3
1	1	-3
1	1	-3
1	1	-3
5	-3	-3
-3	-3	-3
3	-3	-3
1	1	-3
5	-3	-3
1	6	-3
1	1	-3
1	1	-3
5	-3	-3
-3	-3	-3
-3	-3	-3

1	1	-3
1	1	-3
1	1	-3
-3	-3	-3
1	1	-3
1	1	-3
3	-3	-3
1	1	-3
1	1	-3
2	-3	1
-3	-3	-3
1	1	-3
1	1	-3
1	2	-3
1	1	-3
-3	-3	-3
1	1	-3
1	1	-3
-3	-3	-3
1	5	-3
1	1	-3
3	-3	-3
3	-3	-3
5	-3	-3
3	-3	-3
1	1	-3
-3	-3	-3
3	-3	-3
1	1	-3
1	1	-3
1	1	-3
-3	-3	-3
-3	-3	-3
1	1	-3
1	1	-3
1	1	-3
1	1	-3
3	-3	-3
2	-3	1
1	1	-3
2	-3	1
1	1	-3
1	1	-3
1	1	-3
1	1	-3
1	1	-3
5	-3	-3
-3	-3	-3
-3	-3	-3
-3	-3	-3
1	1	-3
-3	-3	-3
1	2	-3
5	-3	-3
1	1	-3
-3	-3	-3

5	-3	-3
3	-3	-3
1	1	-3
1	1	-3
1	1	-3
1	1	-3
-3	-3	-3
-3	-3	-3
1	1	-3
3	-3	-3
3	-3	-3
5	-3	-3
3	-3	-3
-3	-3	-3
-3	-3	-3
1	1	-3
1	1	-3
1	1	-3
1	1	-3
1	1	-3
1	1	-3
-3	-3	-3
1	1	-3
1	1	-3
1	1	-3
1	1	-3
3	-3	-3
1	2	-3
-3	-3	-3
-3	-3	-3
1	1	-3
1	1	-3
-3	-3	-3
5	-3	-3
3	-3	-3
3	-3	-3
2	-3	2
1	1	-3
-3	-3	-3
1	1	-3
1	1	-3
1	1	-3
5	-3	-3
1	1	-3
1	1	-3
-3	-3	-3
1	1	-3
1	1	-3
1	1	-3
1	1	-3
1	1	-3
1	1	-3
1	1	-3
1	6	-3
1	1	-3
1	1	-3

3	-3	-3
1	1	-3
1	1	-3
1	1	-3
1	1	-3
1	1	-3
1	1	-3
3	-3	-3
-3	-3	-3
2	-3	1
3	-3	-3
1	1	-3
3	-3	-3
3	-3	-3
3	-3	-3
-3	-3	-3
-3	-3	-3
-3	-3	-3
3	-3	-3
-3	-3	-3
1	1	-3
3	-3	-3
3	-3	-3
3	-3	-3
3	-3	-3
-3	-3	-3
2	-3	2
1	1	-3
3	-3	-3
3	-3	-3
3	-3	-3
3	-3	-3
3	-3	-3
-3	-3	-3
1	1	-3
3	-3	-3
3	-3	-3
1	1	-3
3	-3	-3
3	-3	-3
5	-3	-3
5	-3	-3
1	1	-3
-3	-3	-3
3	-3	-3
3	-3	-3
3	-3	-3
3	-3	-3
1	1	-3
-3	-3	-3
5	-3	-3
3	-3	-3
3	-3	-3
-3	-3	-3
3	-3	-3
-3	-3	-3

3	-3	-3
3	-3	-3
3	-3	-3
1	1	-3
3	-3	-3
1	6	-3
-3	-3	-3
-3	-3	-3
-3	-3	-3
3	-3	-3
3	-3	-3
1	5	-3
1	1	-3
3	-3	-3
-3	-3	-3
5	-3	-3
1	1	-3
1	1	-3
-3	-3	-3
1	1	-3
1	1	-3
3	-3	-3
1	1	-3
1	1	-3
1	1	-3
1	1	-3
1	1	-3
1	2	-3
1	1	-3
1	1	-3
1	1	-3
-3	-3	-3
2	-3	2
1	1	-3
1	1	-3
1	1	-3
3	-3	-3
1	1	-3
1	1	-3
1	1	-3
3	-3	-3
1	1	-3
2	-3	1
1	1	-3
2	-3	2
1	1	-3
1	1	-3
2	-3	2
1	1	-3
1	1	-3
-3	-3	-3
1	1	-3
1	1	-3
-3	-3	-3
3	-3	-3
-3	-3	-3

1	1	-3
-3	-3	-3
1	1	-3
1	1	-3
1	1	-3
1	1	-3
-3	-3	-3
1	1	-3
1	1	-3
-3	-3	-3
1	1	-3
1	1	-3
1	1	-3
-3	-3	-3
1	1	-3
1	1	-3
1	1	-3
5	-3	-3
1	1	-3
3	-3	-3
1	1	-3
-3	-3	-3
-3	-3	-3
-3	-3	-3
-3	-3	-3
-3	-3	-3
1	1	-3
1	1	-3
-3	-3	-3
1	1	-3
1	1	-3
-3	-3	-3
1	1	-3
1	1	-3
1	1	-3
1	1	-3
1	1	-3
1	1	-3
3	-3	-3
1	1	-3
1	1	-3
1	1	-3
1	2	-3
1	1	-3
3	-3	-3
1	1	-3
1	1	-3
2	-3	1
1	1	-3
-3	-3	-3
-3	-3	-3
1	1	-3
1	1	-3
1	1	-3

8. Nature of the company (pl	9. Other titles (if there is a second unit of em
-3	-3
3	1
-3	1
-3	-3
-3	1
-3	6
4	1
3	1
-3	1
-3	1
-3	1
-3	6
-3	1
-3	1
-3	1
-3	3
-3	-3
-3	1
-3	-3
-3	1
-3	1
-3	1
-3	2
-3	1
-3	1
-3	1
-3	1
-3	1
-3	1
-3	-3
-3	1
-3	1
-3	1
-3	-3
-3	3
-3	3
-3	1
-3	-3
-3	1
3	1
-3	1
-3	1
-3	1
-3	1
-3	1
-3	1
-3	1
4	1
-3	-3
3	1
-3	1
-3	1
-3	1
-3	1
-3	3

-3	1
-3	1
-3	1
-3	1
-3	1
-3	1
-3	1
-3	1
1	1
-3	1
-3	-3
-3	1
1	2
-3	1
-3	1
-3	3
-3	1
3	1
2	1
-3	1
-3	-3
-3	1
-3	1
-3	-3
-3	1
4	1
-3	-3
-3	1
-3	1
-3	1
-3	-3
-3	-3
-3	-3
-3	1
-3	1
-3	-3
-3	1
-3	-3
-3	1
1	1
-3	-3
4	1
-3	1
-3	1
-3	1
-3	1
-3	-3
3	1
-3	1
-3	1
-3	1
-3	1
-3	1
-3	1
-3	-3
-3	-3

-3	1
-3	1
-3	2
-3	-3
-3	6
-3	1
4	1
-3	1
-3	1
-3	1
-3	-3
-3	1
-3	1
-3	1
-3	1
-3	-3
-3	1
-3	1
-3	-3
-3	1
-3	1
3	1
3	1
-3	1
2	1
-3	1
-3	-3
1	1
-3	1
-3	1
-3	1
-3	-3
-3	-3
-3	1
-3	2
-3	1
-3	1
4	1
-3	3
-3	1
-3	1
-3	3
-3	1
-3	1
-3	1
-3	1
-3	1
-3	-3
-3	-3
-3	-3
-3	1
-3	-3
-3	1
-3	1
-3	1
-3	-3

-3	1
3	1
-3	1
-3	1
-3	3
-3	1
-3	-3
-3	-3
-3	3
4	3
3	1
-3	1
4	1
-3	-3
-3	-3
-3	1
-3	3
-3	1
-3	1
-3	1
-3	2
-3	-3
-3	3
-3	1
-3	1
-3	2
1	1
-3	1
-3	-3
-3	-3
-3	1
-3	1
-3	-3
-3	1
3	1
2	1
-3	1
-3	1
-3	-3
-3	1
-3	1
-3	1
-3	1
-3	1
-3	1
-3	-3
-3	1
-3	1
-3	1
-3	3
-3	4
-3	1
-3	1
-3	1
-3	1
-3	1

2	1
-3	1
-3	2
-3	1
-3	1
-3	1
-3	1
2	1
-3	-3
-3	1
3	1
-3	1
2	1
1	4
3	1
-3	-3
-3	-3
-3	-3
3	1
-3	-3
-3	1
2	1
2	1
4	1
3	1
-3	-3
-3	1
-3	1
4	1
2	1
2	1
3	1
3	1
-3	-3
-3	1
4	1
4	1
-3	1
4	1
3	1
-3	1
-3	1
-3	1
-3	-3
1	1
2	1
3	1
2	3
-3	2
-3	-3
-3	1
2	1
4	1
-3	-3
3	1
-3	-3

2	4
1	1
3	1
-3	1
2	1
-3	2
-3	-3
-3	-3
-3	-3
4	1
3	4
-3	1
-3	2
3	1
-3	-3
-3	1
-3	1
-3	1
-3	-3
-3	1
-3	1
4	1
-3	1
-3	1
-3	6
-3	1
-3	3
-3	1
-3	1
-3	1
-3	1
-3	-3
-3	1
-3	3
-3	1
-3	1
3	1
-3	1
-3	1
-3	1
3	2
-3	1
-3	1
-3	1
-3	1
-3	1
-3	1
-3	1
-3	1
-3	1
-3	-3
-3	1
-3	1
-3	-3
2	1
-3	-3

-3	1
-3	-3
-3	1
-3	1
-3	1
-3	1
-3	-3
-3	1
-3	1
-3	-3
-3	1
-3	3
-3	1
-3	-3
-3	1
-3	1
-3	1
-3	1
-3	2
4	1
-3	1
-3	-3
-3	-3
-3	-3
-3	-3
-3	-3
-3	2
-3	1
-3	-3
-3	1
-3	1
-3	-3
-3	1
-3	1
-3	1
-3	1
-3	3
-3	1
2	1
-3	1
-3	1
-3	1
-3	1
-3	1
4	6
-3	1
-3	1
-3	1
-3	1
-3	-3
-3	-3
-3	1
-3	1
-3	1

10. Whether to participate in clinical research a

-3
2
2
-3
2
2
2
2
2
2
2
2
2
2
2
1
-3
2
-3
2
1
2
2
2
2
1
1
2
2
-3
2
2
2
-3
2
1
1
-3
2
2
2
1
2
1
2
2
2
2
-3
2
2
1
2
2
2

2
2
2
2
2
2
2
2
2
2
-3
2
1
2
1
1
2
2
2
2
-3
2
2
-3
1
2
-3
1
2
2
-3
-3
-3
2
2
-3
1
-3
1
2
-3
2
2
2
1
2
-3
2
2
2
2
2
1
2
-3
-3

2
2
2
-3
2
2
2
1
2
2
-3
1
2
1
1
-3
1
1
-3
2
2
2
2
2
2
2
-3
2
1
2
2
-3
-3
2
2
1
2
2
1
2
2
1
1
2
2
2
2
-3
-3
-3
2
-3
2
2
1
-3

2
2
1
2
2
2
-3
-3
2
1
2
2
2
-3
-3
2
2
2
1
1
2
-3
1
1
1
1
2
2
-3
-3
2
2
-3
2
2
2
2
2
-3
2
2
1
2
2
2
-3
2
2
2
1
2
1
2
2
2
1

2
2
1
1
2
2
2
2
-3
1
1
2
2
2
2
-3
-3
-3
2
-3
2
2
2
2
2
-3
2
2
2
2
2
2
2
-3
1
2
2
2
1
2
2
2
2
-3
2
2
2
2
1
-3
2
2
2
-3
2
-3

1
2
2
2
2
1
-3
-3
-3
2
2
2
1
2
-3
2
2
2
-3
1
2
2
2
2
2
2
1
2
2
2
2
-3
2
1
2
2
2
2
1
2
2
2
2
2
1
2
1
2
2
2
-3
1
2
-3
2
-3

2
-3
1
1
2
2
-3
2
2
-3
2
2
2
-3
1
2
1
2
2
2
2
-3
-3
-3
-3
-3
2
2
-3
2
1
-3
2
2
2
2
1
2
2
2
1
1
1
1
2
2
2
2
2
-3
-3
2
2
2

11. What types of clinical studies have you been involved in, and i	11. (Single-center rand	11. (Prospective co
-3	-3	-3
1	0	0
0	1	0
-3	-3	-3
1	0	1
1	1	0
0	1	1
1	0	0
0	1	1
1	1	0
0	0	0
1	0	1
1	0	1
1	1	1
1	1	1
1	1	1
-3	-3	-3
0	0	0
-3	-3	-3
1	0	0
1	0	0
0	0	0
0	0	0
0	1	0
0	1	0
0	1	1
1	0	0
1	0	0
1	0	1
-3	-3	-3
0	1	1
0	0	0
0	0	0
-3	-3	-3
0	0	1
0	0	1
1	1	1
-3	-3	-3
1	0	1
1	0	0
0	1	1
1	1	1
1	1	1
1	1	0
0	0	0
0	1	0
0	0	0
1	1	1
-3	-3	-3
0	0	0
1	0	1
0	1	0
1	0	0
1	1	1
1	1	1

0	0	0
0	1	0
0	1	1
1	0	1
1	0	0
0	0	0
0	1	1
0	1	1
1	0	0
0	0	0
-3	-3	-3
0	1	1
1	1	1
0	0	1
0	1	1
1	1	1
1	0	0
1	1	0
1	1	0
1	0	0
-3	-3	-3
0	1	1
1	0	0
-3	-3	-3
0	0	1
1	1	0
-3	-3	-3
0	0	1
0	0	0
0	0	0
-3	-3	-3
-3	-3	-3
-3	-3	-3
1	0	1
0	0	0
-3	-3	-3
0	1	0
-3	-3	-3
1	0	1
1	1	0
-3	-3	-3
1	1	1
1	0	0
0	1	1
1	1	1
0	1	0
-3	-3	-3
1	1	1
0	0	0
0	0	0
0	0	0
1	1	0
1	0	1
0	1	1
-3	-3	-3
-3	-3	-3

0	0	0
1	0	1
1	0	0
-3	-3	-3
0	0	0
0	0	0
1	0	0
1	1	0
0	0	0
1	0	0
-3	-3	-3
1	1	1
0	0	0
0	0	0
0	1	0
-3	-3	-3
1	0	1
1	0	1
-3	-3	-3
1	0	0
0	0	0
1	1	1
0	0	1
0	0	0
1	0	0
1	0	0
-3	-3	-3
0	1	0
1	1	1
0	0	0
0	0	0
-3	-3	-3
-3	-3	-3
0	0	1
0	0	0
1	1	0
0	0	0
1	1	0
1	0	0
1	0	0
1	0	0
1	1	1
1	1	1
0	0	0
0	0	0
0	0	0
1	0	1
-3	-3	-3
-3	-3	-3
-3	-3	-3
1	0	1
-3	-3	-3
0	1	0
0	0	1
1	1	1
-3	-3	-3

1	0	0
1	0	0
0	0	1
1	0	0
1	0	1
1	0	1
-3	-3	-3
-3	-3	-3
0	0	0
1	1	0
0	0	0
1	1	0
0	1	1
-3	-3	-3
-3	-3	-3
1	0	0
0	0	1
0	0	0
0	0	1
1	0	1
1	0	1
-3	-3	-3
0	0	1
0	1	1
0	1	0
1	0	0
1	1	0
1	0	0
-3	-3	-3
-3	-3	-3
0	1	0
1	0	0
-3	-3	-3
1	1	0
1	0	0
1	0	1
1	0	1
0	0	1
-3	-3	-3
1	0	0
0	0	1
1	1	1
1	1	1
0	0	0
0	0	0
-3	-3	-3
1	0	1
0	0	0
0	1	0
1	0	0
1	0	0
1	0	0
0	1	0
0	0	0
0	0	0
0	0	0

1	1	1
1	1	0
1	1	1
0	1	1
1	1	1
0	0	0
1	0	0
1	0	1
-3	-3	-3
1	1	1
1	1	1
1	1	1
1	0	0
1	1	1
1	0	0
-3	-3	-3
-3	-3	-3
-3	-3	-3
1	0	0
-3	-3	-3
0	0	1
1	1	0
1	0	0
1	0	1
0	0	1
-3	-3	-3
1	1	0
0	0	0
0	0	0
1	0	0
0	0	1
0	1	1
1	1	1
-3	-3	-3
0	1	1
1	1	0
1	1	1
1	0	0
1	0	0
1	0	0
1	0	0
1	0	0
1	0	0
-3	-3	-3
1	0	1
1	1	1
1	1	0
1	1	1
0	1	0
-3	-3	-3
1	0	0
1	1	1
1	0	1
-3	-3	-3
1	1	1
-3	-3	-3

1	1	1
1	0	1
1	0	1
1	0	1
1	0	1
1	0	0
-3	-3	-3
-3	-3	-3
-3	-3	-3
0	0	0
1	1	1
0	1	0
1	0	0
1	1	1
-3	-3	-3
0	0	0
1	1	0
1	1	1
-3	-3	-3
0	1	0
0	0	1
1	1	1
1	1	0
1	0	1
0	0	0
0	1	0
1	1	1
0	0	1
0	0	0
0	0	0
0	0	1
-3	-3	-3
0	0	1
1	1	1
1	1	1
1	1	1
0	0	0
0	0	1
0	1	1
0	0	1
1	1	0
1	0	0
1	1	1
0	1	0
0	1	0
1	1	0
1	1	0
1	0	1
1	0	0
1	0	0
-3	-3	-3
1	0	0
1	1	1
-3	-3	-3
1	0	0
-3	-3	-3

0	1	1
-3	-3	-3
0	1	0
1	1	1
1	0	1
1	0	1
-3	-3	-3
1	1	1
1	0	0
-3	-3	-3
1	0	1
1	0	0
1	0	1
-3	-3	-3
1	0	0
1	0	0
0	1	0
1	1	1
1	0	1
1	1	1
0	0	1
-3	-3	-3
-3	-3	-3
-3	-3	-3
-3	-3	-3
-3	-3	-3
1	1	0
1	0	1
-3	-3	-3
1	0	0
1	0	0
-3	-3	-3
0	1	1
1	0	0
0	0	0
0	1	1
1	1	1
0	0	0
1	0	0
1	1	1
0	1	0
0	1	1
0	1	0
1	0	1
1	1	0
1	0	0
1	0	0
0	0	0
0	1	1
-3	-3	-3
-3	-3	-3
0	0	1
0	0	0
0	0	0

11. (Case-control	11. (Cross-secti	11. (Case rep	11. (Other	12. (Multicenter randon	12. (Single-center rand
-3	-3	-3	-3	-2	-2
0	0	0	0	1	-2
0	0	0	0	-2	1
-3	-3	-3	-3	-2	-2
1	1	1	0	-2	-2
0	0	0	1	1	2
1	1	0	0	-2	3
0	0	0	0	1	-2
1	1	0	0	-2	1
0	1	1	0	1	2
0	1	1	1	-2	-2
0	1	1	0	4	3
0	0	0	0	1	-2
1	1	1	0	1	3
1	1	1	0	1	2
0	0	1	0	1	-2
-3	-3	-3	-3	-2	-2
1	0	1	0	-2	-2
-3	-3	-3	-3	-2	-2
0	0	0	0	1	-2
1	0	0	0	1	-2
0	1	1	0	-2	-2
1	1	0	0	-2	-2
0	0	0	0	-2	1
0	0	1	0	-2	1
1	1	0	0	-2	4
0	0	0	0	1	-2
0	0	1	0	2	-2
0	0	1	0	1	-2
-3	-3	-3	-3	-2	-2
1	1	0	0	-2	4
1	0	1	0	-2	-2
1	1	1	0	-2	-2
-3	-3	-3	-3	-2	-2
0	0	1	0	2	-2
1	1	1	0	-2	-2
0	0	0	0	2	1
-3	-3	-3	-3	-2	-2
1	0	1	0	1	6
0	0	0	0	1	-2
1	1	1	0	-2	4
0	0	1	0	1	3
1	1	1	0	-2	1
0	0	0	0	1	2
1	1	0	0	-2	-2
0	1	0	0	-2	1
0	0	0	1	-2	-2
0	0	0	0	1	3
-3	-3	-3	-3	-2	-2
0	0	0	1	4	3
0	0	0	0	2	-2
0	1	0	0	-2	2
0	0	0	0	1	2
1	1	1	0	-2	-2
1	1	0	0	5	1

1	1	0	0	-2	-2
1	1	0	0	-2	2
1	1	1	0	-2	1
0	0	0	0	1	-2
0	0	0	0	1	-2
1	0	0	0	-2	-2
1	1	1	0	-2	4
1	1	0	0	4	2
0	0	0	0	1	-2
1	0	0	0	-2	-2
-3	-3	-3	-3	-2	-2
0	0	0	0	-2	2
1	0	0	0	-2	1
0	0	0	0	-2	-2
0	0	1	0	-2	1
1	0	1	0	1	2
1	0	0	0	1	-2
1	0	1	1	1	-2
0	0	0	0	-2	1
0	0	0	0	1	-2
-3	-3	-3	-3	-2	-2
1	1	1	0	-2	4
1	0	1	0	4	-2
-3	-3	-3	-3	-2	-2
0	0	0	0	-2	-2
0	0	0	0	1	2
-3	-3	-3	-3	-2	-2
0	0	0	0	-2	-2
1	0	1	0	-2	-2
1	1	1	0	-2	4
-3	-3	-3	-3	-2	-2
-3	-3	-3	-3	-2	-2
-3	-3	-3	-3	-2	-2
1	0	0	0	1	-2
1	0	0	0	-2	-2
-3	-3	-3	-3	-2	-2
0	0	0	0	-2	1
-3	-3	-3	-3	-2	-2
0	1	0	0	6	5
1	0	0	0	3	1
-3	-3	-3	-3	-2	-2
0	0	0	0	1	2
1	0	0	0	1	2
0	0	0	0	-2	2
1	0	1	0	2	1
0	0	0	0	-2	1
-3	-3	-3	-3	-2	-2
1	1	0	0	4	3
1	0	1	0	6	2
1	0	0	0	-2	-2
1	1	1	1	-2	-2
1	0	0	0	3	1
0	0	0	0	1	-2
1	1	0	0	-2	4
-3	-3	-3	-3	-2	-2
-3	-3	-3	-3	-2	-2

1	0	0	0	-2	-2
0	0	0	0	1	-2
0	1	0	0	2	-2
-3	-3	-3	-3	-2	-2
1	1	1	0	-2	-2
0	0	1	0	-2	-2
0	0	0	0	1	-2
1	1	1	0	6	1
1	0	0	0	-2	-2
0	0	0	0	1	-2
-3	-3	-3	-3	-2	-2
1	1	1	0	4	3
0	0	1	0	-2	-2
1	1	0	0	-2	-2
0	0	0	0	-2	-2
-3	-3	-3	-3	-2	-2
0	1	0	0	2	-2
0	0	0	0	1	-2
-3	-3	-3	-3	-2	-2
0	0	0	0	-2	-2
1	1	1	0	-2	-2
1	1	1	1	1	3
1	0	0	0	-2	-2
1	0	0	0	-2	-2
0	0	0	0	1	2
0	0	0	0	4	-2
-3	-3	-3	-3	-2	-2
0	0	0	0	-2	1
1	0	0	0	-2	1
0	0	1	0	-2	-2
1	0	1	0	-2	-2
-3	-3	-3	-3	-2	-2
-3	-3	-3	-3	-2	-2
0	0	0	0	-2	-2
0	1	1	0	-2	-2
0	1	1	0	2	1
1	0	0	0	-2	-2
0	0	1	0	1	2
0	1	0	0	-2	1
1	1	1	0	1	2
0	0	0	0	1	3
0	1	0	0	1	2
0	0	0	0	3	2
1	0	1	0	6	5
1	0	1	0	-2	-2
0	0	0	1	-2	-2
1	0	1	0	1	-2
-3	-3	-3	-3	-2	-2
-3	-3	-3	-3	-2	-2
-3	-3	-3	-3	-2	-2
0	0	0	1	1	-2
-3	-3	-3	-3	-2	-2
0	0	0	0	-2	1
0	0	0	0	-2	-2
0	1	1	0	1	2
-3	-3	-3	-3	-2	-2

0	0	0	0	1	-2
0	0	0	0	1	-2
0	1	0	0	-2	-2
1	1	1	0	4	5
0	1	0	0	1	-2
0	0	0	0	1	-2
-3	-3	-3	-3	-2	-2
-3	-3	-3	-3	-2	-2
0	0	1	0	-2	-2
0	0	0	0	1	2
1	0	1	0	-2	-2
1	0	0	0	2	1
0	0	0	0	-2	1
-3	-3	-3	-3	-2	-2
-3	-3	-3	-3	-2	-2
0	0	0	0	1	-2
1	0	0	0	-2	-2
1	1	1	0	-2	-2
1	1	1	0	-2	-2
1	1	0	0	3	5
0	0	0	0	1	-2
-3	-3	-3	-3	-2	-2
0	0	0	0	-2	-2
0	0	0	0	-2	2
0	0	0	0	1	2
0	0	0	0	-2	1
0	0	0	0	1	-2
1	0	0	0	-2	-2
-3	-3	-3	-3	-2	-2
-3	-3	-3	-3	-2	-2
1	1	0	0	-2	-2
0	0	0	0	1	-2
-3	-3	-3	-3	-2	-2
0	0	0	0	1	2
0	0	0	0	1	-2
0	0	0	0	2	-2
0	1	0	0	1	-2
0	1	0	0	-2	-2
-3	-3	-3	-3	-2	-2
1	0	0	0	2	-2
1	0	1	0	-2	-2
1	1	1	0	1	3
0	1	0	0	1	4
0	0	0	1	-2	-2
1	1	0	0	-2	-2
-3	-3	-3	-3	-2	-2
0	0	0	0	-2	-2
0	1	1	0	-2	-2
0	0	0	0	-2	1
1	0	0	0	1	-2
0	0	0	0	1	-2
0	0	0	0	1	-2
0	0	0	0	-2	1
0	1	0	0	-2	-2
1	1	1	0	-2	-2
1	1	1	0	-2	-2

0	0	0	0	1	-2
0	0	0	0	1	2
1	1	0	0	4	3
0	1	0	0	-2	3
1	1	0	0	2	3
0	0	1	0	-2	-2
0	0	0	0	1	-2
0	0	0	1	1	-2
-3	-3	-3	-3	-2	-2
1	0	0	0	1	3
0	0	0	0	1	3
0	0	0	0	1	-2
0	0	0	0	1	-2
1	0	1	0	1	2
0	0	0	0	1	-2
-3	-3	-3	-3	-2	-2
-3	-3	-3	-3	-2	-2
-3	-3	-3	-3	-2	-2
0	0	0	0	1	-2
-3	-3	-3	-3	-2	-2
1	1	0	0	-2	-2
0	0	0	0	1	3
0	0	0	1	1	-2
0	0	0	0	1	-2
0	0	0	1	-2	-2
-3	-3	-3	-3	-2	-2
0	0	0	0	1	2
1	1	0	0	-2	1
0	0	1	0	-2	-2
0	0	0	0	1	-2
0	0	0	0	-2	-2
0	0	0	0	-2	1
1	1	0	0	1	3
-3	-3	-3	-3	-2	-2
0	1	0	0	-2	3
0	0	0	0	1	2
1	1	0	0	1	-2
0	0	0	0	1	-2
0	0	0	0	1	-2
0	1	0	0	1	-2
0	0	0	1	1	-2
0	0	0	0	1	-2
0	1	1	0	3	-2
-3	-3	-3	-3	-2	-2
0	0	0	0	-2	2
0	0	0	0	1	2
0	0	0	0	1	2
1	0	0	0	1	2
0	0	0	0	-2	1
-3	-3	-3	-3	-2	-2
0	0	0	0	1	-2
1	0	0	0	1	2
0	0	0	0	1	-2
-3	-3	-3	-3	-2	-2
1	1	1	0	1	-2
-3	-3	-3	-3	-2	-2

0	0	0	0	1	-2
0	0	0	0	1	-2
0	0	0	0	-2	-2
0	0	0	0	1	-2
0	0	0	0	2	-2
0	0	0	1	1	-2
-3	-3	-3	-3	-2	-2
-3	-3	-3	-3	-2	-2
-3	-3	-3	-3	-2	-2
0	0	0	1	-2	-2
1	0	0	0	3	2
0	0	0	0	-2	1
0	0	0	0	1	-2
1	1	0	0	1	2
-3	-3	-3	-3	-2	-2
0	1	1	0	-2	-2
1	0	1	0	1	2
0	0	0	0	1	-2
-3	-3	-3	-3	-2	-2
1	1	0	0	-2	2
0	0	0	0	-2	-2
1	1	1	0	1	2
0	1	0	0	1	2
0	1	1	0	3	-2
1	0	1	0	-2	-2
0	0	0	0	-2	1
1	1	1	0	1	2
0	1	0	0	7	4
1	1	0	0	3	2
1	0	0	0	-2	-2
0	0	1	0	1	-2
-3	-3	-3	-3	-2	-2
1	1	0	0	-2	-2
1	1	1	0	1	2
0	1	0	0	4	3
1	1	1	0	1	-2
1	0	0	1	-2	-2
0	0	0	0	-2	-2
0	0	0	0	6	1
0	0	0	0	-2	-2
1	0	0	0	2	1
0	0	1	0	1	-2
1	1	1	0	1	2
1	0	1	0	-2	2
1	0	1	0	-2	1
0	1	0	0	1	-2
0	0	1	0	1	2
1	1	0	0	2	-2
1	1	1	0	4	-2
0	1	0	0	2	-2
-3	-3	-3	-3	-2	-2
0	0	0	0	1	-2
1	1	1	0	6	1
-3	-3	-3	-3	-2	-2
0	0	1	0	1	-2
-3	-3	-3	-3	-2	-2

0	0	0	0	4	3
-3	-3	-3	-3	-2	-2
0	0	0	0	6	4
0	1	1	0	4	1
1	0	0	0	2	-2
1	0	0	0	1	2
-3	-3	-3	-3	-2	-2
0	0	0	1	3	1
1	1	0	0	2	-2
-3	-3	-3	-3	-2	-2
0	0	1	0	2	-2
0	0	0	0	2	1
1	1	0	0	2	-2
-3	-3	-3	-3	-2	-2
1	0	0	0	-2	-2
0	0	0	0	1	-2
0	0	0	0	-2	1
0	0	0	0	2	1
0	0	0	0	1	-2
0	0	1	0	1	3
1	1	1	0	6	3
-3	-3	-3	-3	-2	-2
-3	-3	-3	-3	-2	-2
-3	-3	-3	-3	-2	-2
-3	-3	-3	-3	-2	-2
-3	-3	-3	-3	-2	-2
1	0	0	0	1	2
0	1	1	0	3	-2
-3	-3	-3	-3	-2	-2
0	0	0	0	1	-2
0	0	0	0	1	-2
-3	-3	-3	-3	-2	-2
0	0	0	0	-2	1
1	1	1	0	4	-2
1	1	1	0	5	4
0	0	0	0	-2	1
1	1	1	0	1	5
1	1	0	0	-2	-2
0	0	0	0	1	-2
1	1	1	1	3	4
1	1	1	0	-2	1
0	1	1	0	-2	2
0	0	0	0	-2	1
0	1	1	0	3	-2
1	1	0	0	4	3
1	0	1	0	1	-2
1	0	0	0	1	-2
0	1	0	0	-2	-2
1	1	1	0	-2	-2
-3	-3	-3	-3	-2	-2
-3	-3	-3	-3	-2	-2
0	0	0	0	-2	1
1	0	0	0	-2	-2
0	0	1	0	-2	-2

12. (Prospective co	12. (Case-control	12. (Cross-secti	12. (Case rep	12. (Other
-2	-2	-2	-2	-2
-2	-2	-2	-2	-2
-2	-2	-2	-2	-2
-2	-2	-2	-2	-2
1	-2	-2	-2	-2
-2	-2	-2	-2	3
1	-2	2	-2	-2
-2	-2	-2	-2	-2
2	-2	3	-2	-2
5	-2	3	4	-2
-2	-2	1	3	2
-2	2	1	-2	-2
2	-2	-2	-2	-2
2	4	5	6	-2
3	4	5	6	-2
-2	-2	-2	-2	-2
-2	-2	-2	-2	-2
-2	1	-2	2	-2
-2	-2	-2	-2	-2
-2	-2	-2	-2	-2
-2	-2	-2	-2	-2
-2	-2	1	-2	-2
-2	2	1	-2	-2
-2	-2	-2	-2	-2
-2	-2	-2	-2	-2
3	1	2	-2	-2
-2	-2	-2	-2	-2
-2	-2	-2	1	-2
2	-2	3	-2	-2
-2	-2	-2	-2	-2
1	3	2	-2	-2
-2	1	-2	2	-2
4	1	3	2	-2
-2	-2	-2	-2	-2
1	-2	-2	3	-2
4	1	2	3	-2
3	-2	-2	-2	-2
-2	-2	-2	-2	-2
3	2	5	4	7
-2	-2	-2	-2	-2
3	1	2	-2	-2
-2	-2	-2	2	-2
-2	-2	-2	-2	-2
-2	-2	-2	-2	-2
-2	2	1	-2	-2
-2	-2	2	-2	3
-2	-2	-2	-2	1
2	-2	-2	-2	-2
-2	-2	-2	-2	-2
2	5	6	7	1
1	-2	-2	-2	-2
-2	-2	1	-2	-2
-2	-2	-2	-2	-2
-2	1	2	3	-2
2	3	4	-2	-2

-2	1	2	-2	-2
-2	3	1	-2	-2
2	4	3	5	-2
2	-2	-2	-2	-2
-2	-2	-2	-2	-2
-2	-2	-2	-2	1
5	1	2	3	-2
3	5	1	6	7
-2	-2	-2	-2	-2
-2	1	2	-2	-2
-2	-2	-2	-2	-2
1	-2	-2	-2	-2
2	3	-2	4	-2
1	-2	-2	-2	-2
2	-2	-2	3	-2
4	5	-2	3	-2
-2	2	-2	-2	-2
-2	-2	-2	-2	-2
-2	-2	-2	-2	-2
-2	-2	-2	-2	-2
-2	-2	-2	-2	-2
-2	1	2	3	-2
-2	2	1	3	-2
-2	-2	-2	-2	-2
1	2	-2	3	-2
-2	-2	-2	-2	-2
-2	-2	-2	-2	-2
1	-2	-2	-2	-2
3	2	-2	1	-2
5	2	3	1	-2
-2	-2	-2	-2	-2
-2	-2	-2	-2	-2
-2	-2	-2	-2	-2
3	2	-2	-2	-2
-2	1	-2	-2	-2
-2	-2	-2	-2	-2
-2	-2	-2	-2	-2
-2	-2	-2	-2	-2
3	1	2	4	7
-2	2	-2	-2	-2
-2	-2	-2	-2	-2
3	-2	-2	-2	-2
-2	-2	3	-2	-2
1	-2	-2	-2	-2
3	4	-2	5	-2
-2	-2	-2	-2	-2
-2	-2	-2	-2	-2
1	5	2	-2	-2
5	1	3	4	-2
-2	1	-2	-2	-2
-2	3	2	1	-2
-2	2	-2	-2	-2
-2	-2	-2	-2	-2
3	1	2	-2	-2
-2	-2	-2	-2	-2
-2	-2	-2	-2	-2

-2	1	-2	-2	-2
2	-2	-2	-2	-2
-2	-2	1	-2	-2
-2	-2	-2	-2	-2
-2	1	2	3	-2
-2	-2	-2	1	-2
-2	-2	-2	-2	-2
2	3	5	4	-2
-2	1	-2	-2	-2
-2	-2	-2	-2	-2
-2	-2	-2	-2	-2
2	5	1	6	-2
-2	-2	-2	1	-2
-2	2	1	-2	-2
-2	1	-2	2	-2
-2	-2	-2	-2	-2
1	-2	3	-2	-2
-2	-2	-2	-2	-2
-2	-2	-2	-2	-2
-2	-2	-2	1	-2
-2	2	1	-2	-2
2	4	5	6	-2
1	2	-2	-2	3
-2	1	-2	-2	-2
-2	-2	-2	-2	-2
-2	2	3	1	-2
-2	-2	-2	-2	-2
-2	-2	-2	-2	-2
-2	-2	2	-2	-2
-2	-2	-2	1	-2
-2	1	-2	2	-2
-2	-2	-2	-2	-2
-2	-2	-2	-2	-2
1	-2	-2	-2	-2
3	1	2	-2	-2
-2	-2	3	-2	-2
-2	2	-2	1	-2
-2	-2	-2	3	-2
-2	2	3	-2	-2
3	5	4	6	-2
2	-2	-2	-2	-2
3	-2	-2	-2	-2
1	-2	-2	-2	-2
4	1	3	2	7
-2	2	-2	1	-2
-2	-2	-2	2	1
-2	-2	-2	-2	-2
-2	-2	-2	-2	-2
-2	-2	-2	-2	-2
-2	-2	-2	-2	-2
-2	-2	-2	-2	-2
-2	-2	-2	-2	-2
-2	-2	-2	-2	-2
1	-2	-2	-2	-2
3	-2	4	5	-2
-2	-2	-2	-2	-2

-2	-2	-2	-2	-2
-2	-2	-2	-2	-2
1	-2	2	-2	-2
6	1	3	2	7
2	-2	3	-2	-2
2	-2	-2	-2	-2
-2	-2	-2	-2	-2
-2	-2	-2	-2	-2
-2	-2	-2	1	-2
-2	-2	-2	-2	-2
1	2	-2	-2	-2
-2	3	-2	-2	-2
2	-2	-2	-2	-2
-2	-2	-2	-2	-2
-2	-2	-2	-2	-2
-2	-2	-2	-2	-2
2	1	-2	-2	-2
-2	-2	1	-2	-2
1	2	3	4	-2
1	2	4	-2	-2
2	-2	-2	-2	-2
-2	-2	-2	-2	-2
1	-2	-2	-2	-2
1	-2	-2	-2	-2
3	-2	-2	-2	-2
-2	-2	-2	-2	-2
-2	-2	-2	-2	-2
-2	1	-2	-2	-2
-2	-2	-2	-2	-2
-2	-2	-2	-2	-2
-2	1	-2	-2	-2
-2	-2	-2	-2	-2
-2	-2	-2	-2	-2
-2	-2	-2	-2	-2
-2	-2	-2	-2	-2
1	-2	-2	-2	-2
2	-2	3	-2	-2
1	-2	-2	-2	-2
-2	-2	-2	-2	-2
-2	1	-2	-2	-2
2	1	-2	3	-2
2	4	6	5	-2
3	-2	2	-2	-2
-2	-2	-2	-2	1
-2	2	1	-2	-2
-2	-2	-2	-2	-2
1	-2	-2	-2	-2
-2	-2	1	-2	-2
-2	-2	-2	-2	-2
-2	2	-2	-2	-2
-2	-2	-2	-2	-2
-2	-2	-2	-2	-2
-2	-2	-2	-2	-2
-2	-2	1	-2	-2
-2	2	3	1	-2
-2	2	1	3	-2

-2	-2	-2	-2	-2
-2	-2	-2	-2	-2
1	5	2	-2	-2
1	-2	2	-2	-2
1	4	5	-2	-2
-2	-2	-2	1	-2
-2	-2	-2	-2	-2
2	-2	-2	-2	3
-2	-2	-2	-2	-2
2	4	-2	-2	-2
2	-2	-2	-2	-2
-2	-2	-2	-2	-2
-2	-2	-2	-2	-2
5	3	7	4	6
-2	-2	-2	-2	-2
-2	-2	-2	-2	-2
-2	-2	-2	-2	-2
-2	-2	-2	-2	-2
-2	-2	-2	-2	-2
-2	-2	-2	-2	-2
2	3	1	-2	-2
4	6	5	7	2
-2	-2	-2	-2	2
2	-2	-2	-2	-2
2	-2	-2	-2	1
-2	-2	-2	-2	-2
-2	-2	-2	-2	-2
-2	-2	-2	-2	-2
-2	-2	-2	1	-2
-2	-2	-2	-2	-2
-2	-2	1	-2	-2
-2	-2	-2	-2	-2
2	5	4	-2	-2
-2	-2	-2	-2	-2
2	-2	1	-2	-2
-2	-2	-2	-2	-2
-2	-2	-2	-2	-2
-2	2	-2	-2	-2
-2	-2	-2	-2	-2
-2	-2	-2	-2	-2
-2	-2	-2	-2	-2
-2	-2	-2	-2	-2
-2	-2	1	2	-2
-2	-2	-2	-2	-2
1	-2	-2	-2	-2
-2	-2	-2	-2	-2
-2	-2	-2	-2	-2
3	4	-2	-2	5
-2	-2	-2	-2	2
-2	-2	-2	-2	-2
-2	-2	-2	-2	-2
4	3	-2	-2	-2
2	-2	-2	-2	-2
-2	-2	-2	-2	-2
-2	-2	-2	-2	-2
-2	-2	-2	-2	-2

2	-2	-2	-2	-2
2	-2	-2	-2	-2
1	-2	-2	-2	-2
-2	-2	-2	-2	-2
1	-2	-2	-2	-2
3	-2	-2	-2	2
-2	-2	-2	-2	-2
-2	-2	-2	-2	-2
-2	-2	-2	-2	-2
-2	-2	-2	-2	1
1	4	-2	-2	-2
-2	-2	-2	-2	-2
-2	-2	-2	-2	-2
3	4	5	-2	-2
-2	-2	-2	-2	-2
-2	-2	1	2	-2
-2	3	-2	4	-2
-2	-2	-2	-2	-2
-2	-2	-2	-2	-2
-2	3	1	-2	-2
1	-2	-2	2	-2
3	5	4	6	-2
-2	-2	3	-2	-2
4	-2	2	1	-2
-2	1	-2	2	-2
-2	-2	-2	-2	-2
3	4	5	6	-2
3	5	1	6	2
-2	1	-2	-2	-2
-2	1	-2	2	-2
-2	-2	-2	2	-2
-2	-2	-2	-2	-2
3	2	1	-2	-2
3	5	4	6	-2
2	-2	1	-2	-2
-2	-2	-2	-2	-2
-2	1	2	-2	3
1	-2	-2	-2	-2
3	5	2	4	7
1	-2	-2	-2	-2
-2	3	-2	-2	-2
-2	-2	-2	-2	-2
4	3	6	5	-2
-2	-2	3	1	-2
-2	2	-2	3	-2
-2	-2	-2	-2	-2
-2	-2	-2	3	-2
1	3	4	-2	-2
-2	3	2	1	-2
-2	-2	1	-2	-2
-2	-2	-2	-2	-2
-2	-2	-2	-2	-2
2	4	3	5	-2
-2	-2	-2	-2	-2
-2	-2	-2	-2	-2
-2	-2	-2	-2	-2

1	-2	2	-2	-2
-2	-2	-2	-2	-2
5	1	2	3	-2
3	-2	2	-2	-2
3	1	-2	-2	-2
3	4	-2	5	-2
-2	-2	-2	-2	-2
2	-2	-2	-2	4
-2	1	3	-2	-2
-2	-2	-2	-2	-2
3	-2	-2	1	-2
-2	-2	3	-2	-2
1	4	3	-2	-2
-2	-2	-2	-2	-2
-2	1	-2	-2	-2
-2	-2	-2	-2	-2
-2	-2	-2	-2	-2
3	-2	-2	-2	-2
2	-2	-2	-2	-2
2	-2	-2	4	-2
4	2	1	5	7
-2	-2	-2	-2	-2
-2	-2	-2	-2	-2
-2	-2	-2	-2	-2
-2	-2	-2	-2	-2
-2	-2	-2	-2	-2
-2	3	-2	-2	-2
1	-2	2	4	-2
-2	-2	-2	-2	-2
-2	-2	-2	-2	-2
-2	-2	-2	-2	-2
-2	-2	-2	-2	-2
-2	-2	-2	-2	-2
-2	1	2	3	-2
6	1	2	3	-2
2	-2	-2	-2	-2
2	3	4	6	-2
-2	1	2	-2	-2
-2	-2	-2	-2	-2
2	1	5	6	-2
-2	2	3	4	-2
1	4	3	-2	-2
-2	-2	-2	-2	-2
1	-2	2	4	-2
-2	2	1	-2	-2
-2	-2	-2	-2	-2
-2	2	-2	-2	-2
-2	-2	1	-2	-2
-2	1	-2	-2	-2
-2	-2	-2	-2	-2
-2	-2	-2	-2	-2
-2	-2	-2	-2	-2
-2	1	-2	-2	-2
-2	-2	-2	1	-2

13. Have you published any SCI articles? If yes,
-3
-3
2
-3
2
-3
-3
-3
2
2
2
-3
2
4
2
6
-3
2
-3
1
5
4
2
2
-3
6
1
1
2
-3
6
3
2
-3
3
5
3
-3
6
-3
3
6
5
5
3
2
-3
-3
-3
-3
-3
2
1
2
6

2
-3
4
4
2
1
2
1
-3
1
-3
2
-3
2
-3
6
2
-3
-3
1
-3
2
2
-3
1
-3
-3
2
3
2
-3
-3
-3
2
1
-3
2
-3
6
-3
-3
-3
1
3
6
-3
-3
-3
2
-3
1
2
2
-3
-3
-3

2
2
1
-3
1
1
-3
2
1
2
-3
6
1
2
1
-3
2
3
-3
6
2
-3
-3
-3
-3
3
-3
-3
3
1
2
-3
-3
2
3
3
3
-3
2
2
6
6
6
1
2
1
-3
-3
-3
-3
4
-3
2
-3
2
-3

-3
-3
6
2
2
2
-3
-3
2
-3
-3
-3
-3
-3
-3
1
2
2
6
6
1
-3
2
2
3
6
-3
3
-3
-3
2
4
-3
-3
-3
-3
1
2
-3
2
2
6
-3
1
1
-3
2
1
2
2
2
1
2
2
2
2

-3
2
4
3
2
1
2
-3
-3
1
-3
3
-3
-3
-3
-3
-3
-3
-3
-3
2
-3
-3
-3
-3
-3
2
2
-3
-3
-3
-3
-3
-3
1
-3
-3
2
-3
-3
-3
-3
3
-3
-3
-3
-3
-3
3
-3
-3
-3
-3
-3
-3
-3

-3
-3
-3
2
-3
6
-3
-3
-3
-3
-3
1
2
-3
-3
-3
3
2
-3
2
3
-3
2
3
2
4
2
3
2
2
1
-3
6
3
6
3
-3
2
4
3
-3
2
2
2
6
2
3
4
3
5
-3
1
2
-3
-3
-3

2
-3
2
6
1
3
-3
3
2
-3
2
6
2
-3
6
2
1
-3
3
-3
2
-3
-3
-3
-3
-3
2
4
-3
2
2
-3
2
2
1
3
6
2
-3
2
2
6
2
5
-3
1
1
2
3
-3
-3
2
1
2

14. Have you published SCI articles as first author or/and correspond
-3
-3
1
-3
1
-3
-3
-3
2
1
1
-3
1
1
1
4
-3
1
-3
1
4
2
1
1
-3
3
3
1
1
-3
1
2
1
-3
2
2
2
-3
5
-3
2
3
3
4
2
1
-3
-3
-3
-3
-3
1
1
1
4

1
-3
2
2
1
1
1
1
-3
1
-3
1
-3
1
-3
2
1
-3
-3
1
-3
1
1
-3
1
-3
-3
1
2
1
-3
-3
-3
1
1
-3
1
-3
5
-3
-3
-3
1
1
2
-3
-3
-3
1
-3
1
1
1
-3
-3
-3

2
1
2
-3
1
1
-3
2
1
2
-3
6
1
2
1
-3
2
3
-3
6
2
-3
-3
-3
-3
3
-3
-3
3
1
2
-3
-3
2
2
3
3
-3
3
2
6
6
6
1
2
1
-3
-3
-3
-3
5
-3
2
-3
2
-3

-3
-3
5
1
2
2
-3
-3
2
-3
-3
-3
-3
-3
-3
1
1
2
3
6
1
-3
2
2
3
6
-3
2
-3
-3
2
4
-3
-3
-3
-3
1
2
-3
2
2
6
-3
1
1
-3
2
1
2
2
2
1
2
2
1
1

-3
2
3
3
2
1
4
-3
-3
1
-3
3
-3
-3
-3
-3
-3
-3
-3
-3
2
-3
-3
-3
-3
-3
2
2
-3
-3
-3
-3
-3
-3
1
-3
-3
2
-3
-3
-3
-3
2
-3
-3
-3
-3
-3
3
-3
-3
-3
-3
-3
-3
-3

-3
-3
-3
2
-3
4
-3
-3
-3
-3
-3
1
4
-3
-3
-3
3
2
-3
2
3
-3
2
2
2
4
2
2
2
1
1
-3
4
2
4
2
-3
2
4
3
-3
2
2
2
4
2
2
2
2
3
-3
1
2
-3
-3
-3

2
-3
2
4
1
3
-3
2
2
-3
2
6
1
-3
6
2
1
-3
2
-3
2
-3
-3
-3
-3
-3
2
3
-3
2
2
-3
2
2
1
3
5
2
-3
2
2
2
1
5
-3
1
1
2
3
-3
-3
2
1
2

15. In your current job, which of the following woul	16. (Design of clinical r	16. (Writing clinical res
-3	-2	-2
2	1	2
3	1	2
-3	-2	-2
3	2	-2
1	1	2
1	1	2
1	1	3
1	1	2
6	2	3
2	1	3
2	1	2
1	-2	2
1	2	4
1	1	2
1	1	-2
-3	-2	-2
1	1	3
-3	-2	-2
1	1	2
4	2	1
2	5	1
2	2	3
3	1	2
2	1	-2
2	1	-2
1	1	2
1	1	2
2	1	-2
-3	-2	-2
1	1	3
2	3	5
3	2	-2
-3	-2	-2
2	1	2
1	1	3
3	2	4
-3	-2	-2
1	1	2
1	1	2
1	1	-2
1	1	2
2	4	5
2	1	2
2	1	2
2	1	2
3	-2	-2
6	1	2
-3	-2	-2
3	1	5
1	1	2
2	3	4
1	1	2
5	1	4
2	2	1

2	2	1
1	1	2
2	1	-2
1	1	3
3	1	2
1	1	2
1	2	3
2	1	2
1	2	3
2	2	-2
-3	-2	-2
1	3	6
1	1	2
2	-2	1
2	1	2
1	1	2
2	-2	1
1	1	2
1	1	2
4	1	-2
-3	-2	-2
2	2	3
3	2	3
-3	-2	-2
2	1	3
1	1	2
-3	-2	-2
2	1	2
1	2	3
2	1	2
-3	-2	-2
-3	-2	-2
-3	-2	-2
1	2	3
2	2	3
-3	-2	-2
2	1	2
-3	-2	-2
2	4	5
1	1	-2
-3	-2	-2
1	1	2
3	1	2
2	2	5
1	2	4
1	1	5
-3	-2	-2
1	5	4
3	1	3
2	1	2
3	4	3
2	1	2
3	1	-2
6	-2	2
-3	-2	-2
-3	-2	-2

1	7	1
3	1	2
2	1	-2
-3	-2	-2
2	5	2
1	1	2
1	2	3
4	1	2
1	2	3
1	3	4
-3	-2	-2
1	1	2
1	1	2
1	1	2
2	1	4
-3	-2	-2
2	1	2
2	1	2
-3	-2	-2
1	1	-2
2	2	5
6	-2	-2
2	1	7
3	1	3
1	1	-2
3	2	1
-3	-2	-2
2	1	2
2	1	2
2	1	6
1	1	2
-3	-2	-2
-3	-2	-2
1	1	2
1	1	2
1	1	-2
2	1	2
1	3	4
3	1	2
3	2	4
1	1	2
1	1	2
1	2	5
2	1	3
1	1	-2
1	1	3
1	1	2
-3	-2	-2
-3	-2	-2
-3	-2	-2
2	4	2
-3	-2	-2
1	1	4
1	1	3
2	1	2
-3	-2	-2

1	-2	-2
1	1	2
1	1	2
2	1	2
1	1	2
2	3	1
-3	-2	-2
-3	-2	-2
2	2	1
2	1	2
5	-2	-2
1	1	-2
1	1	2
-3	-2	-2
-3	-2	-2
3	1	2
2	1	2
2	1	3
1	1	2
2	2	1
4	-2	-2
-3	-2	-2
2	1	2
2	1	2
2	1	2
1	-2	1
1	1	4
4	1	-2
-3	-2	-2
-3	-2	-2
4	1	2
2	1	2
-3	-2	-2
2	-2	-2
1	1	2
1	1	2
2	1	2
2	1	2
-3	-2	-2
1	1	2
2	1	2
2	2	3
1	-2	-2
2	-2	-2
2	2	3
-3	-2	-2
1	1	2
1	1	3
1	1	2
2	1	2
6	1	2
2	1	3
1	1	2
2	1	-2
1	2	5
1	2	4

1	1	2
1	1	4
1	1	2
4	2	3
1	-2	-2
4	1	3
2	3	4
1	1	2
-3	-2	-2
2	1	-2
2	1	4
1	1	2
2	1	2
1	1	5
1	1	-2
-3	-2	-2
-3	-2	-2
-3	-2	-2
1	2	3
-3	-2	-2
2	1	3
1	1	-2
1	1	3
1	1	2
1	-2	-2
-3	-2	-2
1	1	2
1	1	-2
2	-2	-2
1	1	2
3	-2	-2
1	1	2
1	1	5
-3	-2	-2
2	2	3
1	2	3
6	1	-2
1	1	-2
1	1	-2
1	1	3
2	2	3
1	1	2
2	1	5
-3	-2	-2
1	1	-2
1	1	7
1	1	3
5	1	-2
6	-2	1
-3	-2	-2
1	1	-2
1	1	3
1	-2	-2
-3	-2	-2
3	1	2
-3	-2	-2

2	1	2
1	1	3
2	2	-2
2	1	-2
1	1	2
6	3	4
-3	-2	-2
-3	-2	-2
-3	-2	-2
1	1	2
2	2	1
2	1	-2
2	1	2
2	1	2
-3	-2	-2
2	1	4
3	1	2
1	4	1
-3	-2	-2
3	2	3
1	1	2
2	2	3
1	1	2
2	1	5
1	1	3
1	1	2
2	3	4
2	1	2
2	1	2
4	1	2
5	1	2
-3	-2	-2
1	1	2
2	1	2
1	1	2
2	1	2
1	1	2
1	1	2
2	2	3
2	1	-2
1	1	4
1	1	2
5	1	2
1	1	2
2	1	2
2	1	-2
1	1	2
2	1	2
1	1	2
2	1	2
-3	-2	-2
5	3	2
2	1	5
-3	-2	-2
1	1	2
-3	-2	-2

1	3	4
-3	-2	-2
1	2	4
1	1	-2
2	1	2
1	1	2
-3	-2	-2
1	-2	2
2	1	2
-3	-2	-2
2	1	2
2	1	2
2	1	3
-3	-2	-2
2	1	2
4	1	2
1	3	2
1	1	3
1	1	5
1	1	4
2	1	2
-3	-2	-2
-3	-2	-2
-3	-2	-2
-3	-2	-2
-3	-2	-2
1	1	2
1	1	2
-3	-2	-2
2	2	3
3	1	2
-3	-2	-2
2	1	3
3	1	-2
2	1	2
2	1	2
1	2	3
2	2	-2
1	-2	-2
1	1	5
2	1	3
1	4	3
1	1	2
1	1	2
6	1	2
3	1	2
1	2	1
2	1	3
1	2	3
-3	-2	-2
-3	-2	-2
2	1	2
2	1	2
1	-2	-2

16. (Statistical analysis of clinical research data	16. (Clinical research e	16. (Implementation and ma
-2	-2	-2
3	-2	4
3	4	5
-2	-2	-2
1	-2	-2
3	4	5
3	4	5
6	7	2
3	4	5
4	-2	5
2	-2	-2
3	4	5
-2	-2	1
3	5	1
3	4	5
-2	-2	2
-2	-2	-2
4	6	2
-2	-2	-2
3	-2	-2
3	-2	-2
2	3	4
1	7	4
3	4	5
-2	-2	-2
2	-2	3
3	4	5
-2	-2	-2
2	-2	-2
-2	-2	-2
7	5	4
1	7	2
-2	-2	-2
-2	-2	-2
-2	-2	3
4	5	2
1	-2	5
-2	-2	-2
3	-2	-2
5	4	3
2	-2	3
-2	-2	3
2	1	3
3	-2	-2
-2	-2	-2
-2	-2	-2
1	-2	3
3	-2	-2
-2	-2	-2
2	6	7
-2	3	4
2	5	1
3	5	6
-2	-2	-2
3	7	6

5	4	3
4	7	3
2	-2	3
-2	-2	2
3	-2	-2
3	-2	4
1	4	5
6	4	5
1	-2	-2
-2	-2	1
-2	-2	-2
7	1	2
3	4	5
-2	-2	2
4	7	3
3	4	5
2	-2	4
3	4	5
5	3	-2
-2	-2	-2
-2	-2	-2
7	1	4
1	6	5
-2	-2	-2
4	6	2
3	-2	-2
-2	-2	-2
3	-2	-2
1	6	4
3	-2	-2
-2	-2	-2
-2	-2	-2
-2	-2	-2
6	5	1
1	7	4
-2	-2	-2
5	3	6
-2	-2	-2
1	6	2
2	-2	3
-2	-2	-2
-2	-2	-2
3	-2	-2
1	3	4
1	5	3
7	3	2
-2	-2	-2
1	2	3
2	7	4
3	4	5
5	6	7
-2	-2	3
2	-2	-2
-2	-2	3
-2	-2	-2
-2	-2	-2

2	3	4
-2	-2	3
2	-2	4
-2	-2	-2
6	7	4
-2	-2	-2
-2	-2	1
6	5	3
7	4	6
2	5	1
-2	-2	-2
3	5	4
3	4	5
3	5	4
2	6	7
-2	-2	-2
3	-2	-2
3	-2	-2
-2	-2	-2
-2	-2	-2
1	3	4
-2	-2	-2
2	3	4
-2	-2	2
2	-2	5
4	5	3
-2	-2	-2
3	5	4
3	-2	-2
2	7	5
5	6	4
-2	-2	-2
-2	-2	-2
6	7	3
-2	-2	3
2	-2	-2
-2	3	4
7	2	1
-2	-2	3
1	5	7
3	4	5
3	4	5
3	4	1
2	7	4
-2	-2	2
-2	-2	2
-2	-2	-2
-2	-2	-2
-2	-2	-2
-2	-2	-2
3	1	5
-2	-2	-2
2	3	-2
2	7	5
7	6	5
-2	-2	-2

-2	2	1
3	4	5
-2	-2	3
3	4	5
3	-2	4
6	5	2
-2	-2	-2
-2	-2	-2
-2	-2	-2
-2	3	4
-2	-2	1
3	-2	2
3	-2	-2
-2	-2	-2
-2	-2	-2
3	7	5
-2	-2	-2
-2	-2	2
-2	-2	3
5	3	4
-2	1	2
-2	-2	-2
3	-2	4
3	-2	4
3	4	6
-2	-2	-2
3	5	2
-2	-2	-2
-2	-2	-2
-2	-2	-2
3	-2	-2
3	-2	4
-2	-2	-2
1	-2	-2
3	-2	-2
3	-2	-2
3	4	5
-2	-2	-2
-2	-2	-2
4	5	3
3	4	5
4	5	1
-2	-2	1
-2	-2	-2
-2	-2	-2
-2	-2	-2
-2	3	4
2	7	4
3	7	5
-2	-2	3
-2	3	-2
2	-2	-2
3	-2	4
-2	-2	-2
3	7	4
3	7	5

3	4	5
2	5	3
3	-2	4
-2	-2	-2
-2	-2	1
6	7	2
1	5	2
3	-2	-2
-2	-2	-2
-2	-2	2
3	-2	2
3	4	5
-2	-2	3
2	6	7
-2	-2	2
-2	-2	-2
-2	-2	-2
-2	-2	-2
4	5	1
-2	-2	-2
2	-2	-2
2	-2	4
2	-2	4
3	4	5
2	-2	1
-2	-2	-2
3	4	5
2	-2	3
2	-2	3
3	4	5
-2	-2	1
3	4	5
2	4	6
-2	-2	-2
-2	4	1
5	7	1
-2	-2	-2
2	-2	-2
-2	-2	-2
2	-2	-2
1	4	5
5	4	3
2	4	3
-2	-2	-2
2	3	4
2	3	4
5	2	4
-2	-2	4
2	-2	3
-2	-2	-2
-2	-2	-2
2	4	-2
-2	1	2
-2	-2	-2
3	4	5
-2	-2	-2

3	4	5
2	5	4
-2	-2	1
-2	-2	-2
3	-2	-2
5	1	2
-2	-2	-2
-2	-2	-2
-2	-2	-2
3	-2	-2
5	4	3
-2	-2	-2
-2	-2	3
3	-2	4
-2	-2	-2
2	5	3
3	4	5
3	5	6
-2	-2	-2
4	7	1
3	4	5
1	7	6
4	6	3
3	4	2
2	-2	-2
7	5	3
5	7	6
6	7	3
3	4	-2
3	-2	-2
3	-2	5
-2	-2	-2
-2	-2	-2
3	5	4
3	-2	-2
3	4	5
-2	-2	-2
3	-2	4
1	6	4
-2	-2	-2
2	7	3
5	4	3
3	7	4
5	6	7
3	4	5
-2	-2	-2
4	-2	-2
-2	-2	-2
3	-2	-2
4	6	3
-2	-2	-2
1	-2	-2
2	7	4
-2	-2	-2
-2	-2	-2
-2	-2	-2

5	6	1
-2	-2	-2
3	7	1
-2	-2	-2
4	6	3
3	-2	-2
-2	-2	-2
-2	-2	1
3	7	6
-2	-2	-2
3	-2	4
-2	-2	3
2	7	6
-2	-2	-2
3	4	-2
5	3	4
-2	1	-2
5	-2	2
2	4	3
5	6	7
3	4	5
-2	-2	-2
-2	-2	-2
-2	-2	-2
-2	-2	-2
-2	-2	-2
3	-2	4
-2	-2	3
-2	-2	-2
1	4	5
3	-2	-2
-2	-2	-2
-2	-2	2
3	-2	2
7	6	3
3	-2	4
5	6	4
-2	1	-2
-2	2	1
4	6	2
-2	-2	2
2	-2	-2
3	4	-2
3	4	5
-2	4	3
3	-2	-2
3	5	4
6	5	2
4	6	1
-2	-2	-2
-2	-2	-2
3	4	5
4	-2	-2
-2	-2	-2

16. (Thesis writing a	16. (Literature revie	16. (Other	17、What is your preferred
-2	-2	-2	-3
-2	-2	-2	2
6	7	8	4
-2	-2	-2	-3
-2	-2	-2	4
6	7	-2	1
6	7	-2	3
4	5	-2	3
-2	-2	-2	2
1	-2	-2	4
-2	-2	-2	3
-2	-2	-2	2
-2	-2	-2	3
6	7	-2	3
-2	-2	-2	4
3	-2	-2	3
-2	-2	-2	-3
5	7	-2	3
-2	-2	-2	-3
4	-2	-2	3
4	-2	-2	3
-2	-2	-2	3
5	6	-2	4
6	7	-2	4
-2	-2	-2	3
-2	-2	-2	1
6	-2	-2	2
-2	-2	-2	4
-2	-2	-2	3
-2	-2	-2	-3
6	2	-2	1
4	6	-2	3
1	3	-2	4
-2	-2	-2	-3
4	-2	-2	3
6	7	-2	3
3	-2	-2	3
-2	-2	-2	-3
-2	-2	-2	1
6	7	-2	3
-2	-2	-2	2
4	-2	-2	2
6	7	-2	2
4	-2	-2	3
-2	-2	-2	4
3	-2	-2	3
-2	2	-2	2
-2	-2	-2	4
-2	-2	-2	-3
3	4	8	2
-2	-2	-2	2
7	6	-2	3
4	7	-2	2
2	3	-2	2
4	5	-2	4

6	7	-2	4
5	6	-2	3
-2	-2	-2	3
-2	-2	-2	2
4	-2	-2	4
5	-2	-2	4
6	7	-2	3
3	7	-2	3
-2	-2	-2	2
-2	-2	-2	3
-2	-2	-2	-3
5	4	-2	3
-2	-2	-2	1
-2	-2	-2	2
5	6	-2	1
6	7	-2	2
3	-2	-2	3
6	7	-2	3
-2	4	-2	4
-2	-2	-2	4
-2	-2	-2	-3
5	6	-2	2
4	7	-2	4
-2	-2	-2	-3
5	7	-2	3
4	-2	-2	3
-2	-2	-2	-3
-2	-2	-2	4
7	5	-2	2
-2	-2	-2	3
-2	-2	-2	-3
-2	-2	-2	-3
-2	-2	-2	-3
4	7	-2	4
5	6	-2	3
-2	-2	-2	-3
4	7	-2	3
-2	-2	-2	-3
7	3	-2	3
-2	-2	-2	2
-2	-2	-2	-3
-2	3	-2	4
-2	-2	-2	1
7	6	-2	2
6	7	-2	3
4	6	-2	3
-2	-2	-2	-3
6	7	-2	2
5	6	-2	3
6	7	-2	4
2	1	-2	4
4	-2	-2	4
3	-2	-2	2
-2	4	1	2
-2	-2	-2	-3
-2	-2	-2	-3

5	6	-2	3
4	-2	-2	3
3	-2	-2	3
-2	-2	-2	-3
3	1	-2	4
3	-2	-2	3
-2	-2	-2	4
4	7	-2	2
5	1	-2	3
6	7	-2	3
-2	-2	-2	-3
6	-2	-2	3
6	7	-2	4
6	7	-2	3
3	5	-2	2
-2	-2	-2	-3
4	5	-2	1
-2	-2	-2	2
-2	-2	-2	-3
-2	-2	-2	2
7	6	-2	4
-2	-2	1	3
5	6	-2	3
-2	-2	-2	2
3	4	-2	4
6	7	-2	4
-2	-2	-2	-3
7	6	-2	4
-2	-2	-2	1
3	4	-2	3
3	7	-2	3
-2	-2	-2	-3
-2	-2	-2	-3
4	5	-2	3
-2	4	-2	3
-2	-2	-2	1
-2	-2	-2	3
6	5	-2	4
-2	-2	-2	3
3	6	-2	3
6	7	-2	3
6	7	-2	3
-2	-2	-2	3
5	6	8	3
-2	-2	-2	4
-2	-2	-2	3
-2	-2	-2	3
-2	-2	-2	-3
-2	-2	-2	-3
-2	-2	-2	-3
-2	-2	-2	3
-2	-2	-2	-3
-2	-2	-2	2
4	6	-2	3
3	4	-2	1
-2	-2	-2	-3

-2	-2	-2	4
-2	-2	-2	3
-2	-2	-2	1
6	7	-2	2
-2	-2	-2	3
7	4	-2	3
-2	-2	-2	-3
-2	-2	-2	-3
3	-2	-2	3
5	-2	-2	2
-2	-2	-2	3
-2	-2	-2	3
-2	-2	-2	1
-2	-2	-2	-3
-2	-2	-2	-3
4	6	-2	2
-2	-2	-2	3
-2	-2	-2	3
-2	-2	-2	2
6	7	-2	3
-2	-2	-2	2
-2	-2	-2	-3
5	6	-2	1
5	-2	-2	4
5	7	-2	4
-2	-2	-2	2
-2	-2	-2	4
-2	-2	-2	4
-2	-2	-2	-3
-2	-2	-2	-3
4	5	-2	4
5	-2	-2	4
-2	-2	-2	-3
-2	-2	-2	3
-2	-2	-2	4
-2	-2	-2	3
6	7	-2	4
3	4	-2	3
-2	-2	-2	-3
6	7	-2	3
6	7	-2	4
6	7	-2	2
2	3	-2	3
1	-2	-2	3
1	-2	-2	3
-2	-2	-2	-3
-2	-2	-2	4
6	5	-2	2
4	6	-2	4
4	-2	-2	4
-2	4	-2	1
-2	-2	-2	3
5	6	-2	4
-2	-2	-2	4
1	6	-2	4
1	6	-2	4

6	7	-2	3
6	7	-2	2
5	-2	-2	2
1	-2	-2	4
-2	-2	-2	2
5	4	-2	3
6	7	-2	3
4	5	-2	3
-2	-2	-2	-3
-2	-2	-2	1
-2	-2	-2	3
6	7	-2	3
-2	-2	-2	4
3	4	-2	3
-2	-2	-2	1
-2	-2	-2	-3
-2	-2	-2	-3
-2	-2	-2	-3
6	7	-2	3
-2	-2	-2	-3
4	-2	-2	2
-2	3	-2	2
-2	-2	5	3
6	7	-2	4
-2	-2	-2	1
-2	-2	-2	-3
6	7	-2	4
-2	-2	-2	2
-2	1	-2	1
6	7	-2	1
-2	-2	-2	3
-2	-2	-2	3
-2	3	-2	4
-2	-2	-2	-3
5	-2	-2	4
4	6	-2	1
-2	-2	-2	3
3	-2	-2	2
-2	-2	-2	4
-2	-2	-2	3
6	7	-2	3
7	6	-2	2
-2	-2	-2	3
-2	-2	-2	-3
5	-2	-2	3
5	6	-2	3
-2	-2	-2	3
2	3	-2	3
-2	-2	-2	3
-2	-2	-2	-3
-2	-2	-2	2
-2	-2	-2	2
-2	-2	-2	2
-2	-2	-2	-3
6	7	-2	3
-2	-2	-2	-3

-2	-2	-2	4
6	7	-2	2
-2	-2	-2	3
-2	-2	-2	2
-2	-2	-2	2
6	-2	-2	3
-2	-2	-2	-3
-2	-2	-2	-3
-2	-2	-2	-3
-2	-2	-2	3
-2	-2	-2	3
-2	-2	-2	2
4	-2	-2	2
-2	-2	-2	2
-2	-2	-2	-3
7	6	-2	1
6	7	-2	3
2	-2	-2	1
-2	-2	-2	-3
6	5	-2	3
6	7	-2	3
5	4	-2	3
5	7	-2	3
6	7	-2	3
-2	-2	-2	3
4	6	-2	2
2	1	-2	3
4	5	-2	3
-2	-2	-2	2
-2	-2	-2	4
4	-2	-2	2
-2	-2	-2	-3
-2	-2	-2	4
6	7	-2	3
-2	-2	-2	2
6	7	-2	2
3	4	-2	2
-2	-2	-2	2
5	7	8	3
3	2	-2	2
5	6	-2	3
6	7	-2	4
5	6	-2	1
3	4	-2	3
-2	-2	-2	4
-2	-2	-2	2
3	-2	-2	3
-2	3	-2	2
4	-2	-2	1
5	-2	-2	2
-2	-2	-2	-3
4	-2	-2	4
3	6	-2	4
-2	-2	-2	-3
3	4	-2	3
-2	-2	-2	-3

2	7	-2	3
-2	-2	-2	-3
6	5	-2	4
-2	-2	-2	3
5	7	-2	3
-2	-2	-2	3
-2	-2	-2	-3
-2	-2	-2	2
4	5	-2	3
-2	-2	-2	-3
-2	-2	-2	4
-2	-2	-2	2
4	5	-2	3
-2	-2	-2	-3
5	-2	-2	2
6	-2	-2	2
-2	-2	-2	4
4	-2	-2	1
7	6	-2	3
3	2	-2	4
6	7	-2	4
-2	-2	-2	-3
-2	-2	-2	-3
-2	-2	-2	-3
-2	-2	-2	-3
-2	-2	-2	-3
-2	-2	-2	4
-2	-2	-2	4
-2	-2	-2	-3
-2	-2	-2	2
-2	-2	-2	3
-2	-2	-2	-3
-2	-2	-2	3
4	-2	-2	4
5	4	-2	3
-2	-2	-2	2
1	7	-2	1
-2	3	-2	2
-2	-2	-2	4
3	7	-2	4
-2	-2	-2	4
-2	1	-2	4
-2	-2	-2	2
-2	-2	-2	1
-2	-2	-2	4
4	-2	-2	3
6	7	-2	4
7	4	-2	3
5	7	-2	3
-2	-2	-2	-3
-2	-2	-2	-3
6	7	-2	4
3	-2	-2	3
1	-2	-2	3

18. Please rank the group learning styles you prefer	19. Would you prefer to atter	20. (Obtaining a trainin
-3	-3	-2
1	1	-2
1	2	-2
-3	-3	-2
2	4	-2
1	1	-2
3	4	-2
3	2	-2
1	4	4
3	2	1
1	4	2
1	4	-2
1	4	4
1	4	4
3	2	1
1	4	-2
-3	-3	-2
3	2	4
-3	-3	-2
2	2	-2
2	4	-2
3	3	1
2	3	-2
3	3	-2
2	4	-2
1	1	-2
1	1	-2
1	3	-2
2	4	3
-3	-3	-2
1	1	4
1	4	4
1	4	5
-3	-3	-2
3	3	-2
2	2	-2
2	4	1
-3	-3	-2
1	4	-2
2	2	-2
1	1	3
1	4	-2
1	4	-2
1	1	-2
3	2	-2
2	4	-2
1	4	-2
3	2	-2
-3	-3	-2
1	1	-2
1	4	-2
3	4	-2
1	4	-2
1	1	5
2	2	-2

3	2	4
2	4	-2
1	2	3
1	4	-2
3	3	2
2	3	-2
3	2	4
2	4	4
1	4	-2
2	4	-2
-3	-3	-2
2	4	-2
1	1	-2
1	1	-2
2	4	-2
1	1	-2
3	4	-2
2	3	-2
1	4	-2
3	2	-2
-3	-3	-2
1	4	-2
3	2	4
-3	-3	-2
1	4	2
2	2	-2
-3	-3	-2
2	3	-2
1	4	1
3	2	3
-3	-3	-2
-3	-3	-2
-3	-3	-2
2	2	3
1	4	-2
-3	-3	-2
3	2	4
-3	-3	-2
3	4	-2
1	1	-2
-3	-3	-2
1	3	-2
1	3	-2
1	4	4
3	4	4
3	2	-2
-3	-3	-2
1	1	-2
1	2	3
2	4	-2
3	2	4
3	2	3
1	1	-2
1	4	-2
-3	-3	-2
-3	-3	-2

1	3	4
1	4	-2
3	3	-2
-3	-3	-2
2	2	1
2	2	3
2	3	-2
1	4	1
3	3	4
2	4	3
-3	-3	-2
1	4	-2
2	3	5
3	2	4
1	4	4
-3	-3	-2
1	4	3
2	3	-2
-3	-3	-2
1	1	-2
2	2	4
2	3	-2
3	4	-2
2	4	-2
3	2	-2
2	1	-2
-3	-3	-2
3	4	-2
1	1	-2
2	4	3
1	3	5
-3	-3	-2
-3	-3	-2
1	4	4
2	4	-2
1	1	-2
1	4	-2
3	3	-2
1	1	-2
1	4	4
1	1	-2
1	1	-2
1	4	-2
3	4	3
2	4	-2
1	2	-2
2	4	-2
-3	-3	-2
-3	-3	-2
-3	-3	-2
3	4	-2
-3	-3	-2
1	4	1
2	2	-2
1	1	4
-3	-3	-2

3	3	-2
1	4	-2
1	1	-2
1	4	4
2	2	-2
3	2	4
-3	-3	-2
-3	-3	-2
2	3	-2
1	3	-2
3	2	-2
3	2	-2
1	1	-2
-3	-3	-2
-3	-3	-2
1	4	4
1	2	1
1	4	-2
1	4	-2
1	4	-2
1	4	1
-3	-3	-2
1	4	-2
2	4	2
2	2	-2
1	3	-2
2	3	-2
3	4	4
-3	-3	-2
-3	-3	-2
3	2	-2
3	3	-2
-3	-3	-2
2	3	-2
1	3	-2
3	4	-2
2	4	1
2	2	1
-3	-3	-2
3	3	5
3	4	3
1	4	-2
2	4	-2
3	2	1
2	4	3
-3	-3	-2
3	2	-2
1	1	3
3	4	2
3	3	-2
1	1	2
2	3	-2
3	2	-2
2	3	-2
2	3	4
2	3	4

3	4	-2
2	3	1
1	1	-2
2	2	-2
1	4	1
1	4	-2
1	4	-2
3	4	-2
-3	-3	-2
1	4	-2
3	2	-2
1	2	-2
3	2	-2
3	4	-2
1	4	-2
-3	-3	-2
-3	-3	-2
-3	-3	-2
2	4	-2
-3	-3	-2
1	4	-2
1	4	-2
2	2	-2
3	2	-2
1	1	-2
-3	-3	-2
3	2	5
3	3	-2
1	1	-2
1	1	-2
2	3	-2
2	2	-2
3	4	-2
-3	-3	-2
2	3	4
1	1	-2
2	2	-2
1	4	-2
3	2	-2
1	4	-2
2	3	-2
1	4	-2
3	4	2
-3	-3	-2
2	4	-2
3	4	-2
2	4	-2
2	4	-2
2	4	-2
-3	-3	-2
1	1	-2
1	4	-2
1	3	-2
-3	-3	-2
2	4	-2
-3	-3	-2

2	4	-2
1	4	-2
3	2	-2
1	1	1
1	4	-2
3	4	-2
-3	-3	-2
-3	-3	-2
-3	-3	-2
1	4	-2
3	4	-2
1	3	4
1	4	-2
3	4	-2
-3	-3	-2
1	4	-2
1	4	-2
1	1	2
-3	-3	-2
1	3	3
2	4	-2
2	4	-2
1	4	-2
2	1	4
3	2	-2
1	4	-2
3	2	-2
3	2	4
1	4	1
3	2	2
3	4	3
-3	-3	-2
3	3	-2
2	4	2
1	4	-2
3	2	1
3	1	-2
1	1	-2
2	4	-2
3	4	-2
2	3	-2
2	2	4
1	1	3
2	2	4
1	3	-2
1	1	1
2	4	3
1	4	3
1	2	4
2	4	-2
-3	-3	-2
2	2	-2
3	2	4
-3	-3	-2
3	2	-2
-3	-3	-2

1	2	4
-3	-3	-2
2	4	5
2	2	-2
1	4	3
2	2	-2
-3	-3	-2
1	4	-2
2	3	4
-3	-3	-2
3	2	-2
2	4	-2
2	4	5
-3	-3	-2
1	1	-2
1	4	-2
2	1	-2
1	4	-2
3	3	3
3	4	-2
3	2	-2
-3	-3	-2
-3	-3	-2
-3	-3	-2
-3	-3	-2
-3	-3	-2
3	4	4
3	2	-2
-3	-3	-2
1	1	1
3	3	-2
-3	-3	-2
1	2	-2
3	4	3
2	4	5
1	4	-2
1	1	-2
1	4	-2
3	2	-2
3	3	4
3	4	3
3	4	-2
1	4	2
1	1	-2
2	3	-2
2	2	-2
2	2	4
1	4	4
2	3	-2
-3	-3	-2
-3	-3	-2
3	3	3
3	4	4
1	3	-2

20. (Publication of SCI pap	20. (Continuing educ	20. (Establishment of coop	20. (Access to clinical res
-2	-2	-2	-2
-2	-2	-2	-2
-2	-2	-2	1
-2	-2	-2	-2
1	-2	-2	2
-2	-2	-2	-2
-2	-2	-2	-2
-2	-2	-2	-2
2	5	1	3
2	-2	-2	3
1	5	3	4
-2	-2	-2	-2
1	-2	3	2
1	3	2	5
-2	-2	2	-2
-2	-2	-2	-2
-2	-2	-2	-2
2	5	3	1
-2	-2	-2	-2
-2	-2	-2	1
-2	-2	-2	-2
3	-2	2	-2
2	-2	3	1
1	-2	-2	2
-2	-2	-2	-2
-2	-2	-2	-2
-2	-2	-2	-2
1	-2	2	3
2	-2	4	1
-2	-2	-2	-2
3	-2	1	2
1	5	3	2
3	4	2	1
-2	-2	-2	-2
-2	-2	1	2
3	-2	1	2
4	-2	2	3
-2	-2	-2	-2
-2	-2	-2	-2
-2	-2	-2	-2
1	-2	-2	2
-2	-2	-2	-2
-2	-2	-2	-2
-2	-2	-2	-2
1	-2	2	3
1	-2	3	2
-2	-2	-2	-2
-2	-2	-2	-2
-2	-2	-2	-2
-2	-2	-2	-2
-2	-2	-2	-2
4	3	2	1
1	-2	3	2
4	1	3	2
-2	-2	-2	-2

2	5	3	1
-2	-2	-2	-2
4	-2	1	2
-2	-2	1	-2
1	-2	4	3
1	-2	2	3
2	5	3	1
3	5	2	1
-2	-2	-2	-2
1	-2	-2	-2
-2	-2	-2	-2
1	-2	2	3
-2	-2	-2	-2
1	-2	-2	-2
-2	-2	-2	-2
1	-2	3	2
2	-2	1	-2
-2	-2	-2	-2
-2	-2	-2	-2
1	-2	-2	-2
-2	-2	-2	-2
-2	-2	-2	-2
2	5	3	1
-2	-2	-2	-2
1	-2	3	4
-2	-2	-2	-2
-2	-2	-2	-2
1	-2	-2	2
2	-2	3	-2
1	4	5	2
-2	-2	-2	-2
-2	-2	-2	-2
-2	-2	-2	-2
2	1	-2	4
2	-2	3	1
-2	-2	-2	-2
1	5	2	3
-2	-2	-2	-2
-2	-2	-2	-2
-2	-2	-2	-2
-2	-2	-2	-2
-2	-2	-2	-2
1	2	-2	-2
2	5	1	3
2	5	1	3
-2	-2	-2	-2
-2	-2	-2	-2
-2	-2	-2	-2
1	4	5	2
-2	-2	-2	-2
1	3	5	2
2	-2	-2	1
1	-2	3	2
-2	-2	-2	-2
-2	-2	-2	-2
-2	-2	-2	-2

1	5	3	2
1	-2	3	2
1	-2	2	3
-2	-2	-2	-2
3	5	2	4
1	5	4	2
-2	-2	-2	-2
3	5	2	4
1	5	2	3
4	5	2	1
-2	-2	-2	-2
-2	-2	-2	-2
3	4	2	1
2	5	3	1
1	5	2	3
-2	-2	-2	-2
-2	-2	2	1
-2	-2	-2	-2
-2	-2	-2	-2
-2	-2	-2	-2
1	5	3	2
-2	-2	-2	-2
-2	-2	-2	-2
-2	-2	-2	-2
-2	-2	-2	-2
-2	-2	-2	-2
-2	-2	-2	-2
-2	-2	-2	-2
-2	-2	-2	-2
1	-2	4	2
1	4	2	3
-2	-2	-2	-2
-2	-2	-2	-2
1	-2	3	2
2	-2	1	3
-2	-2	-2	-2
-2	-2	-2	-2
-2	-2	-2	-2
-2	-2	-2	-2
3	5	2	1
-2	-2	-2	-2
-2	-2	-2	-2
-2	-2	-2	-2
1	2	4	5
1	-2	2	-2
1	-2	2	-2
-2	-2	-2	-2
-2	-2	-2	-2
-2	-2	-2	-2
-2	-2	-2	-2
-2	-2	-2	-2
-2	-2	-2	-2
4	5	3	2
-2	-2	-2	-2
2	5	3	1
-2	-2	-2	-2

-2	-2	-2	-2
-2	-2	-2	-2
-2	-2	-2	-2
3	5	1	2
2	-2	3	1
1	5	2	3
-2	-2	-2	-2
-2	-2	-2	-2
1	-2	2	-2
-2	-2	-2	-2
-2	-2	-2	-2
-2	-2	-2	-2
-2	-2	-2	-2
-2	-2	-2	-2
-2	-2	-2	-2
1	5	2	3
2	-2	-2	-2
1	-2	2	3
-2	-2	-2	-2
-2	-2	-2	-2
-2	2	-2	-2
-2	-2	-2	-2
2	-2	-2	1
3	-2	1	4
-2	-2	-2	-2
-2	-2	-2	-2
-2	-2	-2	-2
1	5	2	3
-2	-2	-2	-2
-2	-2	-2	-2
1	-2	2	3
-2	-2	-2	-2
-2	-2	-2	-2
-2	-2	-2	-2
-2	-2	-2	-2
-2	-2	-2	-2
2	-2	-2	3
-2	-2	2	3
-2	-2	-2	-2
2	4	3	1
2	4	-2	1
-2	-2	-2	-2
-2	-2	-2	-2
-2	-2	-2	-2
1	-2	-2	2
-2	-2	-2	-2
-2	-2	-2	1
4	5	1	2
3	-2	4	1
2	-2	-2	1
-2	-2	3	1
3	-2	1	2
1	-2	3	2
1	-2	-2	-2
1	5	2	3
1	5	2	3

-2	-2	-2	-2
2	-2	-2	-2
-2	-2	-2	-2
-2	-2	-2	-2
2	3	4	5
1	-2	2	3
-2	-2	-2	-2
-2	-2	-2	-2
-2	-2	-2	-2
-2	-2	-2	-2
-2	-2	-2	-2
-2	-2	-2	-2
-2	-2	-2	-2
-2	-2	-2	-2
-2	-2	-2	-2
-2	-2	-2	-2
-2	-2	-2	-2
-2	-2	-2	-2
-2	-2	-2	-2
-2	-2	-2	-2
1	-2	2	3
-2	-2	-2	-2
-2	-2	-2	-2
-2	-2	-2	-2
-2	-2	-2	-2
-2	-2	-2	-2
1	2	4	3
1	-2	2	3
-2	-2	-2	-2
-2	-2	-2	-2
-2	-2	-2	-2
-2	-2	-2	-2
-2	-2	-2	-2
-2	-2	-2	-2
3	-2	1	2
-2	-2	-2	-2
-2	-2	-2	-2
1	-2	2	-2
-2	-2	-2	-2
-2	-2	-2	-2
-2	-2	-2	-2
-2	-2	-2	-2
1	5	4	3
-2	-2	-2	-2
-2	-2	-2	-2
-2	-2	-2	-2
-2	-2	-2	-2
-2	-2	-2	-2
-2	-2	-2	-2
-2	-2	-2	-2
-2	-2	-2	-2
-2	-2	-2	-2
-2	-2	-2	-2
-2	-2	-2	-2
-2	-2	-2	-2
-2	-2	-2	-2

-2	-2	-2	-2
-2	-2	-2	-2
-2	-2	-2	-2
2	-2	3	-2
-2	-2	-2	-2
-2	-2	-2	-2
-2	-2	-2	-2
-2	-2	-2	-2
-2	-2	-2	-2
-2	-2	-2	-2
-2	-2	-2	-2
1	-2	2	3
-2	-2	-2	-2
-2	-2	-2	-2
-2	-2	-2	-2
-2	-2	-2	-2
-2	-2	-2	-2
1	-2	-2	3
-2	-2	-2	-2
2	5	1	4
-2	-2	-2	-2
-2	-2	-2	-2
1	-2	2	3
1	5	3	2
1	-2	-2	2
-2	-2	-2	-2
-2	-2	1	2
3	5	1	2
2	-2	-2	-2
-2	-2	3	1
2	5	1	4
-2	-2	-2	-2
-2	-2	-2	-2
4	5	1	3
-2	-2	-2	-2
2	3	4	5
-2	-2	-2	-2
-2	-2	2	1
-2	-2	-2	-2
-2	-2	-2	-2
-2	-2	-2	-2
1	5	2	3
1	2	4	5
1	5	3	2
-2	-2	-2	-2
2	-2	-2	3
4	-2	2	1
-2	-2	2	1
1	-2	2	3
-2	-2	-2	-2
-2	-2	-2	-2
-2	-2	-2	1
2	5	1	3
-2	-2	-2	-2
-2	-2	-2	-2
-2	-2	-2	-2

1	5	3	2
-2	-2	-2	-2
1	4	3	2
-2	-2	-2	-2
1	5	2	4
-2	-2	-2	-2
-2	-2	-2	-2
1	-2	2	3
1	5	2	3
-2	-2	-2	-2
3	-2	1	2
-2	-2	-2	-2
3	4	1	2
-2	-2	-2	-2
-2	-2	-2	-2
1	-2	2	3
1	-2	2	3
-2	-2	-2	-2
4	5	2	1
-2	-2	-2	-2
1	-2	2	3
-2	-2	-2	-2
-2	-2	-2	-2
-2	-2	-2	-2
-2	-2	-2	-2
-2	-2	-2	-2
1	-2	2	3
-2	-2	-2	-2
-2	-2	-2	-2
2	-2	-2	-2
-2	-2	1	-2
-2	-2	-2	-2
-2	-2	1	2
-2	-2	1	2
1	2	3	4
-2	-2	-2	-2
-2	-2	-2	-2
3	-2	2	1
-2	-2	-2	-2
1	5	3	2
2	-2	-2	1
-2	-2	2	1
1	-2	-2	-2
-2	-2	-2	-2
-2	-2	-2	-2
1	-2	2	3
2	5	3	1
1	5	3	2
-2	-2	-2	-2
-2	-2	-2	-2
-2	-2	-2	-2
2	5	4	1
1	5	2	3
1	-2	2	3

20. (Other	21. (Obtaining a trainin	21. (Publication of SCI pap	21. (Establishment of coop
-2	-2	-2	-2
-2	-2	-2	2
-2	-2	-2	-2
-2	-2	-2	-2
-2	-2	-2	-2
-2	-2	-2	-2
-2	1	2	3
-2	-2	1	3
-2	-2	-2	-2
-2	-2	-2	-2
-2	-2	-2	-2
-2	-2	-2	-2
-2	-2	-2	-2
-2	-2	-2	-2
-2	-2	-2	-2
-2	-2	-2	-2
-2	-2	-2	-2
-2	-2	-2	-2
-2	-2	-2	-2
-2	-2	-2	-2
-2	-2	-2	-2
-2	-2	-2	-2
-2	-2	-2	-2
-2	-2	-2	-2
-2	-2	-2	-2
-2	-2	-2	-2
-2	-2	-2	-2
-2	-2	-2	-2
-2	-2	-2	-2
-2	-2	-2	-2
-2	-2	-2	-2
-2	-2	-2	-2
-2	-2	-2	-2
-2	-2	-2	-2
-2	-2	-2	-2
-2	-2	-2	-2
-2	-2	-2	-2
-2	-2	-2	-2
-2	-2	-2	-2
-2	5	4	3
-2	-2	-2	-2
-2	-2	-2	-2
-2	-2	-2	-2
-2	-2	-2	-2
-2	-2	-2	-2
-2	-2	-2	-2
-2	-2	-2	-2
-2	2	-2	4
-2	-2	-2	-2
-2	6	4	2
-2	-2	-2	-2
-2	-2	-2	-2
-2	-2	-2	-2
-2	-2	-2	-2
-2	-2	-2	-2

-2	-2	-2	-2
-2	-2	-2	-2
-2	-2	-2	-2
-2	-2	-2	-2
-2	-2	-2	-2
-2	-2	-2	-2
-2	-2	-2	-2
-2	-2	-2	-2
-2	2	-2	-2
-2	-2	-2	-2
-2	-2	-2	-2
-2	-2	-2	-2
-2	2	1	3
-2	-2	-2	-2
-2	-2	-2	-2
-2	-2	-2	-2
-2	-2	-2	-2
-2	5	3	4
-2	3	-2	4
-2	-2	-2	-2
-2	-2	-2	-2
1	-2	-2	-2
-2	-2	-2	-2
-2	-2	-2	-2
-2	-2	-2	-2
-2	4	3	2
-2	-2	-2	-2
-2	-2	-2	-2
-2	-2	-2	-2
-2	-2	-2	-2
-2	-2	-2	-2
-2	-2	-2	-2
-2	-2	-2	-2
-2	-2	-2	-2
-2	-2	-2	-2
-2	-2	-2	-2
-2	-2	-2	-2
-2	-2	-2	-2
-2	-2	-2	-2
-2	-2	-2	2
-2	-2	-2	-2
-2	-2	-2	-2
-2	-2	-2	-2
-2	-2	-2	-2
-2	-2	-2	-2
-2	-2	-2	-2
-2	-2	-2	-2
-2	4	5	1
-2	-2	-2	-2
-2	-2	-2	-2
-2	-2	-2	-2
-2	-2	-2	-2
-2	-2	-2	-2
-2	-2	-2	-2
-2	-2	-2	-2
-2	-2	-2	-2

-2	-2	-2	-2
-2	-2	-2	-2
-2	-2	-2	-2
-2	-2	-2	-2
-2	-2	-2	-2
-2	-2	-2	-2
-2	-2	-2	2
-2	-2	-2	-2
-2	-2	-2	-2
-2	-2	-2	-2
-2	-2	-2	-2
-2	-2	-2	-2
-2	-2	-2	-2
-2	-2	-2	-2
-2	-2	-2	-2
-2	-2	-2	-2
-2	-2	-2	-2
-2	-2	-2	-2
-2	-2	-2	-2
-2	-2	-2	-2
-2	-2	-2	-2
-2	-2	-2	2
-2	4	5	1
-2	-2	-2	-2
-2	5	2	4
-2	-2	-2	-2
-2	-2	-2	-2
-2	1	4	5
-2	-2	-2	-2
-2	-2	-2	-2
-2	-2	-2	-2
-2	-2	-2	-2
-2	-2	-2	-2
-2	-2	-2	-2
-2	-2	-2	-2
-2	-2	-2	-2
-2	-2	-2	-2
-2	3	-2	2
-2	-2	-2	-2
-2	-2	-2	-2
-2	-2	-2	-2
-2	-2	-2	-2
-2	-2	-2	-2
-2	-2	-2	-2
-2	-2	-2	-2
-2	-2	-2	-2
-2	-2	-2	-2
-2	-2	-2	-2
-2	-2	-2	-2
-2	-2	-2	-2
-2	-2	-2	-2
-2	-2	-2	-2
-2	-2	-2	-2
-2	-2	-2	-2
-2	-2	-2	-2
-2	-2	-2	-2

-2	-2	-2	-2
-2	1	-2	-2
-2	-2	-2	-2
-2	-2	-2	-2
-2	-2	-2	-2
-2	-2	-2	-2
-2	-2	-2	-2
-2	-2	-2	-2
-2	-2	-2	-2
-2	1	2	4
-2	3	4	5
-2	-2	-2	-2
-2	-2	3	2
-2	-2	-2	-2
-2	-2	-2	-2
-2	-2	-2	-2
-2	-2	-2	-2
-2	-2	-2	-2
-2	-2	-2	-2
-2	-2	-2	-2
-2	-2	-2	-2
-2	-2	-2	-2
-2	-2	-2	-2
-2	-2	-2	-2
-2	-2	-2	-2
-2	-2	-2	-2
-2	3	-2	-2
-2	-2	-2	-2
-2	-2	-2	-2
-2	-2	-2	-2
-2	-2	-2	-2
-2	-2	-2	-2
-2	-2	-2	-2
-2	-2	-2	-2
-2	1	-2	2
-2	-2	-2	-2
-2	-2	-2	-2
-2	-2	-2	-2
-2	-2	-2	-2
-2	-2	-2	-2
-2	-2	-2	-2
-2	-2	-2	-2
-2	-2	-2	-2
-2	-2	-2	-2
-2	-2	-2	-2
-2	-2	-2	-2
-2	-2	-2	-2
-2	-2	-2	-2
-2	-2	-2	-2
-2	-2	-2	-2
-2	-2	-2	-2
-2	-2	-2	-2
-2	-2	-2	-2
-2	-2	-2	-2
6	-2	-2	-2
-2	-2	-2	-2

-2	1	-2	2
-2	-2	-2	-2
-2	-2	-2	-2
-2	-2	-2	-2
-2	-2	-2	-2
-2	-2	-2	-2
-2	-2	-2	-2
-2	2	-2	-2
-2	-2	-2	-2
1	-2	-2	-2
-2	5	3	2
-2	-2	-2	-2
-2	1	-2	-2
-2	1	2	4
-2	1	-2	-2
-2	-2	-2	-2
-2	-2	-2	-2
-2	-2	-2	-2
-2	4	5	1
-2	-2	-2	-2
-2	-2	-2	-2
-2	4	-2	3
-2	-2	-2	-2
-2	2	1	3
-2	-2	-2	2
-2	-2	-2	-2
-2	-2	-2	-2
-2	-2	-2	-2
-2	-2	-2	1
-2	-2	-2	2
-2	-2	-2	1
-2	-2	-2	2
-2	4	-2	1
-2	-2	-2	-2
-2	-2	-2	-2
-2	5	4	2
-2	-2	-2	-2
-2	-2	-2	-2
-2	4	5	3
-2	1	3	2
-2	-2	-2	-2
-2	-2	-2	-2
-2	-2	-2	-2
-2	-2	-2	-2
-2	4	-2	2
-2	1	4	2
-2	-2	-2	3
-2	-2	2	-2
-2	-2	-2	-2
-2	-2	-2	-2
-2	-2	-2	-2
-2	4	-2	2
-2	1	-2	-2
-2	-2	-2	-2
-2	-2	-2	-2
-2	-2	-2	-2

-2	1	-2	2
-2	5	4	1
-2	-2	-2	-2
-2	-2	-2	-2
-2	-2	-2	1
-2	-2	-2	-2
-2	-2	-2	-2
-2	-2	-2	-2
-2	-2	-2	-2
-2	-2	1	-2
-2	4	5	3
-2	-2	-2	-2
-2	-2	-2	-2
-2	-2	-2	2
-2	-2	-2	-2
-2	-2	-2	-2
-2	-2	-2	-2
-2	-2	-2	-2
-2	-2	-2	-2
-2	-2	-2	-2
-2	-2	-2	-2
-2	-2	-2	1
-2	-2	-2	-2
-2	-2	-2	-2
-2	-2	-2	-2
-2	-2	-2	-2
-2	-2	-2	-2
-2	-2	-2	-2
-2	-2	-2	-2
-2	-2	-2	-2
-2	-2	-2	-2
-2	-2	-2	-2
-2	-2	-2	-2
-2	-2	-2	-2
-2	-2	-2	-2
-2	-2	-2	-2
-2	-2	2	1
-2	-2	-2	-2
-2	-2	-2	-2
-2	-2	-2	-2
-2	4	5	3
-2	-2	-2	-2
-2	-2	-2	-2
-2	-2	-2	-2
-2	-2	-2	-2
-2	-2	-2	-2
-2	-2	-2	-2
-2	-2	-2	-2
-2	-2	-2	-2
-2	-2	-2	-2
-2	-2	-2	-2
-2	-2	-2	-2
-2	-2	-2	-2
-2	-2	-2	-2
-2	-2	1	-2
-2	-2	-2	-2

-2	-2	-2	-2
-2	-2	-2	-2
-2	-2	-2	-2
-2	-2	-2	-2
-2	-2	-2	-2
-2	-2	-2	-2
-2	-2	-2	-2
-2	-2	-2	-2
-2	-2	-2	-2
-2	-2	-2	-2
-2	-2	-2	-2
-2	-2	-2	-2
-2	-2	-2	-2
-2	-2	-2	-2
-2	-2	-2	-2
-2	-2	-2	-2
-2	-2	-2	-2
-2	-2	-2	-2
-2	-2	-2	-2
-2	5	4	2
-2	-2	-2	-2
-2	-2	-2	-2
-2	-2	-2	-2
-2	-2	-2	-2
-2	-2	-2	-2
-2	-2	-2	-2
-2	-2	-2	-2
-2	-2	-2	-2
-2	-2	-2	-2
-2	-2	-2	-2
-2	-2	-2	-2
-2	-2	-2	-2
-2	-2	-2	-2
-2	-2	-2	-2
-2	-2	-2	-2
-2	-2	-2	-2
-2	-2	-2	-2
-2	-2	-2	-2
-2	-2	-2	2
-2	-2	-2	-2
-2	-2	-2	-2
-2	-2	-2	-2
-2	-2	-2	-2
-2	-2	-2	-2
-2	5	4	3
-2	-2	-2	-2
-2	-2	-2	-2
-2	-2	-2	-2
-2	-2	-2	-2
-2	-2	-2	-2
-2	-2	-2	-2
-2	-2	-2	-2
-2	-2	-2	-2
-2	-2	-2	-2

21. (Meet high-quality eco-pa	21. (Access to clinical res	21. (Other	22. (Obtaining a trainin
-2	-2	-2	-2
3	1	-2	-2
-2	-2	-2	-2
-2	-2	-2	-2
-2	-2	-2	-2
-2	-2	-2	-2
5	4	-2	-2
4	2	-2	-2
-2	-2	-2	-2
-2	-2	-2	-2
-2	-2	-2	-2
-2	-2	-2	1
-2	-2	-2	-2
-2	-2	-2	-2
-2	-2	-2	-2
-2	-2	-2	-2
-2	-2	-2	-2
-2	-2	-2	-2
-2	-2	-2	-2
-2	-2	-2	-2
-2	-2	-2	-2
-2	-2	-2	-2
-2	-2	-2	-2
-2	-2	-2	-2
-2	-2	-2	-2
-2	-2	-2	-2
-2	-2	-2	-2
-2	-2	-2	-2
-2	-2	-2	-2
-2	-2	-2	-2
-2	-2	-2	-2
-2	-2	-2	-2
-2	-2	-2	-2
-2	-2	-2	-2
-2	-2	-2	-2
-2	-2	-2	-2
-2	-2	-2	-2
-2	-2	-2	-2
-2	-2	-2	-2
2	1	-2	-2
-2	-2	-2	-2
-2	-2	-2	-2
-2	-2	-2	-2
-2	-2	-2	-2
-2	-2	-2	-2
-2	-2	-2	-2
-2	-2	-2	-2
3	1	-2	-2
-2	-2	-2	-2
3	1	5	-2
-2	-2	-2	-2
-2	-2	-2	-2
-2	-2	-2	-2
-2	-2	-2	-2
-2	-2	-2	-2

-2	-2	-2	-2
-2	-2	-2	-2
-2	-2	-2	-2
-2	-2	-2	-2
-2	-2	-2	-2
-2	-2	-2	-2
-2	-2	-2	-2
-2	-2	-2	-2
3	1	-2	-2
-2	-2	-2	-2
-2	-2	-2	-2
-2	-2	-2	-2
4	-2	-2	-2
-2	-2	-2	-2
-2	-2	-2	-2
-2	-2	-2	-2
-2	-2	-2	-2
2	1	-2	-2
1	2	-2	-2
-2	-2	-2	-2
-2	-2	-2	-2
-2	-2	-2	-2
-2	-2	-2	-2
-2	-2	-2	-2
-2	-2	-2	-2
-2	1	-2	-2
-2	-2	-2	-2
-2	-2	-2	-2
-2	-2	-2	-2
-2	-2	-2	-2
-2	-2	-2	-2
-2	-2	-2	-2
-2	-2	-2	-2
-2	-2	-2	-2
-2	-2	-2	-2
-2	-2	-2	-2
-2	-2	-2	-2
-2	-2	-2	-2
-2	-2	-2	-2
3	1	-2	-2
-2	-2	-2	-2
-2	1	-2	-2
-2	-2	-2	-2
-2	-2	-2	-2
-2	-2	-2	-2
-2	-2	-2	-2
-2	-2	-2	-2
2	3	-2	-2
-2	-2	-2	-2
-2	-2	-2	-2
-2	-2	-2	-2
-2	-2	-2	-2
-2	-2	-2	-2
-2	-2	-2	-2
-2	-2	-2	-2
-2	-2	-2	-2

-2	-2	-2	-2
-2	-2	-2	-2
-2	-2	-2	-2
-2	-2	-2	-2
-2	-2	-2	-2
-2	-2	-2	-2
1	3	-2	-2
-2	-2	-2	-2
-2	-2	-2	-2
-2	-2	-2	-2
-2	-2	-2	-2
-2	-2	-2	-2
-2	-2	-2	-2
-2	-2	-2	-2
-2	-2	-2	-2
-2	-2	-2	-2
-2	-2	-2	-2
-2	-2	-2	-2
-2	-2	-2	-2
-2	-2	-2	-2
-2	-2	-2	-2
1	-2	-2	-2
3	2	-2	-2
-2	-2	-2	-2
3	1	-2	-2
-2	-2	-2	-2
-2	-2	-2	-2
3	2	-2	-2
-2	-2	-2	-2
-2	-2	-2	-2
-2	-2	-2	-2
-2	-2	-2	-2
-2	-2	-2	-2
-2	-2	-2	-2
-2	-2	-2	-2
-2	-2	-2	-2
-2	-2	-2	-2
1	4	-2	-2
-2	-2	-2	-2
-2	-2	-2	-2
-2	-2	-2	-2
-2	-2	-2	-2
-2	-2	-2	-2
-2	-2	-2	-2
-2	-2	-2	-2
-2	-2	-2	-2
-2	-2	-2	-2
-2	-2	-2	-2
-2	-2	-2	-2
-2	-2	-2	-2
-2	-2	-2	-2
-2	-2	-2	-2
-2	-2	-2	-2
-2	-2	-2	-2
-2	-2	-2	-2
-2	-2	-2	-2

-2	-2	-2	-2
2	3	-2	-2
-2	-2	-2	-2
-2	-2	-2	-2
-2	-2	-2	-2
-2	-2	-2	-2
-2	-2	-2	-2
-2	-2	-2	-2
-2	-2	-2	-2
3	-2	-2	-2
1	2	-2	-2
-2	-2	-2	-2
-2	1	-2	-2
-2	-2	-2	-2
-2	-2	-2	-2
-2	-2	-2	-2
-2	-2	-2	-2
-2	-2	-2	-2
-2	-2	-2	-2
-2	-2	-2	-2
-2	-2	-2	-2
-2	-2	-2	-2
-2	-2	-2	-2
-2	-2	-2	-2
-2	-2	-2	-2
-2	-2	-2	-2
2	1	-2	-2
-2	-2	-2	-2
-2	-2	-2	-2
-2	-2	-2	-2
-2	-2	-2	-2
-2	-2	-2	-2
-2	-2	-2	-2
-2	-2	-2	-2
3	4	-2	-2
-2	1	-2	-2
-2	-2	-2	-2
-2	-2	-2	-2
-2	-2	-2	-2
-2	-2	-2	-2
-2	-2	-2	-2
-2	-2	-2	-2
-2	-2	-2	-2
-2	-2	-2	-2
-2	-2	-2	-2
-2	-2	-2	-2
-2	-2	-2	-2
-2	-2	-2	-2
-2	-2	-2	-2
-2	-2	-2	-2
-2	-2	-2	-2
-2	-2	-2	-2
-2	-2	-2	-2
-2	-2	-2	-2
-2	-2	-2	-2
-2	-2	-2	-2

4	3	-2	-2
-2	-2	-2	-2
-2	-2	-2	-2
-2	-2	-2	-2
-2	-2	-2	-2
-2	-2	-2	-2
-2	-2	-2	-2
-2	1	-2	-2
-2	-2	-2	-2
-2	-2	-2	-2
1	4	-2	-2
-2	-2	-2	-2
-2	2	-2	-2
5	3	-2	-2
-2	2	-2	-2
-2	-2	-2	-2
-2	-2	-2	-2
-2	-2	-2	-2
2	3	-2	-2
-2	-2	-2	-2
-2	-2	-2	-2
2	1	-2	-2
2	1	-2	-2
5	4	-2	-2
3	1	-2	-2
-2	-2	-2	-2
-2	-2	-2	-2
-2	-2	-2	-2
-2	2	-2	-2
1	3	-2	-2
-2	-2	-2	-2
3	1	-2	-2
3	2	-2	-2
-2	-2	-2	-2
-2	-2	-2	-2
1	3	-2	-2
1	-2	-2	-2
-2	-2	-2	-2
2	1	-2	-2
-2	-2	-2	-2
-2	-2	-2	-2
-2	-2	-2	-2
-2	-2	-2	-2
-2	-2	-2	-2
1	3	-2	-2
5	3	-2	-2
2	1	-2	-2
-2	1	-2	-2
-2	-2	-2	-2
-2	-2	-2	-2
-2	-2	-2	-2
1	3	-2	-2
-2	2	-2	-2
-2	-2	-2	-2
-2	1	-2	-2
-2	-2	-2	-2

-2	3	-2	-2
2	3	-2	-2
-2	1	-2	-2
-2	-2	-2	-2
-2	2	-2	-2
-2	-2	-2	-2
-2	-2	-2	-2
-2	-2	-2	-2
-2	-2	-2	-2
-2	-2	-2	-2
2	1	-2	-2
-2	-2	-2	-2
-2	-2	-2	-2
-2	1	-2	-2
-2	-2	-2	-2
-2	-2	-2	-2
-2	-2	-2	-2
-2	-2	-2	-2
-2	-2	-2	-2
-2	-2	-2	-2
-2	-2	-2	-2
-2	2	-2	-2
-2	-2	-2	-2
-2	-2	-2	-2
-2	-2	-2	-2
-2	-2	-2	-2
-2	-2	-2	-2
-2	-2	-2	-2
-2	-2	-2	-2
-2	-2	-2	-2
-2	-2	-2	-2
-2	-2	-2	-2
-2	-2	-2	-2
-2	-2	-2	-2
-2	-2	-2	-2
-2	-2	-2	-2
4	3	-2	-2
-2	-2	-2	-2
-2	-2	-2	-2
-2	-2	-2	-2
1	2	-2	-2
-2	-2	-2	-2
-2	-2	-2	-2
-2	-2	-2	-2
-2	-2	-2	-2
-2	-2	-2	-2
-2	-2	-2	-2
-2	-2	-2	-2
-2	-2	-2	-2
-2	-2	-2	-2
-2	-2	-2	-2
-2	-2	-2	-2
-2	-2	-2	-2
-2	-2	-2	-2
-2	-2	-2	-2
-2	-2	-2	-2

-2	-2	-2	-2
-2	-2	-2	-2
-2	-2	-2	-2
-2	-2	-2	-2
-2	-2	-2	-2
-2	-2	-2	-2
-2	-2	-2	-2
-2	-2	-2	-2
-2	-2	-2	-2
-2	-2	-2	-2
-2	-2	-2	-2
-2	-2	-2	-2
-2	-2	-2	-2
-2	-2	-2	-2
-2	-2	-2	-2
-2	-2	-2	-2
-2	-2	-2	-2
-2	-2	-2	-2
-2	-2	-2	-2
3	1	-2	-2
-2	-2	-2	-2
-2	-2	-2	-2
-2	-2	-2	-2
-2	-2	-2	-2
-2	-2	-2	-2
-2	-2	-2	-2
-2	-2	-2	-2
-2	-2	-2	-2
-2	-2	-2	-2
-2	-2	-2	-2
-2	-2	-2	-2
-2	-2	-2	-2
-2	-2	-2	-2
-2	-2	-2	-2
-2	-2	-2	-2
-2	-2	-2	-2
-2	-2	-2	-2
-2	-2	-2	-2
-2	1	-2	-2
-2	-2	-2	-2
-2	-2	-2	-2
-2	-2	-2	-2
-2	-2	-2	-2
-2	-2	-2	-2
2	1	-2	-2
-2	-2	-2	-2
-2	-2	-2	-2
-2	-2	-2	-2
-2	-2	-2	-2
-2	-2	-2	-2
-2	-2	-2	-2
-2	-2	-2	-2
-2	-2	-2	-2
-2	-2	-2	-2

22. (Access to clinical re	22. (In-depth underst	22. (Other
-2	-2	-2
-2	-2	-2
-2	-2	-2
-2	-2	-2
-2	-2	-2
-2	-2	-2
-2	-2	-2
-2	-2	-2
-2	-2	-2
-2	-2	-2
-2	-2	-2
2	3	-2
-2	-2	-2
-2	-2	-2
-2	-2	-2
-2	-2	-2
-2	-2	-2
-2	-2	-2
-2	-2	-2
-2	-2	-2
-2	-2	-2
-2	-2	-2
-2	-2	-2
-2	-2	-2
-2	-2	-2
-2	-2	-2
-2	-2	-2
-2	-2	-2
-2	-2	-2
-2	-2	-2
-2	-2	-2
-2	-2	-2
-2	-2	-2
-2	-2	-2
-2	-2	-2
-2	-2	-2
-2	-2	-2
-2	-2	-2
-2	-2	-2
-2	-2	-2
-2	-2	-2
-2	-2	-2
-2	-2	-2
-2	-2	-2
-2	-2	-2
-2	-2	-2
-2	-2	-2
-2	-2	-2
-2	-2	-2
-2	-2	-2
-2	-2	-2
-2	-2	-2
-2	-2	-2
-2	-2	-2
-2	-2	-2

-2	-2	-2
-2	-2	-2
-2	-2	-2
-2	-2	-2
-2	-2	-2
-2	-2	-2
-2	-2	-2
-2	-2	-2
-2	-2	-2
-2	-2	-2
-2	-2	-2
-2	-2	-2
-2	-2	-2
-2	-2	-2
-2	-2	-2
-2	-2	-2
-2	-2	-2
-2	-2	-2
-2	-2	-2
-2	-2	-2
-2	-2	-2
-2	-2	-2
-2	-2	-2
-2	-2	-2
-2	-2	-2
-2	-2	-2
-2	-2	-2
-2	-2	-2
-2	-2	-2
-2	-2	-2
-2	-2	-2
-2	-2	-2
-2	-2	-2
-2	-2	-2
-2	-2	-2
-2	-2	-2
-2	-2	-2
-2	-2	-2
-2	-2	-2
-2	-2	-2
-2	-2	-2
-2	-2	-2
-2	-2	-2
-2	-2	-2
-2	-2	-2
-2	-2	-2
-2	-2	-2
-2	-2	-2
-2	-2	-2
-2	-2	-2
-2	-2	-2
-2	-2	-2
-2	-2	-2
-2	-2	-2
-2	-2	-2
-2	-2	-2

-2	-2	-2
-2	-2	-2
-2	-2	-2
-2	-2	-2
-2	-2	-2
-2	-2	-2
-2	-2	-2
-2	-2	-2
-2	-2	-2
-2	-2	-2
-2	-2	-2
-2	-2	-2
-2	-2	-2
-2	-2	-2
-2	-2	-2
-2	-2	-2
-2	-2	-2
-2	-2	-2
-2	-2	-2
-2	-2	-2
-2	-2	-2
-2	-2	-2
-2	-2	-2
-2	-2	-2
-2	-2	-2
-2	-2	-2
-2	-2	-2
-2	-2	-2
-2	-2	-2
-2	-2	-2
-2	-2	-2
-2	-2	-2
-2	-2	-2
-2	-2	-2
-2	-2	-2
-2	-2	-2
-2	-2	-2
-2	-2	-2
-2	-2	-2
-2	-2	-2
-2	-2	-2
-2	-2	-2
-2	-2	-2
-2	-2	-2
-2	-2	-2
-2	-2	-2
-2	-2	-2
-2	-2	-2
-2	-2	-2
-2	-2	-2
-2	-2	-2
-2	-2	-2
-2	-2	-2
-2	-2	-2
-2	-2	-2
-2	-2	-2

-2	-2	-2
-2	-2	-2
-2	-2	-2
-2	-2	-2
-2	-2	-2
-2	-2	-2
-2	-2	-2
-2	-2	-2
-2	-2	-2
-2	-2	-2
-2	-2	-2
-2	-2	-2
-2	-2	-2
-2	-2	-2
-2	-2	-2
-2	-2	-2
-2	-2	-2
-2	-2	-2
-2	-2	-2
-2	-2	-2
-2	-2	-2
-2	-2	-2
-2	-2	-2
-2	-2	-2
-2	-2	-2
-2	-2	-2
-2	-2	-2
-2	-2	-2
-2	-2	-2
-2	-2	-2
-2	-2	-2
-2	-2	-2
-2	-2	-2
-2	-2	-2
-2	-2	-2
-2	-2	-2
-2	-2	-2
-2	-2	-2
-2	-2	-2
-2	-2	-2
-2	-2	-2
-2	-2	-2
-2	-2	-2
-2	-2	-2
-2	-2	-2
-2	-2	-2
-2	-2	-2
-2	-2	-2
-2	-2	-2
-2	-2	-2
-2	-2	-2
-2	-2	-2
-2	-2	-2
-2	-2	-2
-2	-2	-2
-2	-2	-2

-2	-2	-2
-2	-2	-2
-2	-2	-2
-2	-2	-2
-2	-2	-2
-2	-2	-2
-2	-2	-2
-2	-2	-2
-2	-2	-2
-2	-2	-2
-2	-2	-2
-2	-2	-2
-2	-2	-2
-2	-2	-2
-2	-2	-2
-2	-2	-2
-2	-2	-2
-2	-2	-2
-2	-2	-2
-2	-2	-2
-2	-2	-2
-2	-2	-2
-2	-2	-2
-2	-2	-2
-2	-2	-2
-2	-2	-2
-2	-2	-2
-2	-2	-2
-2	-2	-2
-2	-2	-2
-2	-2	-2
-2	-2	-2
-2	-2	-2
-2	-2	-2
-2	-2	-2
-2	-2	-2
-2	-2	-2
-2	-2	-2
-2	-2	-2
-2	-2	-2
-2	-2	-2
-2	-2	-2
-2	-2	-2
-2	-2	-2
-2	-2	-2
-2	-2	-2
-2	-2	-2
-2	-2	-2
-2	-2	-2
-2	-2	-2
-2	-2	-2
-2	-2	-2
-2	-2	-2
-2	-2	-2
-2	-2	-2
-2	-2	-2

-2	-2	-2
-2	-2	-2
-2	-2	-2
-2	-2	-2
-2	-2	-2
-2	-2	-2
-2	-2	-2
-2	-2	-2
-2	-2	-2
-2	-2	-2
-2	-2	-2
-2	-2	-2
-2	-2	-2
-2	-2	-2
-2	-2	-2
-2	-2	-2
-2	-2	-2
-2	-2	-2
-2	-2	-2
-2	-2	-2
-2	-2	-2
-2	-2	-2
-2	-2	-2
-2	-2	-2
-2	-2	-2
-2	-2	-2
-2	-2	-2
-2	-2	-2
-2	-2	-2
-2	-2	-2
-2	-2	-2
-2	-2	-2
-2	-2	-2
-2	-2	-2
-2	-2	-2
-2	-2	-2
-2	-2	-2
-2	-2	-2
-2	-2	-2
-2	-2	-2
-2	-2	-2
-2	-2	-2
-2	-2	-2
-2	-2	-2
-2	-2	-2
-2	-2	-2
-2	-2	-2
-2	-2	-2
-2	-2	-2
-2	-2	-2
-2	-2	-2
-2	-2	-2
-2	-2	-2
-2	-2	-2
-2	-2	-2
-2	-2	-2

-2	-2	-2
-2	-2	-2
-2	-2	-2
-2	-2	-2
-2	-2	-2
-2	-2	-2
-2	-2	-2
-2	-2	-2
-2	-2	-2
-2	-2	-2
-2	-2	-2
-2	-2	-2
-2	-2	-2
-2	-2	-2
-2	-2	-2
-2	-2	-2
-2	-2	-2
-2	-2	-2
-2	-2	-2
-2	-2	-2
-2	-2	-2
-2	-2	-2
-2	-2	-2
-2	-2	-2
-2	-2	-2
-2	-2	-2
-2	-2	-2
-2	-2	-2
-2	-2	-2
-2	-2	-2
-2	-2	-2
-2	-2	-2
-2	-2	-2
-2	-2	-2
-2	-2	-2
-2	-2	-2
-2	-2	-2
-2	-2	-2
-2	-2	-2
-2	-2	-2
-2	-2	-2
-2	-2	-2
-2	-2	-2
-2	-2	-2
-2	-2	-2
-2	-2	-2
-2	-2	-2
-2	-2	-2
-2	-2	-2
-2	-2	-2
-2	-2	-2
-2	-2	-2
-2	-2	-2
-2	-2	-2

areyourrequirements for the qualifications of the teaching faculty (multiple choice,

-3
1
1
-3
1
1
1
1
1
1
1
1
0
1
0
1
-3
0
-3
1
1
1
1
1
1
1
1
1
1
-3
1
0
1
-3
1
1
0
-3
1
1
1
1
1
1
1
1
1
1
-3
1
1
0
1
1
1

1
1
1
1
0
1
1
1
1
0
-3
1
1
1
1
1
1
1
1
0
-3
1
1
-3
0
1
-3
1
1
1
-3
-3
-3
0
1
-3
1
-3
1
0
-3
0
0
1
1
1
-3
1
0
1
1
1
1
1
-3
-3

1
1
1
-3
1
1
0
1
0
1
-3
1
1
1
1
-3
1
1
-3
1
1
1
1
0
1
0
-3
1
0
1
0
-3
-3
1
1
1
1
0
1
1
1
1
0
1
1
1
1
-3
-3
-3
1
-3
1
0
0
-3

0
1
1
1
1
0
-3
-3
0
1
0
0
0
-3
-3
0
1
0
1
1
0
-3
0
1
1
0
1
1
-3
-3
1
1
-3
0
1
1
0
1
-3
0
1
1
1
1
0
-3
1
1
1
1
1
1
1
1
1
1

1
1
1
0
1
1
1
1
-3
0
1
1
1
1
1
-3
-3
-3
1
-3
1
1
0
1
0
-3
1
0
1
0
0
1
1
-3
1
1
1
0
1
1
1
1
1
-3
1
1
1
1
1
-3
1
1
0
-3
1
-3

0
1
1
1
1
1
-3
-3
-3
0
0
1
1
1
-3
1
1
1
-3
0
1
1
1
1
0
1
1
1
1
0
1
-3
1
1
1
1
1
0
1
1
1
1
1
1
1
1
1
1
1
0
-3
0
1
-3
1
-3

1
-3
1
1
0
1
-3
1
1
-3
1
1
1
-3
1
0
0
1
1
1
1
-3
-3
-3
-3
-3
1
1
-3
1
1
-3
0
1
1
1
1
1
0
0
1
1
1
1
1
1
1
1
1
-3
-3
0
0
1

23. (Editor/Editorial Boarc	23. (Author of a published first-r	23. (Domestic hosting of clinical research
-3	-3	-3
0	0	1
1	1	1
-3	-3	-3
1	1	1
1	1	1
1	1	1
1	1	1
1	1	1
1	1	0
1	1	1
0	1	1
0	1	1
1	1	1
0	1	1
1	1	1
-3	-3	-3
0	1	1
-3	-3	-3
1	1	1
1	1	1
1	0	0
1	1	1
1	1	1
1	0	1
1	0	0
0	0	0
1	0	1
1	1	1
-3	-3	-3
1	1	1
1	0	1
1	1	1
-3	-3	-3
1	1	1
1	1	1
0	0	1
-3	-3	-3
1	0	0
1	1	1
1	0	1
1	1	1
1	1	1
1	1	1
1	0	1
1	1	1
1	0	1
0	0	1
-3	-3	-3
1	0	1
1	1	1
1	0	1
1	1	1
1	1	1
1	1	1

1	1	1
1	1	0
1	0	1
1	0	0
0	1	1
1	1	0
1	1	1
1	1	1
1	1	1
0	1	1
-3	-3	-3
1	1	1
1	1	0
1	0	0
1	1	1
1	1	1
1	0	0
0	0	1
0	0	1
0	1	0
-3	-3	-3
1	1	1
1	1	1
-3	-3	-3
0	1	1
1	1	1
-3	-3	-3
1	0	0
1	1	1
1	1	1
-3	-3	-3
-3	-3	-3
-3	-3	-3
1	1	1
1	1	1
-3	-3	-3
1	1	1
-3	-3	-3
1	1	1
1	1	1
-3	-3	-3
0	0	1
0	1	0
1	1	1
1	1	1
1	1	1
-3	-3	-3
1	1	1
0	1	1
1	1	1
1	1	1
1	1	1
1	1	1
1	1	1
-3	-3	-3
-3	-3	-3

1	1	1
1	0	1
0	1	1
-3	-3	-3
1	1	1
0	1	1
0	0	0
1	1	1
1	0	1
1	1	1
-3	-3	-3
1	1	1
1	1	1
1	1	1
1	1	1
-3	-3	-3
1	1	1
1	0	0
-3	-3	-3
0	0	0
1	1	1
0	1	0
1	1	1
1	0	1
1	1	1
1	1	1
-3	-3	-3
1	1	1
1	1	1
1	1	1
1	1	1
-3	-3	-3
-3	-3	-3
1	1	1
0	1	1
1	0	0
0	1	1
1	0	1
1	0	1
1	1	1
1	1	1
1	1	1
1	1	1
1	1	1
1	1	1
1	1	1
1	1	0
-3	-3	-3
-3	-3	-3
-3	-3	-3
0	1	0
-3	-3	-3
1	1	1
0	1	1
1	1	1
-3	-3	-3

0	1	1
1	1	1
1	1	1
1	1	1
1	1	1
1	1	0
-3	-3	-3
-3	-3	-3
1	0	1
0	0	0
0	0	0
0	0	1
0	1	1
-3	-3	-3
-3	-3	-3
0	1	1
1	0	0
0	1	1
1	1	0
1	1	1
0	1	1
-3	-3	-3
1	1	0
1	1	1
1	1	1
1	0	0
0	0	1
1	1	1
-3	-3	-3
-3	-3	-3
1	1	1
0	0	0
-3	-3	-3
0	0	0
1	1	1
0	0	1
0	1	1
1	1	1
-3	-3	-3
0	1	1
1	1	1
1	1	1
1	1	1
0	1	0
0	1	1
-3	-3	-3
1	1	1
1	1	1
1	1	1
1	1	0
1	0	1
1	1	1
1	1	1
1	1	0
1	1	1
1	1	1

1	0	1
1	1	1
1	1	1
1	1	0
1	1	1
1	1	1
1	1	1
0	0	1
-3	-3	-3
0	0	0
0	0	1
1	1	0
1	1	0
1	0	1
0	0	1
-3	-3	-3
-3	-3	-3
-3	-3	-3
1	1	1
-3	-3	-3
1	1	1
1	0	1
0	0	1
1	1	1
0	0	1
-3	-3	-3
1	1	1
1	0	1
1	0	0
0	0	1
1	0	0
0	1	1
0	1	1
-3	-3	-3
1	1	1
1	1	1
0	0	0
1	1	1
1	1	1
1	0	1
1	1	1
1	0	1
1	1	1
-3	-3	-3
1	1	1
1	1	1
0	0	1
0	1	1
0	0	0
-3	-3	-3
0	0	0
0	0	1
0	0	1
-3	-3	-3
0	0	1
-3	-3	-3

0	0	1
1	1	1
1	1	1
1	0	1
0	0	1
1	0	1
-3	-3	-3
-3	-3	-3
-3	-3	-3
0	0	1
0	0	1
1	1	0
0	1	1
1	1	1
-3	-3	-3
1	1	1
1	1	1
1	1	1
-3	-3	-3
0	1	1
1	1	1
1	0	1
1	1	1
1	1	1
1	1	1
1	1	1
1	1	1
1	1	1
1	0	0
1	1	1
1	1	1
-3	-3	-3
0	0	0
1	1	1
1	1	1
1	1	1
0	0	1
0	0	1
1	1	1
1	1	1
1	1	1
1	1	1
1	1	1
1	1	1
1	0	0
1	0	0
1	1	0
1	1	1
1	0	1
1	1	1
-3	-3	-3
0	1	1
1	1	1
-3	-3	-3
1	1	1
-3	-3	-3

1	1	1
-3	-3	-3
1	1	1
0	1	1
0	1	1
1	1	1
-3	-3	-3
1	1	1
1	1	1
-3	-3	-3
1	1	1
1	1	1
1	1	1
-3	-3	-3
1	1	0
1	1	1
1	1	0
1	1	1
0	0	1
0	0	1
1	1	1
-3	-3	-3
-3	-3	-3
-3	-3	-3
-3	-3	-3
-3	-3	-3
1	0	1
1	0	1
-3	-3	-3
1	0	0
1	1	0
-3	-3	-3
1	1	1
1	1	0
1	1	1
1	1	0
1	0	0
0	0	1
0	0	1
1	1	1
1	1	1
1	1	1
1	1	0
1	1	1
1	1	1
0	1	1
1	1	1
1	1	1
1	1	1
-3	-3	-3
-3	-3	-3
1	1	1
1	0	1
1	1	1

23. (Executives with extensive	23. (Other
-3	-3
1	0
1	1
-3	-3
1	0
1	0
1	0
1	0
1	0
0	0
0	0
1	0
0	0
1	0
1	0
0	0
-3	-3
0	0
-3	-3
1	0
0	0
0	0
1	0
1	0
0	0
1	0
0	0
1	0
1	0
-3	-3
1	0
1	0
0	0
-3	-3
1	0
0	0
1	0
-3	-3
0	0
1	0
1	0
1	0
0	0
1	0
0	0
0	0
1	0
1	0
-3	-3
1	0
1	0
1	0
0	0
1	0
1	0

1	0
0	0
0	0
1	0
1	0
0	0
1	0
1	0
1	0
1	0
-3	-3
1	0
1	0
0	0
0	0
1	0
0	0
1	0
1	0
0	0
-3	-3
0	0
1	0
-3	-3
0	0
0	0
-3	-3
0	0
1	0
1	0
-3	-3
-3	-3
-3	-3
0	0
1	0
-3	-3
1	0
-3	-3
0	0
0	0
-3	-3
1	0
0	0
1	0
1	0
1	0
-3	-3
1	0
0	0
1	0
0	0
0	0
0	0
1	0
-3	-3
-3	-3

1	0
1	0
1	0
-3	-3
1	0
0	0
1	0
1	0
1	0
1	0
-3	-3
1	0
0	0
1	0
1	0
-3	-3
1	0
0	0
-3	-3
0	0
0	0
0	0
1	0
1	0
1	0
1	0
-3	-3
1	0
0	0
1	0
1	0
-3	-3
-3	-3
0	0
0	0
0	0
1	0
1	0
0	0
1	0
1	0
0	0
0	0
1	0
0	0
1	0
0	0
-3	-3
-3	-3
-3	-3
0	0
-3	-3
1	0
0	0
0	0
-3	-3

1	0
1	0
1	0
1	0
0	0
1	0
-3	-3
-3	-3
1	0
0	0
1	0
1	0
1	0
-3	-3
-3	-3
0	0
0	0
0	0
0	0
1	0
0	0
-3	-3
0	0
1	0
1	0
0	0
1	0
1	0
-3	-3
-3	-3
0	0
0	0
-3	-3
1	0
1	0
1	0
0	0
1	0
-3	-3
1	0
0	0
1	0
1	0
1	0
0	0
-3	-3
1	0
1	0
1	0
0	0
1	0
1	0
1	0
0	0
1	0
1	0

1	0
0	0
1	0
0	0
1	0
1	0
1	0
1	0
-3	-3
0	1
1	0
0	0
1	0
1	0
1	0
-3	-3
-3	-3
-3	-3
1	0
-3	-3
0	0
1	0
1	0
0	0
1	0
-3	-3
1	0
0	0
0	0
1	0
0	0
1	0
1	0
-3	-3
1	0
1	0
1	1
0	0
1	0
0	0
1	0
1	0
1	0
-3	-3
0	0
1	0
1	0
0	0
1	0
-3	-3
0	0
1	0
1	0
-3	-3
1	0
-3	-3

1	0
1	0
1	0
0	0
1	0
0	0
-3	-3
-3	-3
-3	-3
1	0
1	0
0	0
0	0
1	0
-3	-3
0	0
1	0
0	0
-3	-3
0	0
1	0
1	0
0	0
1	0
1	0
0	0
1	0
0	0
0	0
0	0
1	0
-3	-3
0	0
1	0
0	0
1	0
1	0
0	0
0	0
0	0
1	0
0	0
1	0
1	0
0	0
0	0
0	0
1	0
0	0
0	0
-3	-3
0	0
0	0
-3	-3
1	0
-3	-3

1	0
-3	-3
1	0
1	0
1	0
1	0
-3	-3
0	0
1	0
-3	-3
0	0
1	0
0	0
-3	-3
0	0
0	0
1	0
1	0
1	0
1	0
1	0
-3	-3
-3	-3
-3	-3
-3	-3
-3	-3
0	0
1	0
-3	-3
0	0
0	0
-3	-3
0	0
0	0
1	0
0	0
0	0
0	0
1	0
1	0
1	0
1	0
0	0
0	0
1	0
0	0
1	0
0	0
0	0
-3	-3
-3	-3
0	0
0	0
1	0

24. Do you have any suggestions for upcoming training programs? We sincerely appreciate
-3
not have
not available
-3
not have
not have
Would like the content to be systematized
Hopefully it will be open for free viewing
It is hoped that trainable instructors will come from a wide range of fields
There are a lot of clinical rct research courses out there. And only a few large hospitals have the capacity to co
I hope to present methods and examples of clinical research utilizing big data methods and clinical databases
Widely publicize the project
I wish it was more hands-on.
not have
not have
Specific guidance from a practical perspective
-3
not have
-3
Early release of preview content
not have
not have
I hope to be able to have the class online and be able to go back to it over and over again
Just started a clinical study, would like more training
Learning and practicing at the same time
not have
Turn it on as soon as possible
not have
It is recommended that there be an overall description of the intervention study.
-3
Ongoing connections can be made
I have the honor to publish an article in NEJM, and I am also a big fan of your public website. I hope that more
The focus should be on case-controls, case series, and other types of study designs that are feasible for most
-3
Serious engagement in cooperation
Combined with hospital specialties
Clinical Research Thinking and Clinical Research Management
-3
Very much looking forward to it. Through the training, I will be able to conduct high quality research and publish
affordable
No specifics at this time
certified
Is it possible to suggest opening up the class? In particular, the potential for advancement is greater for medica
With practical examples and simulation exercises
not have
not have
Preferably focused on the grass roots
Able to explain specific cases with current and up-to-date research, and be able to speak a little more plainly al
-3
Code of Practice for Clinical Trials and Ethical Management of Cell Therapy and Gene Therapy
not available
I hope to accommodate the inconvenient timing of clinical scheduling on-call transfers. Thank you very much!
not have
not have
Research

Very much looking forward to attending
not available
Wish the course was more in-depth rather than a brief introduction
Desire to connect and mentor with top international teachers
not have
No more
RCT statistical protocol design details: from the very beginning sample size POWER, randomization protocol s
Hierarchical training
How do I enroll in the study?
Ground yourself.
-3
It is recommended to differentiate between types of clinical studies, e.g. infectious diseases, chronic diseases,
Lower tuition fees
not have
not have
There is a lot of basic training out there, and I hope to improve the depth.
not have
The price is not too high
not have
not have
-3
I hope it's on-line. I've been out of town lately.
not have
-3
Recommendations are designed to fit the knowledge level of primary care physicians in China.
If the course format is offline, I hope it can cover second-tier cities; if the course is online, I hope it can strength
-3
Integration of theory and practice
Hopefully it will involve meta-analysis and drug RCTs for rare diseases
not have
-3
-3
-3
I hope the fees are reasonable and not too high
Integrate more examples
-3
Online, replayable
-3
5
N/A
-3
not have
not available
We hope that we can really learn useful knowledge from the training program and enrich our experience and a
Thematic approach
Theory combined with practice
-3
Specialized training on the separation of drug, device and IVD clinical trials.
Beginning, enduring, long term
expectation
Start from scratch, suitable for the grassroots, and talk about some submission techniques and journal selectic
not have
Practical and practical
1. provide research examples, conduct case studies and group discussions. 2. provide policy interpretation for
-3
-3

Focus on participation
I hope there are interpretations of classic and latest cases, in addition, the affordable tuition will surely warm th
Emphasize hands-on training.
-3
Anyone wishing to train can sign up
I hope there will be training for people who have no basic knowledge at all.
Needs to be in line with China's national context, the current state of clinical research execution
not have
not have
not available
-3
A list of course content could be made and participants could select a number of courses that are of most inter
not have
not have
One-on-one mentorship with specific guidance in completing a clinical research protocol
-3
not available
More classic studies explained
-3
persuade
not available
NA
expectation
not have
Hands-on, online flexible learning
NA
-3
not have
not have
Hopefully there will be hands-on programs, or open feedback channels where problems can be resolved in a ti
linking theory with practice
-3
-3
Looking forward to it.
not have
If possible, it is recommended that additional expert lectures from the Oxford University Clinical Trials Center b
Highly practical
Domestic field of innovative devices heats up, wants to add courses in devices
below the line
Obtaining the appropriate training certificates
not have
not have
Hope well-known journal editorial board members and practical people lectures, time weekend schedule suitab
Teaching more research examples
not have
Case studies based on published articles
patronize
-3
-3
-3
not have
-3
not available
Principles + examples
Follow-up long-term guidance
-3

not have
not have
thank you
not have
In-depth explanations, close to the practice, to avoid generalizations
not have
-3
-3
practicality
It should be global project
Real-world face to marketization
How to synthesize regulatory requirements in study design
not have
-3
-3
Reaching the grassroots, suiting the grassroots, deepening cooperation
The final assessment can be in the form of a collaborative group paper or a proposal.
not have
Focus on practical needs
Emphasis on hands-on
not have
-3
Transformation of scientific research projects
It is recommended that a practical course in statistical analysis programs be added that is actionable and grou
Let's hope it goes as planned.
solid progress
not have
Would like to see more flexibility in training hours
-3
-3
combining practical aspects
not have
-3
not have
hasn't
not have
not have
What's the price? I hope it's affordable.
-3
not have
Come on, come on.
Hands-on plus coaching
not have
not have
pragmatic
-3
not have
Thank you for conducting such a program, and I hope that more sessions will be held, with a combination of Cl
not have
Turn it on.
Suggest short-term training courses or weekend classes, offline teaching, without interfering with daily work
As much as possible, taking into account busy clinical schedules, classes are held online and can be watched
You can record and set up a Q&A sharing group after the class.
Lessons can be watched back. Q&A opportunities are available for participants.
Follow-up instruction continues after the class
There is a follow-up mentoring group after the class for research mentoring and communication

not have
not have
not have
On line, you can play it back.
not have
I hope to learn.
not have
Suggesting the development of training programs for corporate medical personnel or the establishment of a pla
-3
Avoid empty talks
not available
not have
No
Held online
not have
-3
-3
-3
I hope to present the impact of FDA regulations on clinical study design and introduce some innovative clinical
-3
Would like to see some training in clinical research for the medical statistics profession.
not have
Would like to schedule some introductory classes, as well as strategy classes for clinical development
I hope there will be more opportunities for interaction
You can have one topic or module per issue and cover the content in detail instead of fast-food filler.
-3
Facing the Nanjing area
not have
not have
Fee: 500 RMB per credit hour
。
Taught in different modules
Looking to learn more about new design ideas
-3
He attended the "Qixing Star" clinical research lecture hall of the Chinese Medical Association at the time of his
With the development of the country in mind, the people are in danger and the benefits will come naturally. Be
Case-centered learning and discussion
Targeted counseling
English (language)
not have
More dry goods
not available
It is hoped that clear examples will be given to guide realistic and feasible paths in response to the current situa
-3
It's all about the content, not the content.
As soon as possible
1. suggested that the presentation could be more integrated with specific cases; 2. suggested that historical fut
Expand your audience with a combination of paid and free.
Researchers who have actually designed, and implemented clinical trials are not full of speakers.
-3
Integration of theory and practice
The time is flexible, and there should be some thematic offline interactive and practical activities to solve the pr
Desire to learn high quality, standardized clinical knowledge
-3
More actual desensitization case studies to share
-3

I'd like to be able to cover all the bases, let them flow through me.
not have
not have
not have
not available
How well researcher-initiated research
-3
-3
-3
not have
hasn't
Very willing to participate
real combat
The design of clinical research protocols and methods of statistical analysis of data can hopefully be explained
-3
not have
Serving Clinicians
Hoping to offer classes in multiple locations
-3
Desire to care for a clinical frontline with a rostered schedule and variable weekly availability
Conducting a course on data analytics hopefully easy to understand
Practical case discussions
not have
It is recommended that a feasible schedule of lectures be developed based on the realities of a wide range of h
More hands-on case study explanations will help understanding to get started.
Can the registration fee be waived
Field training and exchanges at renowned clinical medical research centers at home and abroad
Great content, looking forward to the training series!
not have
not have
not have
-3
Courses can be modularized to accommodate learners with different needs
Essentials and techniques of clinical design programs
not have
Looking forward to it.
Close to the current industry
not available
not available
not available
Combined with examples
not have
Looking forward to looking forward
Strengthening training for grass-roots personnel with a weak research base
not have
5
It is recommended that the design be more diverse in type, avoiding a predominance of oncology-based studie
Towards young researchers
not have
Desire to incorporate specific cases
-3
It's cheaper.
You can ask the teacher for advice after class.
-3
Suggested to be combined with examples
-3

I can apply to be a lecturer
-3
We hope to set up a training program based on China's national conditions and suitable for all levels.
Avoid only theory, it is recommended to explain with examples, with raw data, with software operations (code),
Hopefully, it will take into account small doctors with weak foundations
What about the cost?
-3
Training based on clinical research programs
Starting from examples
-3
More content on design and thinking of clinical research projects.
not have
Don't overcharge.
-3
not have
Participants combine practical and theoretical aspects of learning to complete the design and program writing c
Minor specialty courses for minor specialties such as anesthesiology, please
not have
Hopefully there will be a replay of the training
We hope that we can understand the process of clinical trials from design to execution to supervision from ente
Increase advocacy to reach more people
-3
-3
-3
-3
-3
Moderate cost
not have
-3
free (of charge)
not have
-3
connect with the local community
Explained with practical examples
There could be one-on-one opportunities
Evaluate the resources currently available to the learner and give direction recommendations for feasible clinic
Novel Clinical Study Designs
not have
not have
not available
Recording is recommended to be able to learn over and over again
not available
not have
More hands-on, real-life case-based sharing and discussion
It might be better to look at case studies and explain them with examples.
I hope to be able to practice and receive guidance on clinical trial design based on what I have learned
Guidance on experimental design
Desire to expand audience
volunteer health or relief work
-3
-3
not available
not have
Don't pay too much.

25. Name
-3
(empty)
(empty)
-3
(empty)
Hu Yajuan (1989-), Chinese athlete, lady high jumper
Huo Shaofeng (1936-), general secretary of the PRC Supreme Court 1998-2003
Cheng Yu
Ivan (Russian name)
Ankang prefecture level city in Shaanxi
Benin Wong
bin
Fang Fang (1948-), one of the leaders of the Beijing student democracy movement of 1989
Liu Xiaolu (1968-), PRC politician
Wei Gongpei (1943-), poet poetical leader of the Ming dynasty
Lei Chong
-3
Li Jian (1540-1562), Ming dynasty novelist
-3
Fang Xiaoyan
Wang Yi
Yan Yan
Zhao Lidong
Li Nan Nan
(empty)
He Yan
Jeffrey.
Lu Jinshan (1881-1943), Chinese poet
(empty)
-3
(empty)
Zhao Jiyu (1928-), Chinese actor
Li Xiang (1907-1989), Chinese communist leader, prime minister of the Republic of China after the cult
-3
infrastructure development
Duana (name)
An Jianghong
-3
Wang Mingwei (1976-), Taiwanese singer and actor
(empty)
(empty)
Tang Yong (1937-), Chinese army leader
thousands of forests
Wang Ying
(empty)
(empty)
Thorny Hing Keung (1935-), Chinese-American mathematician, precursor of Deng Xiaoping
He Yan
-3
Han Nan
Amy Li
Jiang Ruowen (1966-), sixth governor of Hainan
Liu Yongping (1973-), table tennis player, several times world and Olympic winner
(empty)
Wang Tianlu (1910-1998), Taiwanese master puppeteer

Rachel Lee (1975-), US pop singer
Xu Xiaolei
Yoyo Lau (1975-), Hong Kong actress
(empty)
(empty)
Cynthia
Zhang Zhongfan (1907-1984), Chinese communist leader, a martyr of the anti-Qing revolution
(empty)
Duan Xiaonan
(empty)
-3
Michele Chau
Jeff.
(empty)
Wu Dongqiao or Wu Dongzhi (1963-), Chinese-American writer, envoy of Southern China to Hong Kon
Anna Wang
(empty)
Wenying Chang
fu zhen
(empty)
-3
A shepherd boy never loses his way.
(empty)
-3
Yang Shuo (1935-), Chinese writer and poet
Wynter
-3
(empty)
(empty)
Sure
-3
-3
-3
(empty)
(empty)
-3
Liu Tonyuk (died c. 724 AD)
-3
Anglo-Chinese Exchange (ACE), Hong Kong philanthropic organization
(empty)
-3
(empty)
keep sth. confidential
Schmalfeldt (name)
Qian Yangyang
Yoyo Lau (1975-), Hong Kong actress
-3
(empty)
(empty)
Zhou Xinci (1915-1998), Mao Zedong's second wife
Yu Jianyu
Liu Jiaxin (1970-), PRC diplomat
Fan Tongtao
Shi Peng (1928-), leading PRC politician, prime minister 1987-1998
-3
-3

bells
(empty)
(empty)
-3
Wang Yuan (1979-), Taiwanese pop star
think
(empty)
Yin Yulian
Liu Xiankun
Shijunnan (name)
-3
Xu Chengfu (1938-), president of the PRC Supreme Court
Xu Hao
Wang Yan
Anna (person's name)
-3
Jiang Kai
Huang Hang (1944-), PRC politician, prime minister from 2008
-3
(empty)
He Wan Ying
(empty)
Wang Hanylu (1955-), Taiwanese actress
Xu Guangnan
Kathy
(empty)
-3
Sit Chung Yu
Wu Peixia
(empty)
keep sth. confidential
-3
-3
(empty)
lumpy
Liu Jing (1902-1987), Chinese writer
Yu Shan Dong (1941-), Chinese businessman and politician, prime minister 1990-2004
(empty)
Yang Zi (1916-), PRC politician
Liu Shubin
person from Beijing University
Zhang Huijie
Wu Aizi (1902-1994), Chinese communist leader, prime minister 1949-1990
horse or cavalry piece in Chinese chess
(empty)
Zhang Xinze (1908-1995), Chinese communist leader, a martyr of the Cultural Revolution
Sun Jade (jade ear-plug)
-3
-3
-3
(empty)
-3
Cao Yajing
Yang Shuhe (1938-), PRC diplomat and lawyer
Mr. Lee
-3

(empty)
Sean Zhang
Dr. Tian
Joseph Wang Lin-yao (1902-1998), Chinese pioneer nuclear physicist
Dr. Zhen
(empty)
-3
-3
Lee Ming
Zhang Shengjun
Tang Hongliang
Wang Honghui (1964-), Taiwanese actress
(empty)
-3
-3
Zou Luping (1885-1905), a martyr of the anti-Qing revolution, died in jail in 1905
Ma Chenhao
Tingting Zhang
Dr. Jiang
topaz (aluminum fluorosilicate)
youths
-3
Zhang Yongping (1973-), table tennis player, several times world and Olympic winner
Yu Kuai (1907-1984), Mongolian national poet
Klossi
(empty)
(empty)
Jin Weitao
-3
-3
(empty)
thank you
-3
Wang Fuxiu (1889-1910), Mao Zedong's second wife
roc (mythical bird of prey)
(empty)
(empty)
Wang Yongle (1907-1982), Chinese communist leader, a martyr of the Cultural Revolution
-3
Chu Kay
Strife
Xu Jinfu
Wang ZHH
Xiao Hibiscus (1933-), vice-president of the PRC Supreme Court 1998-2003
Li Kei (1982-), Taiwanese politician, prime minister 2007-2010
-3
(empty)
Yan Nan
(empty)
Li Bo
(empty)
Guo Yanying
Sun Jun (1933-), PRC army leader
Liu Huanxian
Chen Yingying
Chen Yingying

Li Na (1982-), Chinese tennis player, first Asian player win a Grand Slam (Gala)
(empty)
(empty)
Yang Qilin
Wayne Chang (1949-), Chinese US film director
Chen Cheng (1905-1975), communist leader and economist
Jiao Guohui (1931-1930), Chinese-American linguist
(empty)
-3
Bainian Feng
Miao Bolognese
(empty)
Samuel Kuok
(empty)
Zhang Xiao侠 (1930-), PRC film director
-3
-3
-3
(empty)
-3
(empty)
Xiang Si Long (1974-), Chinese-American physicist, astronomer and mathematician
(empty)
Li Si Cheng (1913-1987), Chinese-American physicist, Nobel laureate
(empty)
-3
(empty)
(empty)
Tian Changgeng (1910-1985), Chinese poet
(empty)
(empty)
(empty)
Fu Jing (1917-1996), Chinese communist leader, a martyr of the Cultural Revolution
-3
Qiu Xiaotong
(empty)
Caroline Chang (1962-), Taiwanese actress
Huang Guobao (1892-1975), noted scholar and political activist
(empty)
Nieaichi
(empty)
(empty)
(empty)
-3
Song Yihang (1902-1995), Northern Song dynasty poet
cj
(empty)
(empty)
David Shaw
-3
Liu Xing (1969-), Chinese astronomer who catalogued nebulae and clusters of stars
Liu Kang (1948-), PRC writer
Luo Peng (1928-), leading PRC politician, prime minister 1987-1998, reportedly leading a conservative
-3
ivy
-3

Wang Yun (died 409) emperor of Northern or Later Yan dynasty
Susan Niu
Li Xiaodong (1965-), PRC film director
Li Zhu Bin
(empty)
Huang Yulun
-3
-3
-3
(empty)
Chen Junfeng (1936-), Chinese chess grandmaster
Julia Wang
(empty)
Xiao Ruifeng (1936-), former president of the PRC Supreme Court 1998-2008
-3
(empty)
Julia Wong
sift
-3
Jiang Ruowen (1966-), sixth governor of Hainan
(empty)
(empty)
Xia Chengdong (1944-), Chinese businessman and politician, prime minister 1997-1998
(empty)
Poon Hong Nam
Zhu Xiaofei (1970-), PRC actress
Wu Clang
Dr. Lu
Li Fenglin (1934-), calligrapher and writer, author of many novels about the Ming dynasty
Chen Guang
Wu Zijun (1936-), PRC actress
-3
(empty)
ginger and red ginger
(empty)
LAM KIT TO
(empty)
(empty)
(empty)
(empty)
Wang Hui (1962-), Taiwanese actress
(empty)
Wang Weiqing
Liu Sizhong
Mr. Yu
Yang Yong (1974-), Chinese-American physicist, 1965 Nobel laureate
Liu Yi
name of states in Southern China at different historical periods
(empty)
Yang Jin (1916-), PRC politician
-3
CUCKOO
Cai Jie (1979-), Chinese dissident, one of the leaders of the Chinese communist party
-3
(empty)
-3

Tang Yuzhe
-3
(empty)
Pan Jun (1962-), Taiwanese poet
Yang Shuo (1935-), Chinese writer and poet
Kwok Lan Yin
-3
(empty)
(empty)
-3
Zheng Guibin (1943-), right-wing Japanese cabinet minister and prominent denier of Japanese war crin
(empty)
(empty)
-3
Yang Xinglong
(empty)
Mr. Tu Xiaokang (Taiwan Minister of Foreign Affairs)
(empty)
(empty)
glistening
Li Jin (1875-1907), famous Chinese female martyr
-3
-3
-3
-3
-3
(empty)
(empty)
-3
Liu Jue (1926-1992), female revolutionary leader of the Cultural Revolution
(empty)
-3
(empty)
(empty)
(empty)
plateaus
Zheng Xue Ying
Hou Guangsen (1938-), PRC politician, prime minister 1987-1998
Duan Benqing
(empty)
Wang Kuiji (1929-), Mao Zedong's second wife
Wei Xing (1956-), Chinese dissident, one of the leaders of the Beijing student democracy movement of
Director Bian
(empty)
(empty)
Fang Xiaoyan
Chi Songyuan (1906-1995), Taiwanese poet and writer
Lau Mei Shum
(empty)
-3
-3
(empty)
Zheng Yueh-Lan (1926-), Chinese-American mathematician, astronomer, and mathematician in the Mi
Lukoilin (name)

ural revolution

development
